# Synthesis of new pyrrolidine-based organocatalysts and study of their use in the asymmetric Michael addition of aldehydes to nitroolefins

**DOI:** 10.3762/bjoc.13.59

**Published:** 2017-03-27

**Authors:** Alejandro Castán, Ramón Badorrey, José A Gálvez, María D Díaz-de-Villegas

**Affiliations:** 1 Instituto de Síntesis Química y Catálisis Homogénea (ISQCH), CSIC - Universidad de Zaragoza, Departamento de Química Orgánica, Pedro Cerbuna 12, E-50009 Zaragoza, Spain

**Keywords:** enantioselective synthesis, Michael addition, organocatalysis, pyrrolidines, synthetic methods

## Abstract

New pyrrolidine-based organocatalysts with a bulky substituent at C2 were synthesized from chiral imines derived from (*R*)-glyceraldehyde acetonide by diastereoselective allylation followed by a sequential hydrozirconation/iodination reaction. The new compounds were found to be effective organocatalysts for the Michael addition of aldehydes to nitroolefins and enantioselectivities up to 85% ee were achieved.

## Introduction

In the first decades of the 21st century, the enantioselective organocatalysis has witnessed a tremendous development [1–4] and it is now considered to be the third pillar of enantioselective catalyses together with metal complex-mediated catalysis and biocatalysis. Among the different structures usually found in organocatalysis, the five-membered secondary amine structure of pyrrolidine has proven to be a privileged motif [5] with a powerful capacity in aminocatalysis [6–10]. In this context diarylprolinol silyl ethers have proven to be extremely efficient organocatalysts for a wide variety of chemical transformations [11].

In the course of our research we have been involved in the synthesis of new tuneable catalytic motifs to be used in organocatalysis starting from the chiral pool. Highly modular chiral aminodiol derivatives were obtained by the addition of organometallic reagents to chiral imines derived from (*R*)-glyceraldehyde – which is easily accessible from D-mannitol – and these were evaluated as chiral organocatalysts in the enantioselective α-chlorination of β-ketoesters, with excellent results obtained after optimisation of the organocatalyst structure [12].

In an effort to identify new, easily accessible and tuneable organocatalysts with the privileged pyrrolidine motif from the chiral pool, we have now focused on the synthesis of new chiral pyrrolidines capable of creating a sterically demanding environment due to the presence of a bulky 2,2-disubstituted-1,3-dioxolan-4-yl moiety at C2 from chiral imines derived from (*R*)-glyceraldehyde. The Michael addition of aldehydes to nitroolefins was selected as a model reaction to evaluate the effectiveness of the new pyrrolidine-based organocatalysts in aminocatalysis.

## Results and Discussion

We reasoned that pyrrolidines of type **C** with a bulky 2,2-disubstituted-1,3-dioxolan-4-yl moiety at C2 could provide the appropriate environment to lead to high levels of enantioselectivity in asymmetric transformations in which enamine intermediates are formed. The substituent R^1^ in the 1,3-dioxolane moiety in pyrrolidines **C** could be varied to modulate the reactivity and selectivity of the new organocatalysts.

**Figure 1:**
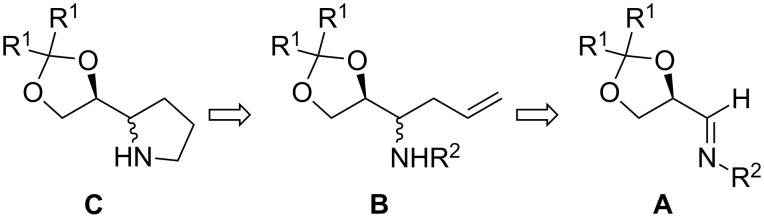
Strategy for the preparation of 2-substituted pyrrolidines **C**.

The sequential hydrozirconation/iodination of chiral homoallylic amines has been described as a straightforward approach to enantiomerically pure 2-substituted pyrrolidines [13–14]. Therefore we decided to test this methodology to gain access to the pyrrolidine ring in compound **C**. The required chiral homoallylic amines **B** can be easily obtained by the addition of allylmagnesium bromide to imines derived from (*R*)-glyceraldehyde acetonides **A** (Figure 1) according to our previously described methodology [15]. The configuration at C2 of the pyrrolidine ring would be determined in the diastereoselective allylation of the starting chiral imines.

**Scheme 1:**
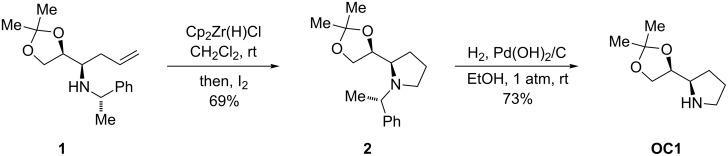
Synthesis of the new organocatalyst **OC1**.

The homoallylic amine **1** with *syn*-configuration was obtained by the reaction of the corresponding imine with allylmagnesium bromide as previously described [15]. The amine **1** was reacted with the Schwartz reagent in CH_2_Cl_2_ at room temperature to afford the hydrozirconated intermediate, which was immediately treated with iodine to yield *N*-benzylpyrrolidine **2** in 69% isolated yield. The subsequent exposure of compound **2** to molecular hydrogen in the presence of Pd(OH)_2_/C as a catalyst afforded the desired organocatalyst **OC1** in 73% yield (Scheme 1).

**Scheme 2:**
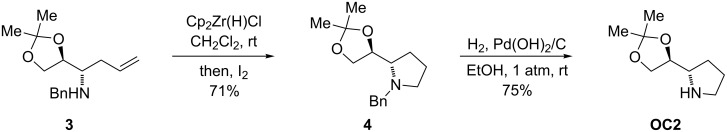
Synthesis of new organocatalyst **OC2**.

The same reaction sequence led to organocatalyst **OC2** in 52% overall yield starting from homoallylic amine **3** having *anti*-configuration, which was obtained by reaction of the corresponding BF_3_·OEt_2_ pre-complexed imine with allylmagnesium bromide as previously described [15] (Scheme 2). It is worth mentioning that the starting homoallylic amines **1** and **3** can be obtained on a multigram scale from the chiral pool.

**Scheme 3:**
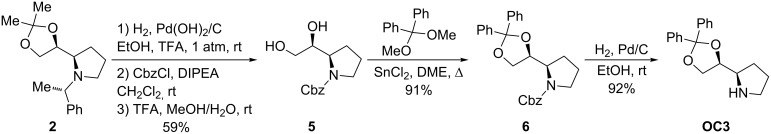
Synthesis of new organocatalyst **OC3**.

**Scheme 4:**
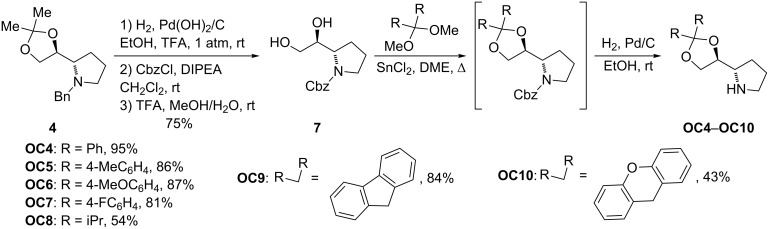
Synthesis of new organocatalysts **OC4**–**OC10**.

In order to obtain a series of new organocatalysts with substituents of different sizes and stereoelectronic properties in the dioxolane moiety, the following reaction sequence was applied to compounds **2** and **4**: a) N-deprotection by hydrogenolysis of the benzylic group with molecular hydrogen using Pd(OH)_2_/C as a catalyst in the presence of trifluoroacetic acid, b) reprotection of the amino group as benzylcarbamate by treatment of the crude reaction mixture with benzyl chloroformate in the presence of diisopropylethylamine, c) hydrolysis of the dioxolane moiety with trifluoroacetic acid, d) reconstruction of the dioxolane moiety by reaction of the diol with the corresponding dimethoxyacetal in the presence of SnCl_2_ and e) N-deprotection of the pyrrolidine by exposure of the benzylcarbamate to molecular hydrogen in the presence of catalytic Pd/C. In this way organocatalysts **OC3**–**OC10** were obtained (Scheme 3 and Scheme 4).

**Scheme 5:**
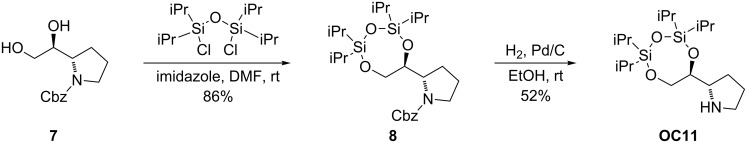
Synthesis of new organocatalyst **OC11**.

In addition another new organocatalyst, **OC11**, with a different bulky substituent at C2 in the pyrrolidine moiety was prepared. Reacting diol **7** with 1,3-dichlorotetraisopropyldisiloxane in the presence of imidazole and subsequent hydrogenolysis of the benzylcarbamate with molecular hydrogen in the presence of catalytic Pd/C (Scheme 5) afforded **OC11** in 43% overall yield for the two steps.

With this series of pyrrolidines at hand, the well-established Michael addition of aldehydes to nitroolefins [16–18] was selected as a benchmark reaction to study their behaviour as organocatalysts. Compounds with a related structure prepared from proline by Diez et al. have proven to work well as organocatalysts in the Michael addition of cyclohexanones to nitrostyrenes [19–20].

We first tested organocatalysts **OC1**–**OC4** in the reaction of *trans*-β-nitrostyrene with 3-phenylpropionaldehyde in order to determine the influence that the relative configuration of the pyrrolidine had on the results (Table 1). The reaction was initially carried out at room temperature in the presence of 10 mol % of the catalyst and using CH_2_Cl_2_ as solvent. Under these conditions the yield of the Michael adducts was 95–99% within 7 hours. The diastereoselectivity was moderate (dr = 70:30–78:22) in favour of the *syn-*diastereoisomer and enantioselectivites were ee ≈ 68% for the *syn-*adducts and ee = 44–63% for the *anti-*adducts. The stereochemistry of the major compound depended on the stereochemistry of the organocatalyst and Michael adducts of opposite configuration were obtained on using *syn* or *anti*-pyrrolidines with similar levels of enantioselectivity for the major *syn*-diastereoisomer.

**Table 1:** Initial screening of catalysts for the Michael addition of 3-phenylpropionaldehyde to *trans-*β-nitrostyrene.^a^

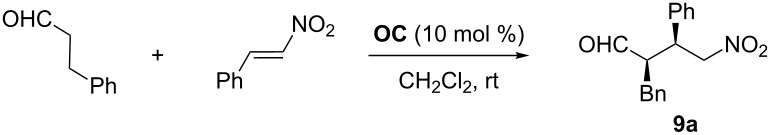					

Catalyst	*t* (h)	Yield^b^ (%)	*syn*:*anti*^b^	ee *syn*^c^ (%)	ee *anti*^c^ (%)

**OC1**	7	95	70:30	−68	−63
**OC2**	7	97	78:22	68	46
**OC3**	7	99	74:26	−68	−44
**OC4**	7	96	77:23	66	44

^a^Reaction performed in CH_2_Cl_2_ (2 mL) at room temperature using 0.2 mmol of β-nitrostyrene, 0.4 mmol of 3-phenylpropionaldehyde and 10 mol % of catalyst. ^b^Determined from the crude reaction mixture by ^1^H NMR spectroscopy using 1,3,5-trimethoxybenzene as internal standard. ^c^Determined by chiral HPLC.

Next, the effect of the solvent and temperature was studied using **OC4** as the organocatalyst (Table 2). The best results were obtained with methylcyclohexane as the solvent at 0 °C reaction temperature. Under these conditions after 24 h the reaction yield was high (87%), the observed diastereoselectivity was 92:8 in favour of the *syn-*adduct and the enantioselectivity reached 85% ee for the major *syn-*adduct. A further decrease in temperature did not improve these results substantially but did diminish the reaction yield (Table 2).

**Table 2:** Optimization of the reaction conditions for the Michael addition of 3-phenylpropionaldehyde to *trans*-β-nitrostyrene using catalyst **OC4**.^a^

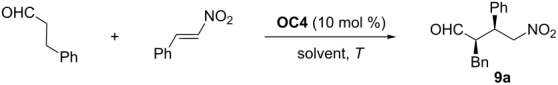						

Solvent	*T* (°C)	*t* (h)	Yield^b^ (%)	*syn*:*anti*^b^	ee *syn*^c^ (%)	ee *anti*^c^ (%)

CH_2_Cl_2_	rt	7	96	77:23	66	44
THF	rt	7	86	80:20	71	51
toluene	rt	7	82	84:16	74	44
CHCl_3_	rt	7	89	78:22	57	43
EtOH	rt	7	85	76:24	62	21
cyclohexane	rt	7	87	86:14	81	67
MTBE	rt	7	96	87:13	63	35
MeCN	rt	7	87	77:23	57	23
CF_3_C_6_H_4_	rt	7	93	89:11	78	75
C_6_F_6_	rt	7	90	92:8	80	62
C_6_F_11_CF_3_	rt	7	85	68:32	76	74
C_10_F_8_	rt	7	82	73:27	76	73
methylcyclohexane	0	24	87	92:8	85	58
toluene	0	24	84	86:14	80	39
methylcyclohexane	−20	24	77	94:6	85	40

^a^Reaction performed using 0.2 mmol of β-nitrostyrene, 0.4 mmol of 3-phenylpropionaldehyde and 10 mol % of **OC4** in the given solvent (2 mL). ^b^Determined from the crude reaction mixture by ^1^H NMR spectroscopy using 1,3,5-trimethoxybenzene as internal standard. ^c^Determined by chiral HPLC.

The organocatalysts **OC1**–**OC11** were then screened to reveal the influence of the substituent R attached to the dioxolane moiety on the reaction outcome and the results are collected in Table 3. However, the variation of this substituent did not result in any significant improvement of the diastereo- or enantioselectivity and based on these results organocatalyst **OC4** was found to be the most efficient and stereoselective organocatalyst. It is worth mentioning that on reducing the catalyst loading to 5 mol % the reactivity remained good and the diastereoselectivity and enantioselectivity were only slightly affected.

**Table 3:** Screening of organocatalysts **OC1**–**OC11** for the Michael addition of 3-phenylpropionaldehyde to *trans*-β-nitrostyrene.^a^

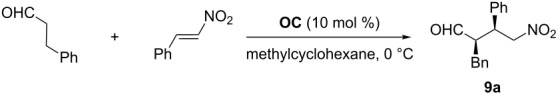				

Catalyst	Yield^b^ (%)	*syn*:*anti*^b^	ee *syn*^c^ (%)	ee *anti*^c^ (%)

**OC1**	84	84:18	77	72
**OC2**	77	94:6	81	50
**OC3**	91	78:22	77	65
**OC4**	87	93:7	85	58
**OC5**	99	92:8	84	63
**OC6**	86	88:12	80	61
**OC7**	90	92:8	83	57
**OC8**	91	93:7	73	70
**OC9**	93	93:7	76	n.d.
**OC10**	83	93:7	85	n.d.
**OC11**	72	87:13	63	n.d.
**OC4**^d^	81	89:11	82	59
**OC4**^e^	23	78:22	82	45

^a^Reaction performed using 0.2 mmol of β-nitrostyrene, 0.4 mmol of 3-phenylpropionaldehyde and 10 mol % of catalyst in methylcyclohexane (2 mL) at 0 °C for 24 h. ^b^Determined from the crude reaction mixture by ^1^H NMR spectroscopy using 1,3,5-trimethoxybenzene as internal standard. ^c^Determined by chiral HPLC. ^d^Catalyst loading 5 mol %. ^e^Catalyst loading 2 mol %.

It has been reported that additives present in the reaction medium can lead to improved results without changing other reaction conditions [21]. For example, in secondary amine-catalysed asymmetric reactions a Brønsted acid additive was found to accelerate the formation of the enamine intermediate and thus to improve not only the reactivity but also the diastereoselectivity and enantioselectivity [22–23]. On the other hand, the presence of thiourea additives could activate nitroalkenes when used as substrates by double hydrogen bonding, which lead to improved reactivities [24]. Based on these findings, we decided to explore the effect of a Brønsted acid or an achiral thiourea as additive on the reaction between *trans-*β-nitrostyrene and 3-phenylpropionaldehyde promoted by **OC4** (Table 4). When thioureas were used as additives the reaction was performed in toluene in order to improve the solubility.

**Table 4:** Screening of additives for the Michael addition of 3-phenylpropionaldehyde to *trans*-β-nitrostyrene using catalysts **OC4**.^a^

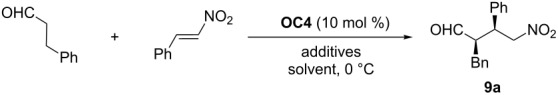						

Acid^b^	TU^c^	Solvent	Yield^d^ (%)	*syn*:*anti*^d^	ee *syn*^e^ (%)	ee *anti*^e^ (%)

none	none	methylcyclohexane	87	93:7	85	58
PhCO_2_H	none	methylcyclohexane	93	75:25	77	83
AcOH	none	methylcyclohexane	98	60:40	75	83
TFA	none	methylcyclohexane	32	76:24	83	82
none	none	toluene	84	86:4	80	39
none	**TU1**	toluene	92	76:24	72	63
none	**TU2**	toluene	87	87:13	61	24
PhCO_2_H	**TU1**	toluene	94	67:33	87	91
AcOH	**TU1**	toluene	90	80:20	80	58
PhCO_2_H	**TU2**	toluene	94	74:26	83	80
AcOH	**TU2**	toluene	85	90:10	77	36

^a^Reaction performed using 0.2 mmol of β-nitrostyrene, 0.4 mmol of 3-phenylpropionaldehyde, 10 mol % of **OC4** and 10 mol % of additive in the given solvent (2 mL) at 0 °C for 24 h. ^b^AcOH = acetic acid, TFA = trifluoroacetic acid. ^c^TU1 = *N*,*N*'-diphenylthiourea, TU2 = *N*,*N*'-bis[3,5-di(trifluoromethyl)phenyl]thiourea. ^d^Determined from the crude reaction mixture by ^1^H NMR spectroscopy using 1,3,5-trimethoxybenzene as internal standard. ^e^Determined by chiral HPLC.

The addition of benzoic or acetic acid increased the reactivity and *anti*-enantioselectivity but it was detrimental for the diastereoselectivity and *syn*-enantioselectivity. On the other hand, in the presence of trifluoroacetic acid the reaction proceeded slowly and the diastereoselectivity decreased to some extent. The presence of an achiral thiourea did not improve the results.

Finally, we considered the possibility of accelerating the formation of the enamine intermediate and simultaneously activating the nitroalkene by using a combination of organocatalyst **OC4**, a Brønsted acid and an achiral thiourea. Thus the reaction was repeated in the presence of a combination of benzoic acid and *N*,*N*'-diphenylthiourea (**TU1**) as additives. The enantioselectivity of both *syn* and *anti-*adducts reached quite good values (87% ee and 91% ee, respectively) but the diastereoselectivity dropped to 67:33.

The aryl group of benzoic acid was varied (Table 5) in an effort to improve the diastereoselectivity. The best results in terms of diastereoselectivity were obtained with the combination *p*-methoxybenzoic acid/*N*,*N*'-diphenylthiourea.

**Table 5:** Screening of benzoic acids as additives for Michael addition of 3-phenylpropionaldehyde to *trans*-β-nitrostyrene using catalysts **OC4**.^a^

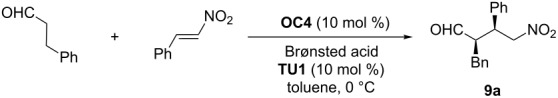			

Acid	Yield^b^ (%)	*syn*:*anti*^b^	ee *syn*^c^ (%)

PhCO_2_H	94	67:33	87
4-MeC_6_H_4_CO_2_H	89	63:37	86
4-NO_2_C_6_H_4_CO_2_H	91	61:39	90
4-FC_6_H_4_CO_2_H	90	63:37	87
4-ClC_6_H_4_CO_2_H	96	72:28	85
4-MeOC_6_H_4_CO_2_H	92	80:20	85

^a^Reaction performed using 0.2 mmol of β-nitrostyrene, 0.4 mmol of 3-phenylpropionaldehyde, 10 mol % of **OC4**, 10 mol % of *N*,*N*'-diphenylthiourea, and 10 mol % of the given benzoic acid in toluene (2 mL) at 0 °C for 24 h. ^b^Determined from the crude reaction mixture by ^1^H NMR spectroscopy using 1,3,5-trimethoxybenzene as internal standard. ^c^Determined by chiral HPLC.

Finally, with the most efficient organocatalyst **OC4** at hand we surveyed the scope of this transformation with respect to the aldehyde and the nitroolefin (Table 6). Other aliphatic aldehydes were less reactive and the reaction temperature had to be increased. Linear aliphatic aldehydes reacted with β-nitrostyrene to provide the Michael adducts in good yields when the reaction was conducted at room temperature. The diastereoselectivity was moderate to good (dr = 79:21–95:5) in favour of the *syn-*diastereoisomer and enantioselectivites ranged from 75–84% ee. The reaction of butyraldehyde with other *trans*-β-nitroolefins at room temperature also provided the Michael adducts with moderate to good distereoselectivity (dr = 74:26–92:8) in favour of the *syn-*diastereoisomer and enantioselectivites from 72–84% ee.

**Table 6:** Scope of Michael addition of aldehydes to *trans*-β-nitroolefins using catalyst **OC4**.^a^

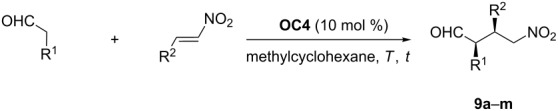							

Product	R^1^	R^2^	*T* (ºC)	*t* (h)	Yield^b^ (%)	*syn*:*anti*^b^	ee *syn*^c^ (%)

**9a**	Bn	Ph	0	24	87	93:7	85
**9b**	*n-*Pr	Ph	0	48	18	66:34	72
**9b**	*n-*Pr	Ph	rt	48	100	85:15	75
**9c**	Et	Ph	rt	22	100	77:23	82
**9d**	Me	Ph	rt	20	100	79:21	83
**9e**	*n-*Hex	Ph	rt	24	94	86:14	83
**9f**	CH_2_=CH(CH_2_)_7_	Ph	rt	70	100	95:5	84
**9g**	*n-*Pr	2-furyl	rt	24	88	74:26	72
**9h**	*n-*Pr	4-MeOC_6_H_4_	rt	44	100	87:14	80
**9i**	*n-*Pr	4-MeC_6_H_4_	rt	46	100	89:11	84
**9j**	*n-*Pr	4-ClC_6_H_4_	rt	24	100	88:12	85
**9k**	*n-*Pr	4-BrC_6_H_4_	rt	22	92	89:11	82
**9l**	*n-*Pr	3-BrC_6_H_4_	rt	31	100	93:7	84
**9m**	*n-*Pr	2-BrC_6_H_4_	rt	33	100	92:8	82

^a^Reaction performed using 0.2 mmol of nitroolefin, 0.4 mmol of aldehyde, 10 mol % of **OC4**, in methylcyclohexane (2 mL). ^b^Determined from the crude reaction mixture by ^1^H NMR spectroscopy using 1,3,5-trimethoxybenzene as internal standard. ^c^Determined by chiral HPLC.

## Conclusion

In conclusion, we have prepared 2-substituted pyrrolidines using a hydrozirconation/iodination reaction of chiral homoallylic amines. The latter were obtained on a multigram scale from imines derived from glyceraldehyde. These easily available compounds are new tuneable organocatalysts with the privileged pyrrolidine motif. When used in the asymmetric Michael addition of aldehydes to nitroolefins, diastereoselectivities of up to 93:7 and enantioselectivities of up to 85% enantiomeric excess for the *syn*-adduct were obtained in the presence of the most effective organocatalyst **OC4**.

## Supporting Information

File 1 Experimental procedures and characterization data.

File 2 NMR spectra and HPLC chromatograms.
