# Supplementary material for: Synthesis of new pyrrolidine-based organocatalysts and study of their use in the asymmetric Michael addition of aldehydes to nitroolefins
Source: Beilstein J Org Chem. 2017 Mar 27;13:612–9. doi: 10.3762/bjoc.13.59 (PMC5389197; doi:10.3762/bjoc.13.59)
Supplement: File 1 — Experimental procedures and characterization data. [file Beilstein_J_Org_Chem-13-612-s001.pdf]

# Supporting Information

# for

# Synthesis of new pyrrolidine-based organocatalysts and study of their use in the asymmetric Michael addition of aldehydes to nitroolefins

Alejandro Castán, Ramón Badorrey*, José A. Gálvez, and María D. Díaz-de-Villegas*

Address: Instituto de Síntesis Química y Catálisis Homogénea (ISQCH), CSIC - Universidad de Zaragoza, Departamento de Química Orgánica, Pedro Cerbuna 12, E-50009 Zaragoza, Spain

Email: Ramón Badorrey - badorrey@unizar.es; María D. Díaz-de-Villegas - loladiaz@unizar.es

*Corresponding author

## Experimental procedures and characterization data

## 1. General remarks

All reagents for reactions were of analytical grade and were used as obtained from commercial sources. All solvents were commercial grade and were purified prior to use according to standard methods if necessary. Dimethoxyacetals used in the synthesis of organocatalysis **OC3**–**OC10** were obtained using standard procedures as described in the literature [1-4]. Homoallylamines **1** and **3** were prepared according to our previously described procedures [5]. Whenever possible the reactions were monitored by TLC. TLC analysis was performed on precoated silica gel polyester plates with an $F_{254}$ indicator and products were visualized using ninhydrin, potassium permanganate or ethanolic phosphomolybdic acid solution followed by heating and UV light (254 nm). Column chromatography was performed using silica gel (Kiesegel 60, 230–400 mesh). Melting points were determined in open capillaries using a Gallenkamp capillary melting point apparatus and are uncorrected. FTIR spectra of oils were recorded as thin films on NaCl plates and FTIR spectra of solids were recorded as nujol dispersions on NaCl plates using a Thermo Nicolet Avatar 360 FT-IR spectrophotometer. Absorptions are expresses as $\nu_{max}$ in $cm^{-1}$ and are given for the main absorption bands. Optical rotations were measured on a Jasco 1020 polarimeter at $\lambda$ 589 nm and 25 °C in a cell with a 10 cm path length; $[\alpha]_D$ values are given in $10^{-1}$ deg·cm·$g^{-1}$ and concentrations are given in g/100 mL. $^{1}$H NMR and $^{13}$C NMR spectra were acquired on a Bruker AV-400 spectrometer operating at 400 MHz for $^{1}$H NMR, 376 MHz for $^{19}$F NMR and 100 MHz for $^{13}$C NMR at room temperature unless otherwise stated using a 5 mm probe. The chemical shifts ($\delta$) are reported in parts per million from tetramethylsilane with the solvent resonance as the internal standard [6]. Coupling constants (*J*) are quoted in Hertz. The following abbreviations are used: s, singlet; d, doublet; q, quartet; dd, doublet of doublets; m, multiplet; bs, broad singlet; ddd, doublet of doublets of

doublets. High resolution mass spectra were recorded using a Bruker Daltonics MicroToF-Q instrument from methanolic solutions using the positive electrospray ionization mode (ESI$^+$). HPLC analyses were carried out on a Waters HPLC system consisting of an M-600 low-pressure gradient pump, and an M-2996 photodiode array detector or a Waters HPLC system consisting of an M-600 low-pressure gradient pump, an M-2487 dual wavelength absorbance detector and the Waters Empower chromatography manager software. Commercially available polysaccharide chiral stationary phases Chiralcel® OD-H column, Chiralpak® IA column and Chiralpak® IC columns were used.

## 2. Synthetic procedures and analytical data

**(*R*)-2-[(*S*)-2,2-Dimethyl-1,3-dioxolan-4-yl]-1-[(*S*)-phenylethyl]pyrrolidine (2).**

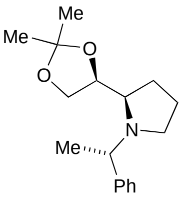


To a solution of homoallylamine **1** (1.5 g, 5.5 mmol) in $CH_2Cl_2$ (33 mL) was added $Cp_2Zr(H)Cl$ (3.1 g, 12.0 mmol) under argon at room temperature. After stirring for 1 h at room temperature, iodine (1.8 g, 7.1 mmol) was added under argon at room temperature and stirring was continued for 3 h. $CH_2Cl_2$ (50 mL) and 1 M aqueous HCl (15 mL) were added. The organic layer was separated and washed with saturated aqueous $Na_2SO_3$ (25 mL). The combined organic extracts were dried over anhydrous $MgSO_4$, filtered and concentrated in vacuo. Purification of the residue by silica gel column chromatography (eluent: $Et_2O$/hexanes, 1:3 1% v/v $Et_3N$) yielded 1.04 g (69% yield) of pyrrolidine **2** as a pale yellow oil. $[\alpha]^{25}_D$ = +34.6 (*c* = 1.00, $CHCl_3$). (KBr): $\nu$ = 1603, 1493 cm$^{-1}$. $^1$H NMR (400 MHz, $CDCl_3$): $\delta$ = 1.24 (s, 3H), 1.38 (d, *J* = 6.8 Hz, 3H), 1.39 (s, 3H), 1.61–1.69 (m, 3H), 1.71–1.75 (m, 1H), 2.51–2.58 (m, 1H), 2.71–2.77 (m, 1H), 3.06–3.11 (m, 1H), 3.68 (dd, *J* = 7.6, 6.8 Hz, 1H), 3.80–3.90 (m, 2H), 4.01 (q, *J* = 6.8 Hz, 1H), 7.28–7.38 (m, 5H). $^{13}$C NMR (100 MHz, $CDCl_3$): $\delta$ = 15.6, 24.2, 25.0, 26.4, 26.7, 49.1, 59.9,

62.6, 66.3, 78.0, 108.9, 126.8, 127.6, 128.2, 145.5; ($ESI^+$): calcd. for $C_{17}H_{26}NO_2$ $[MH]^+$ 276.1958; found 276.1978.

**(*R*)-2-[(S)-2,2-Dimethyl-1,3-dioxolan-4-yl]pyrrolidine (OC1)**

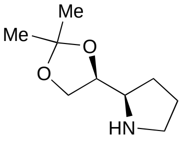


In a similar manner as described in [5] a solution of pyrrolidine **2** (284 mg, 1.03 mmol) in EtOH (20 mL) was hydrogenated with molecular hydrogen for 24 h at atmospheric pressure and room temperature in the presence of 20% $Pd(OH)_2/C$ (100 mg) as a catalyst. Afterwards, the catalyst was removed by filtration through a Celite® pad and the solvent was evaporated in vacuo. $CH_2Cl_2$ (20 mL) and 2 M aqueous NaOH (10 mL) were added. The aqueous layer was separated and extracted with $CH_2Cl_2$ (2 × 15 mL). The combined organic extracts were dried over anhydrous $MgSO_4$, filtered and concentrated in vacuo. Purification of the residue by silica gel column chromatography (eluent: EtOAc/EtOH, 1:1 1% v/v $Et_3N$) yielded 128 mg (73% yield) of organocatalyst **OC1** as a pale yellow oil. $[\alpha]^{25}_D = +7.4$ ($c$ = 1.00, $CHCl_3$). IR (KBr): $\nu$ = 3345 $cm^{-1}$. $^1H$ NMR (400 MHz, $CDCl_3$): $\delta$ = 1.27 (s, 3H), 1.34 (s, 3H), 1.20–1.40 (m, 1H), 1.76–1.77 (m, 3H), 2.81–2.87 (m, 1H), 2.93–2.98 (m, 1H), 3.05 (ddd, $J$ = 7.2, 7.2, 7.2 Hz, 1H), 3.13 (bs, 1H), 3.55–3.62 (m, 1H), 3.91–3.96 (m, 2H). $^{13}C$ NMR (100 MHz, $CDCl_3$): $\delta$ = 25.1, 25.4, 26.8, 27.4, 46.2, 61.0, 67.1, 109.3. HRMS ($ESI^+$): calcd. for $C_{10}H_{18}NO_2$ $[MH]^+$ 172.1332; found 172.1368.

**(*S*)-1-Benzyl-2-[(*S*)-2,2-dimethyl-1,3-dioxolan-4-yl]pyrrolidine (4)**

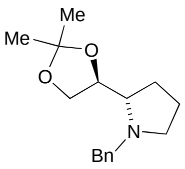


To a solution of homoallylamine **3** (614 mg, 2.35 mmol) in $CH_2Cl_2$ (33 mL) was added $Cp_2Zr(H)Cl$ (1.35 g, 5.2 mmol) under argon at room temperature. After stirring for 1 h at room temperature, iodine (776 mg, 3.06 mmol) was added under argon at room temperature and stirring was continued for 3 h. $CH_2Cl_2$ (50 mL) and 1 M aqueous HCl (15 mL) were added. The organic layer was separated and washed with saturated aqueous $Na_2SO_3$ (25 mL).

The combined organic extracts were dried over anhydrous $MgSO_4$, filtered and concentrated in vacuo. Purification of the residue by silica gel column chromatography (eluent: $Et_2O$/hexanes, 1:3 1% v/v $Et_3N$) yielded 437 mg (71% yield) of pyrrolidine **4** as a pale yellow oil. $[\alpha]^{25}_{D}$ = –34.2 (*c* = 1.00, $CHCl_3$). IR (KBr): ν = 1639, 1062 $cm^{-1}$. $^1$H NMR (400 MHz, $CDCl_3$): δ = 1.35 (s, 3H), 1.42 (s, 3H), 1.67–1.80 (m, 3H), 1.87–1.96 (m, 1H), 2.26–2.32 (m, 1H), 2.84 (ddd, *J* = 8.8, 4.4, 4.4 Hz, 1H), 2.94–2.99 (m, 1H), 3.48 (d, *J* = 12.8 Hz, 1H), 3.81 (dd, *J* = 7.6, 7.6 Hz, 1H), 4.04 (dd, *J* = 7.6, 7.4 Hz, 1H), 4.11 (d, *J* = 12.8 Hz, 1H), 4.15–4.18 (m, 1H), 7.23–7.37 (m, 5H). $^{13}$C NMR (100 MHz, $CDCl_3$): δ = 23.5, 25.4, 26.6, 27.1, 55.0, 60.4, 64.3, 66.9, 78.1, 108.8, 126.9, 128.3, 128.9, 139.8. HRMS (ESI$^+$): calcd. for $C_{16}H_{24}NO_2$ $[MH]^+$ 262.1802; found 262.1800.

**(*S*)-2-[(S)-2,2-Dimethyl-1,3-dioxolan-4-yl]pyrrolidine (OC2)**

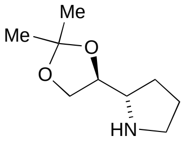


In a similar manner as described in [5] a solution of pyrrolidine **4** (248 mg, 0.95 mmol) in EtOH (20 mL) was hydrogenated with molecular hydrogen for 24 h at atmospheric pressure and room temperature in the presence of 20% $Pd(OH)_2$/C (87 mg) as a catalyst. Afterwards, the catalyst was removed by filtration through a Celite® pad and the solvent was evaporated in vacuo. $CH_2Cl_2$ (20 mL) and 2 M aqueous NaOH (10 mL) were added. The aqueous layer was separated and extracted with $CH_2Cl_2$ (2 × 15 mL). The combined organic extracts were dried over anhydrous $MgSO_4$, filtered and concentrated in vacuo. Purification of the residue by silica gel column chromatography (eluent: EtOAc/EtOH, 1:1 1% v/v $Et_3N$) yielded 123 mg (75% yield) of organocatalyst **OC2** as a pale yellow oil. $[\alpha]^{25}_{D}$ = +9.8 (*c* = 1.00, $CHCl_3$). IR (KBr): ν = 3367 $cm^{-1}$. $^1$H NMR (400 MHz, $CDCl_3$): δ = 1.32 (s, 3H), 1.39 (s, 3H), 1.53–1.64 (m, 1H), 1.64–1.80 (m, 2H), 1.80–1.92 (m, 1H), 2.33 (bs, 1H), 2.80–2.96 (m, 2H), 3.13 (ddd, *J* = 6.8, 6.8, 6.8 Hz, 1H), 3.72 (dd, *J* = 7.6, 6.0 Hz, 1H), 3.95–4.01 (m, 1H),

4.01–4.08 (m, 1H). $^{13}C$ NMR (100 MHz, $CDCl_3$): $\delta$ = 25.4, 25.8, 26.8, 28.1, 47.1, 60.7, 67.7, 78.9, 109.1. HRMS (ESI$^+$): calcd. for $C_{10}H_{18}NO_2$ $[MH]^+$ 172.1332; found 172.1340.

**(*R*)-1-Benzyloxycarbonyl-2-[(S)-1,2-dihydroxyethyl]pyrrolidine (5)**

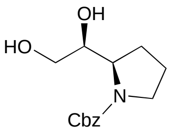


A solution of pyrrolidine **2** (450 mg, 1.63 mmol) and TFA (375 μL, 4.90 mmol) in EtOH (30 mL) was hydrogenated with molecular hydrogen for 6 h at atmospheric pressure and room temperature in the presence of 20% $Pd(OH)_2/C$ (112 mg) as a catalyst. The catalyst was removed by filtration through a Celite® pad. Saturated aqueous $NaHCO_3$ was added (10 mL) and EtOH was removed in vacuo. The residue was diluted with water (10 mL), $CH_2Cl_2$ (20 mL) and 2 M aqueous NaOH (5 mL). The organic layer was collected and the aqueous layer was extracted with $CH_2Cl_2$ (2 × 15 mL). The combined organic extracts were dried over anhydrous $MgSO_4$, filtered and concentrated in vacuo to afford an oily compound, which was used in the next step without additional purification. The resulting compound was dissolved in anhydrous $CH_2Cl_2$ (27 mL) and DIPEA (850 μL, 4.90 mmol) and benzyl chloroformate (350 μL, 2.45 mmol) were added sequentially. After stirring for 12 h at room temperature water was added (10 mL), the organic layer was collected and the aqueous layer was extracted with $CH_2Cl_2$ (2 × 15 mL). The combined organic extracts were dried over anhydrous $MgSO_4$, filtered and concentrated in vacuo to afford an oily compound, which was used in the next step without additional purification. The resulting compound was dissolved in MeOH/water 3:1 (12 mL) and TFA (50 μL, 0.65 mmol) was added. After stirring for 12 h at room temperature saturated aqueous $NaHCO_3$ was added (5 mL) and EtOH was removed in vacuo. The residue was diluted with water (5 mL) and $CH_2Cl_2$ (20 mL). The organic layer was collected and the aqueous layer was extracted with $CH_2Cl_2$ (2 × 15 mL). The combined organic extracts were dried over anhydrous $MgSO_4$, filtered and

concentrated in vacuo. Purification of the residue by silica gel column chromatography (first eluent: $Et_2O$/hexanes, 2:1; second eluent EtOAc) yielded 254 mg (59% yield) of compound **5** as a pale yellow oil. $[\alpha]^{25}_{D}$ = +77.7 (*c* = 1.00, $CHCl_3$). IR (KBr): ν = 3404, 1674 $cm^{-1}$. $^{1}$H NMR (400 MHz, $CDCl_3$): δ = 1.70–1.85 (m, 2H), 1.88–1.98 (m, 1H), 1.99–2.06 (m, 1H), 3.36 (ddd, *J* = 10.8, 7.3, 6.0 Hz, 1H), 3.51–3.56 (m, 1H), 3.64–3.68 (m, 3H), 4.02–4.08 (m, 1H), 5.14 (s, 2H), 7.31–7.36 (m, 5H). $^{13}$C NMR (100 MHz, $CDCl_3$): δ = 24.3, 28.7, 47.5, 60.3, 64.4, 67.7, 75.9, 128.0, 128.3, 128.6, 136.3, 158.2. HRMS (ESI$^+$): calcd. for $C_{14}H_{19}NO_4Na$ $[MNa]^+$ 288.1206; found 288.1196.

**(*R*)-1-Benzyloxycarbonyl-2-[(*S*)-2,2-diphenyl-1,3-dioxolan-4-yl]pyrrolidine (6)**

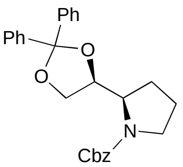


$SnCl_2$ (8.7 mg, 0.046 mmol) was added to a solution of compound **5** (242 mg, 0.91 mmol) and diphenyldimethoxymethane (416 mg, 1.82 mmol) in DME (7 mL) and the resulting mixture was stirred and heated under reflux conditions for 3 h. The reaction was quenched with pyridine (8 μL) and concentrated in vacuo. Purification of the residue by silica gel column chromatography (first eluent: $Et_2O$/hexanes, 1:4; second eluent $Et_2O$/hexanes, 1:1) yielded 355 mg (91% yield) of compound **6** as a pale yellow oil. $[\alpha]^{25}_{D}$ = +84.2 (*c* = 1.00, $CHCl_3$). IR (KBr): ν = 1699 $cm^{-1}$. $^{1}$H NMR (400 MHz, $CDCl_3$, 333 K): δ = 1.82–1.89 (m, 1H), 2.02–2.14 (m, 3H), 3.33–3.36 (m, 1H), 3.64–3.65 (m, 1H), 3.97–4.01 (m, 1H), 4.11 (bs, 1H), 4.31–4.35 (m, 1H), 4.53–4.54 (m, 1H), 5.19 (s, 2H), 7.28–7.40 (m, 11H), 7.61–7.65 (m, 4H). $^{13}$C NMR (100 MHz, $CDCl_3$, 333 K): δ = 24.1, 28.1, 47.5, 58.0, 66.6, 67.0, 78.5, 109.8, 126.1, 126.3, 127.8, 127.9, 128.1, 128.5, 137.0, 142.5, 142.8, 155.6. HRMS (ESI$^+$): calcd. for $C_{27}H_{27}NO_4Na$ $[MNa]^+$ 452.1832; found 452.1869.

**(*R*)-2-[(S)-2,2-Diphenyl-1,3-dioxolan-4-yl]pyrrolidine (OC3)**

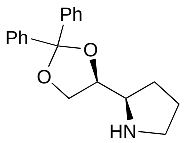

In a similar manner as described in [5] a solution of pyrrolidine **6** (226 mg, 0,53 mmol) in EtOH (10 mL) was hydrogenated with molecular hydrogen for 12 h at atmospheric pressure and room temperature in the presence of 10% Pd/C (18 mg) as a catalyst. Afterwards the catalyst was removed by filtration through a Celite® pad and the solvent evaporated in vacuo. $CH_2Cl_2$ (20 mL) and 2 M aqueous NaOH (10 mL) were added. The aqueous layer was separated and extracted with $CH_2Cl_2$ (2 × 10 mL). The combined organic extracts were dried over anhydrous $MgSO_4$, filtered and concentrated in vacuo. Purification of the residue by silica gel column chromatography (eluent: EtOAc/EtOH, 1:1 1% v/v $Et_3N$) yielded 142 mg (92% yield) of organocatalyst **OC3** as a pale yellow oil. $[\alpha]^{25}_D = +16.7$ ($c$ = 1.00, $CHCl_3$). IR (KBr): $\nu$ = 3351 $cm^{-1}$. $^1H$ NMR (400 MHz, $CDCl_3$): $\delta$ = 1.31–1.39 (m, 1H), 1.69–1.81 (m, 3H), 2.25 (bs, 1H), 2.89–2.93 (m, 1H), 2.97–3.01 (m, 1H), 3.13–3.20 (m, 1H), 3.79–3.85 (m, 1H), 4.01–4.09 (m, 2H), 7.24–7.25 (m, 6H), 7.49–7.55 (m, 4H). $^{13}C$ NMR (100 MHz, $CDCl_3$): $\delta$ = 25.2, 27.4, 46.4, 67.0, 67.7, 80.2, 109.9, 126.2, 126.3, 128.0, 128.1, 128.1, 128.2, 142.4, 142.5. HRMS ($ESI^+$): calcd. for $C_{19}H_{22}NO_2$ $[MH]^+$ 296.1645; found 296.1643.

**(*S*)-1-Benzyloxycarbonyl-2-[(*S*)-1,2-dihydroxyethyl]pyrrolidine (7)**

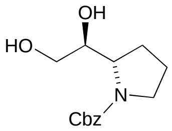

A solution of pyrrolidine **4** (700 mg, 2.68 mmol) and TFA (615 μL, 8.04 mmol) in EtOH (53 mL) was hydrogenated with molecular hydrogen for 6 h at atmospheric pressure and room temperature in the presence of 20% $Pd(OH)_2/C$ (175 mg) as a catalyst. Afterwards the catalyst was removed by filtration through a Celite® pad. Saturated aqueous $NaHCO_3$ was added (10 mL) and EtOH was removed in vacuo. The residue was diluted with water (10 mL), $CH_2Cl_2$ (20 mL) and 2 M aqueous NaOH (5 mL). The organic layer was collected and the aqueous layer was extracted with $CH_2Cl_2$ (2 × 15 mL). The combined organic

extracts were dried over anhydrous $MgSO_4$, filtered and concentrated in vacuo to afford an oily compound, which was used in the next step without additional purification. The resulting compound was dissolved in anhydrous $CH_2Cl_2$ (27 mL), and DIPEA (1,40 mL, 8.04 mmol) and benzyl chloroformate (575 μL, 4.02 mmol) were added sequentially. After stirring for 12 h at room temperature water was added (10 mL), the organic layer was collected and the aqueous layer was extracted with $CH_2Cl_2$ (2 × 15 mL). The combined organic extracts were dried over anhydrous $MgSO_4$, filtered and concentrated in vacuo to afford an oily compound, which was used in the next step without additional purification. The resulting compound was dissolved in MeOH/water 3:1 (22 mL) and TFA (50 μL, 0.67 mmol) was added. After stirring for 12 h at room temperature saturated aqueous $NaHCO_3$ (5 mL) was added and EtOH was removed in vacuo. The residue was diluted with water (5 mL) and $CH_2Cl_2$ (20 mL). The organic layer was collected and the aqueous layer was extracted with $CH_2Cl_2$ (2 × 15 mL). The combined organic extracts were dried over anhydrous $MgSO_4$, filtered and concentrated in vacuo. Purification of the residue by silica gel column chromatography (first eluent: $Et_2O$/hexanes, 2:1; second eluent EtOAc) yielded 533 mg (75% yield) of compound **7** as a white solid. Mp: 73–74 °C; $[\alpha]^{25}_D$ = –28.0 (*c* = 1.00, $CHCl_3$). IR (KBr): ν = 3406, 3350 $cm^{-1}$. $^1H$ NMR (400 MHz, $CDCl_3$): δ = 1.85–1.92 (m, 3H), 2.09–2.12 (m, 1H), 3.36–3.50 (m, 4H), 3.62 (bs, 2H), 3.90–3.95 (m, 1H), 3.96–4.10 (m, 1H), 5.14 (d, *J* = 14.4 Hz, 1H), 5.17 (d, *J* = 14.4 Hz, 1H), 7.31–7.38 (m, 5H). $^{13}C$ NMR (100 MHz, $CDCl_3$): δ = 23.4, 27.7, 47.1, 59.4, 62.9, 67.4, 72.7, 127.9, 128.2, 128.6, 136.4, 155.1. HRMS ($ESI^+$): calcd. for $C_{14}H_{19}NO_4Na$ $[MNa]^+$ 288.1206; found 288.1210.

**General procedure for the synthesis of organocatalysts OC4–OC10**

$SnCl_2$ (9.5 mg, 0.05 mmol) was added to a solution of compound **7** (265 mg, 1.00 mmol) and the corresponding dimethoxyacetal (2 mmol) in DME (7 mL) and the

resulting mixture was stirred and heated under reflux until complete disappearance of **7** (monitored by TLC). The reaction was quenched with pyridine (8 μL), concentrated in vacuo and the residue was purified by silica gel column chromatography (first eluent: $Et_2O$/hexanes, 1:4; second eluent $Et_2O$/hexanes, 1:1). The resulting compound was dissolved in EtOH (18 mL per mmol of substrate) and hydrogenated with molecular hydrogen for 12 h at atmospheric pressure and room temperature in the presence of 10% Pd/C (8 mg per 100 mg of substrate) as a catalyst. The catalyst was removed by filtration through a Celite® pad and the solvent was evaporated in vacuo. $CH_2Cl_2$ (20 mL) and 2 M aqueous NaOH (10 mL) were added. The aqueous layer was separated and extracted with $CH_2Cl_2$ (2 × 10 mL). The combined organic extracts were dried over anhydrous $MgSO_4$, filtered and concentrated in vacuo. Purification of the residue by silica gel column chromatography (eluent: EtOAc/EtOH, 1:1 1% v/v $Et_3N$) yielded the corresponding organocatalysts **OC4–OC10.**

**(*S*)-2-[(*S*)-2,2-Diphenyl-1,3-dioxolan-4-yl]pyrrolidine (OC4)**

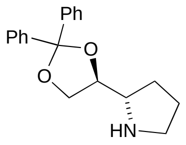


Colourless oil, 160 mg (95% yield) from 0.57 mmol of **7**. $[\alpha]^{25}_D$ = +12.1 (*c* = 1.00, $CHCl_3$). IR (KBr): ν = 3361 $cm^{-1}$. $^1H$ NMR (400 MHz, $CDCl_3$): δ = 1.70–1.85 (m, 3H), 1.92–2.01 (m, 1H), 2.32 (bs, 1H), 2.88–3.00 (m, 2H), 3.23–3.28 (m, 1H), 3.95–4.00 (m, 1H), 4.10–4.16 (m, 2H), 7.28–7.39 (m, 6H), 7.53–7.58 (m, 4H). $^{13}C$ NMR (100 MHz, $CDCl_3$): δ = 25.8, 28.4, 47.1, 60.6, 68.4, 79.6, 109.8, 126.2, 126.3, 128.0, 128.1, 128.2, 142.6, 142.6. HRMS ($ESI^+$): calcd. for $C_{19}H_{22}NO_2$ $[MH]^+$ 296.1645; found 296.1637.

**(*S*)-2-[(*S*)-2,2-Bis(4-methylphenyl)-1,3-dioxolan-4-yl]pyrrolidine (OC5)**

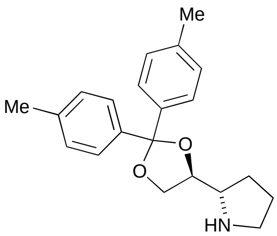


Colourless oil, 72.5 mg (86% yield) from 0.26 mmol of **7**. $^1H$ NMR (400 MHz, $CDCl_3$): δ = 1.68–1.82 (m, 3H), 1.90–1.98 (m,

1H), 2.33 (s, 3H), 2.34 (s, 3H), 2.57 (bs, 1H), 2.85–2.99 (m, 2H), 3.21–3.26 (m, 1H), 3.90–3.95 (m, 1H), 4.07–4.12 (m, 2H), 7.11–7.16 (m, 4H), 7.37–7.42 (m, 4H). $^{13}$C NMR (100 MHz, $CDCl_3$): $\delta$ = 21.2, 25.8, 28.4, 47.1, 60.7, 68.3, 79.4, 110.0, 126.2, 126.3, 128.7, 128.9, 137.6, 137.7, 139.7, 139.9.

**(*S*)-2-[(*S*)-2,2-Bis(4-methoxyphenyl)-1,3-dioxolan-4-yl]pyrrolidine (OC6)**

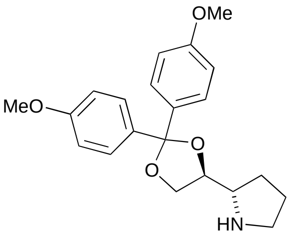


Colourless oil, 80.2 mg (87% yield) from 0.26 mmol of **7**. $^{1}$H NMR (400 MHz, $CDCl_3$): $\delta$ = 1.68–1.81 (m, 2H), 1.91–1.98 (m, 1H), 2.87–2.99 (m, 2H), 3.24 (ddd, *J* = 6.8, 6.8, 6.8 Hz, 1H), 3.76 (s, 3H), 3.78 (s, 3H), 3.87 (bs, 1H), 3.90 (dd, *J* = 7.5, 5.6 Hz, 1H), 4.04–4.13 (m, 2H), 6.81–6.86 (m, 4H), 7.36–7.42 (m, 4H). $^{13}$C NMR (100 MHz, $CDCl_3$): $\delta$ = 25.6, 28.2, 46.9, 55.3, 60.7, 68.2, 78.9, 110.0, 113.3, 113.5, 127.8, 134.6, 134.9, 159.4, 159.4.

**(*S*)-2-[(*S*)-2,2-Bis(4-fluorophenyl)-1,3-dioxolan-4-yl]pyrrolidine (OC7)**

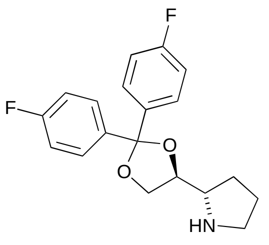


Colourless oil, 102.4 mg (81% yield) from 0.38 mmol of **7**. $^{1}$H NMR (400 MHz, $CDCl_3$): $\delta$ = 1.68–1.82 (m, 3H), 1.88–1.97 (m, 1H), 2.45 (bs, 1H), 2.85–2.95 (m, 2H), 3.18–3.23 (m, 1H), 3.89–3.95 (m, 1H), 4.043–4.09 (m, 2H), 6.95–7.03 (m, 4H), 7.41–7.49 (m, 4H). $^{13}$C NMR (100 MHz, $CDCl_3$): $\delta$ = 25.8, 28.5, 47.1, 60.5, 68.4, 79.7, 109.2, 115.0 (d, *J* = 21.4 Hz), 115.1 (d, *J* = 21.4 Hz), 128.1 (d, *J* = 8.2 Hz), 128.2 (d, *J* = 8.2 Hz), 138.2 (d, *J* = 3.0 Hz), 138.4 (d, *J* = 3.1 Hz), 162.6 (d, *J* = 245.1 Hz), 162.6 (d, *J* = 245.1 Hz); $^{19}$F NMR (376 MHz, $CDCl_3$): $\delta$ = –114.1, –114.3.

**(*S*)-2-[(*S*)-2,2-Diisopropyl)-1,3-dioxolan-4-yl]pyrrolidine (OC8)**

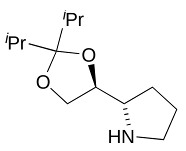


Yellowish oil, 30.9 mg (54% yield) from 0.25 mmol of **7**. $^{1}$H NMR (400 MHz, $CDCl_3$): $\delta$ = 0.89–0.95 (m, 12H), 1.64–1.82 (m, 3H), 1.94–2.08 (m, 3H), 2.84 (bs, 1H), 2.86–2.95 (m, 2H), 3.14 (ddd, *J* = 7.2, 7.2, 7.2

Hz, 1H), 3.66 (dd, $J$ = 8.4, 7.6 Hz, 1H), 3.94–3.99 (m, 1H), 4.18 (dd, $J$ = 7.2, 6.4 Hz, 1H). $^{13}$C NMR (100 MHz, $CDCl_3$): $\delta$ = 17.5, 17.6, 17.7, 17.8, 25.6, 29.6, 33.9, 34.8, 47.0, 61.2, 71.1, 81.1, 116.5.

**(*S*)-2-{(*S*)-spiro[2,9'-fluorene-(1,3)-dioxolan]-4-yl}pyrrolidine (OC9)**

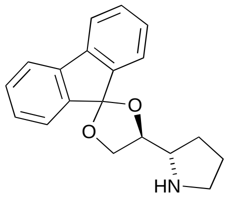


Colourless oil, 94 mg (84% yield) from 0.38 mmol of **7**. $^{1}$H NMR (400 MHz, $CDCl_3$): $\delta$ = 1.71–1.89 (m, 3H), 1.97–2.04 (m, 1H), 2.67 (bs, 1H), 2.93–3.03 (m, 2H), 3.46 (ddd, $J$ = 7.2, 7.2, 7.2 Hz, 1H), 4.20–4.26 (m, 1H), 4.52–4.59 (m, 2H), 7.25–7.32 (m, 2H), 7.34–7.41 (m, 2H), 7.48–7.56 (m, 4H). $^{13}$C NMR (100 MHz, $CDCl_3$): $\delta$ = 25.8, 28.4, 47.1, 60.6, 69.0, 80.1, 113.1, 119.9, 120.0, 123.7, 124.2, 128.3, 128.3, 130.1, 130.5, 139.3, 140.0, 143.5, 144.9.

**(*S*)-2-{(*S*)-Spiro[2,9'-xanthene-(1,3)-dioxolan]-4-yl}pyrrolidine (OC10)**

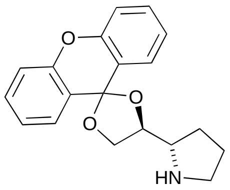


Yellowish oil, 51.1 mg (43% yield) from 0.38 mmol of **7**. $^{1}$H NMR (400 MHz, $CDCl_3$): $\delta$ = 1.74–1.92 (m, 3H), 2.03–2.12 (m, 1H), 3.02–3.05 (m, 2H), 3.50–3.55 (m, 2H), 4.17–4.23 (m, 1H), 4.50–4.57 (m, 2H), 7.20–7.28 (m, 4H), 7.39–7.44 (m, 2H), 7.63 (dd, $J$ = 8.0, 1.6 Hz, 1H), 7.76 (dd, $J$ = 7.6, 1.6 Hz, 1H). $^{13}$C NMR (100 MHz, $CDCl_3$): $\delta$ = 25.5, 28.8, 46.9, 60.2, 69.7, 80.0, 101.8, 116.8, 117.0, 123.0, 123.3, 123.4, 123.7, 126.0, 126.4, 129.9, 130.1, 151.6, 152.1.

**(*S*)-1-Benzyloxycarbonyl-2-[(*S*)-2,2,4,4-tetraisopropyl-1,3,5,2,4-trioxadisilepan-6-yl)pyrrolidine (8)**

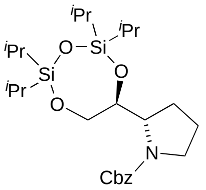


1,3-Dichloro-1,1,3,3-tetraisopropyldisiloxane (105 μL, 0.33 mmol) were added sequentially to a solution of compound **7** (59 mg, 0.22 mmol) in anhydrous DMF (1 mL) at 0 °C. Stirring was continued for 10 min at 0 °C and then for 24 h at room temperature. The reaction was

quenched with MeOH (200 μL). EtOAc (5 mL) and water (3 mL) were added. The mixture was filtered and the organic layer was collected, dried over anhydrous $MgSO_4$, filtered and concentrated in vacuo. Purification of the residue by silica gel column chromatography (eluent: $Et_2O$/hexanes, 1:6) yielded 96 mg (86% yield) of compound **8**. $^1H$ NMR (400 MHz, $CDCl_3$): (mixture of rotamers) δ = 0.80–1.12 (m, 28H), 1.70–1.83 (m, 2H), 1.96–2.08 (m, 2H), 3.30–3.39 (m, 1H), 3.48–3.69 (m, 2H), 3.74–3.91 (m, 2H), 4.24 and 4.41 (dd, *J* = 8.4, 3.3 Hz and *J* = 8.2, 3.3 Hz, 1H), 5.08 and 5.08 (d, *J* = 12.5 Hz and *J* = 12.2 Hz, 1H), 5.13 and 5.19 (d, *J* = 12.5 Hz and *J* = 12.2 Hz, 1H), 7.27–7.41 (m, 5H). $^{13}C$ NMR (100 MHz, $CDCl_3$): δ = 12.4, 12.9, 17.1, 17.2, 17.4, 17.4, 17.5, 17.6, 24.0 and 24.7, 25.2 and 26.1, 46.8 and 47.2, 59.0 and 59.5, 66.8 and 67.0, 68.3 and 68.4, 75.6 and 76.6, 128.0 and 128.0, 128.1 and 128.4, 128.5 and 128.6, 136.8 and 137.1, 154.8 and 155.0.

**(*S*)-2-[(*S*)-2,2,4,4-Tetraisopropyl-1,3,5,2,4-trioxadisilepan-6-yl)pyrrolidine (OC11)**

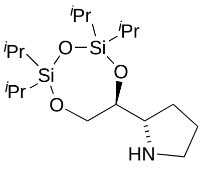


A suspension of compound **8** (96 mg, 0.19 mmol) in EtOH (3.5 mL) was hydrogenated with molecular hydrogen for 24 h at atmospheric pressure and room temperature in the presence of 10% Pd/C (7.7 mg) as a catalyst. The catalyst was removed by filtration through a Celite® pad and the solvent was evaporated in vacuo. $CH_2Cl_2$ (15 mL) and 2 M aqueous NaOH (6 mL) were added at 0 °C. The aqueous layer was separated and extracted with $CH_2Cl_2$ (2 × 10 mL). The combined organic extracts were dried over anhydrous $MgSO_4$, filtered and concentrated in vacuo. Purification of the residue by silica gel column chromatography (first eluent: $Et_2O$/hexanes, 1:1; second eluent EtOAc/EtOH, 1:1) yielded 36.6 mg (52% yield) of organocatalyst **OC11**. $^1H$ NMR (400 MHz, $CDCl_3$): δ = 0.91–1.11 (m, 28H), 1.61–1.84 (m, 4H), 2.85–2.95 (m, 1H), 3.01–3.12 (m, 2H), 3.67 (dd, *J* = 12.1, 8.2 Hz, 1H), 3.91 (dd, *J* = 12.1, 1.6 Hz, 1H), 4.10 (ddd, *J*

= 8.2, 4.9, 1.5 Hz, 1H), 4.46 (bs, 1H). $^{13}$C NMR (100 MHz, $CDCl_3$): δ = 12.5, 12.8, 13.2, 17.4, 17.4, 17.4, 17.5, 17.6, 17.6, 17.7, 25.6, 26.4, 46.6, 60.8, 68.4, 76.5.

**General procedure for the Michael reaction of aldehydes with β-nitroolefins**

A solution of the corresponding organocatalyst **OC1**–**OC11** (0.02 mmol), additive (0.02 mmol) and aldehyde (0.4 mmol) in the selected solvent (2 mL) was stirred at the specified temperature for 10 min. The nitroolefin (0.2 mmol) was added and stirring was continued for the required time at the same temperature. The reaction mixture was concentrated in vacuo. The residue was analysed by $^1$H NMR using 1,3,5-trimethoxybenzene as internal standard [7] to determine the reaction yield and diastereoselectivity. Purification of the residue by silica gel column chromatography (eluent: EtOAc/EtOH, 1:1 1% v/v $Et_3N$) yielded the corresponding Michael adducts. Enantioselectivity was determined by chiral HPLC analysis of the purified adducts using a chiral stationary phase.

**(2*R*,3*S*)-2-Benzyl-4-nitro-3-phenylbutanal (9a)**

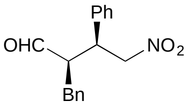


Obtained in 87% yield with a dr 93:7 and 85% ee. Chromatographic and spectroscopic data are in accordance with those previously reported [8]. Chiral HPLC analysis: Chiralcel® OD-H column, hexane/EtOH 93:7, flow rate 1.0 mL $min^{-1}$, λ = 220 nm, $t_R$ = 28.7 min (minor) and 32.1 (major). $^1$H NMR (400 MHz, $CDCl_3$): δ = 2.74–2.84 (m, 2H), 3.14 (dddd; *J* = 8.8, 8.8, 5.9, 2.4 Hz, 1H), 3.86 (ddd, *J* = 8.8, 8.8, 6.0 Hz, 1H), 4.72 (dd, *J* = 12.8, 8.8 Hz, 1H), 4.76 (dd, *J* = 12.8, 6.0 Hz, 1H), 7.04–7.07 (m, 2H), 7.23–7.37 (m, 6H), 7.37–7.43 (m, 2H), 9.73 (d, *J* = 2.2 Hz, 1H). $^{13}$C NMR (100 MHz, $CDCl_3$): δ = 34.3, 43.6, 55.4, 78.1, 127.1, 128.2, 128.4, 128.9, 128.9, 129.4, 136.8, 137.3, 203.1.

**(*R*)-2-[(*S*)-2-Nitro-1-phenylethyl]pentanal (9b)**

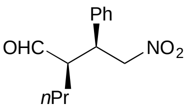


Obtained in nearly quantitative yield with a dr 85:15 and 75% ee.

Chromatographic and spectroscopic data are in accordance with those previously reported [9]. Chiral HPLC analysis: Chiralcel® OD-H column, hexane/iPrOH 95:5, flow rate 1.0 mL min$^{-1}$, λ = 220 nm, $t_R$ = 20.1 min (minor) and 23.0 (major). $^1$H NMR (400 MHz, $CDCl_3$): δ = 0.79 (t, *J* = 7.2 Hz, 3H), 1.10–1.22 (m, 1H), 1.24–1.39 (m, 2H), 1.41–1.52 (m, 1H), 2.70 (dddd, *J* = 9.2, 9.2, 3.2, 3.2 Hz, 1H), 3.77 (ddd, *J* = 9.6, 9.6, 5.2 Hz, 1H), 4.64 (dd, *J* =12.6, 9.4 Hz, 1H), 4.70 (dd, *J* =12.6, 5.2 Hz, 1H), 7.15–7.18 (m, 2H), 7.25–7.36 (m, 3H), 9.69 (d, *J* = 2.8 Hz, 1H). $^{13}$C NMR (100 MHz, $CDCl_3$): δ = 14.0, 19.9, 29.6, 43.2, 53.9, 78.5, 128.1, 128.2, 129.2, 136.9, 203.4.

**(2*R*,3*S*)-2-Ethyl-4-nitro-3-phenylbutanal (9c)**

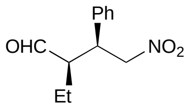


Obtained in nearly quantitative yield with a dr 77:23 and 82% ee. Chromatographic and spectroscopic data are in accordance with those previously reported [10]. Chiral HPLC analysis: Chiralpak® IC column, hexane/iPrOH 90:10, flow rate 1.0 mL min$^{-1}$, λ = 220 nm, $t_R$ = 36.6 min (minor) and 41.1 (major). $^1$H NMR (400 MHz, $CDCl_3$): δ = 0.83 (t, *J*= 7.4 Hz, 3H), 1.47–1.55 (m, 2H), 2.65–2.71 (m, 1H), 3.79 (ddd, *J* = 9.8, 9.8, 5.0 Hz, 1H), 4.63 (dd, *J* = 12.8, 9.8 Hz, 1H), 4.72 (dd, *J* = 12.8, 4.8 Hz, 1H) , 7.16–7.19 (m, 2H), 7.27–7.37 (m, 3H), 9.72 (d, *J* = 2.8 Hz, 1H). $^{13}$C NMR (100 MHz, $CDCl_3$): δ = 10.8, 20.5, 42.8, 55.1, 78.7, 128.1, 128.3, 129.2, 136.9, 203.3.

**(2*R*,3*S*)-2-Methyl-4-nitro-3-phenylbutanal (9d)**

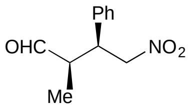


Obtained in nearly quantitative yield with a dr 79:21 and 83% ee. Chromatographic and spectroscopic data are in accordance with those previously reported [11]. Chiral HPLC analysis: Chiralcel® OD-H column, hexane/iPrOH 80:20, flow rate 1.0 mL min$^{-1}$, λ = 220 nm, $t_R$ = 18.4 min (minor) and 24.7 (major). $^1$H NMR (400 MHz, $CDCl_3$): δ = 0.99 (d, *J* = 7.2 Hz, 3H), 2.72–2.83 (m, 1H), 3.81 (ddd, *J* = 9.2, 9.2, 5.6 Hz, 1H), 4.68 (dd, *J* = 12.4, 9.2 Hz, 1H), 4.80 (dd, *J* =

12.4, 5.6 Hz, 1H), 7.14–7.18 (m, 2H), 7.26–7.35 (m, 3H), 9.70 (d, *J* = 2.0 Hz, 1H). $^{13}$C NMR (100 MHz, $CDCl_3$): δ = 12.2, 44.1, 48.5, 78.2, 128.2, 128.2, 129.2, 136.7, 202.4.

**(*R*)-2-[(*S*)-2-Nitro-1-phenylethyl]octanal (9e)**

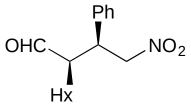


Obtained in 94% yield with a dr 86:14, and 83% ee. Chromatographic and spectroscopic data are in accordance with those previously reported [12]. Chiral HPLC analysis: Chiralcel® OD-H column, hexane/iPrOH 90:10, flow rate 0.5 mL min$^{-1}$, λ = 220 nm, $t_R$ = 29.9 min (minor) and 37.0 (major). $^{1}$H NMR (400 MHz, $CDCl_3$): δ = 0.82 (t, *J* = 7.2 Hz, 3H), 1.11–1.51 (m, 10H), 2.70 (dddd; *J* = 9.2, 9.2, 4.0, 2.8 Hz, 1H), 3.77 (ddd, *J* = 9.6, 9.6, 5.2 Hz, 1H), 4.64 (dd, *J* = 12.8, 9.6 Hz, 1H), 4.71 (dd, *J* = 12.8, 5.2 Hz, 1H), 7.16–7.19 (m, 2H), 7.26–7.38 (m, 3H), 9.70 (d, *J* = 2.8 Hz, 1H). $^{13}$C NMR (100 MHz, $CDCl_3$): δ = 14.1, 22.5, 26.5, 27.5, 29.2, 31.5, 43.3, 54.0, 78.6, 128.1, 128.3, 129.2, 136.9, 203.4.

**(*R*)-2-[(*S*)-2-Nitro-1-phenylethyl]undec-10-enal (9f)**

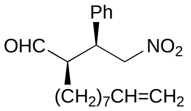


Obtained in nearly quantitative yield with a dr 95:5, and 84% ee. Chromatographic and spectroscopic data are in accordance with those previously reported [10]. Chiral HPLC analysis: Chiralpak® IC column, hexane/iPrOH 90:10, flow rate 1.0 mL min$^{-1}$, λ = 220 nm, $t_R$ = 22.7 min (minor) and 25.4 (major). $^{1}$H NMR (400 MHz, $CDCl_3$): δ = 1.11–1.52 (m, 12H), 1.96–2.02 (m, 2H), 2.69 (dddd, *J* = 9.4, 9.4, 3.8, 2.8 Hz,1H), 3.77 (ddd, *J* = 9.6, 9.6, 5.2 Hz, 1H), 4.63 (dd, *J* = 12.8, 9.4 Hz, 1H), 4.70 (dd, *J* = 12.8, 5.2 Hz, 1H), 4.92 (dddd, *J* = 10.2, 2.4, 1.2, 1.2 Hz, 1H), 4.97 (dddd, *J* = 17.0, 2.4, 1.8, 1.8 Hz, 1H), 4.78 (dddd, *J* = 17.0, 10.2, 6.8, 6.8 Hz, 1H), 7.15–7.18 (m, 2H) , 7.27–7.36 (m, 3H), 9.70 (d, *J* = 2.4, 1H). $^{13}$C NMR (100 MHz, $CDCl_3$): δ = 26.4, 27.4, 28.9, 28.9, 29.1, 29.4, 33.8, 43.2, 54.0, 78.6, 114.3, 128.1, 128.3, 129.2, 136.9, 139.2, 203.3.

**(*R*)-2-[(*R*)-1-(Furan-2-yl)-2-nitroethyl]pentanal (9g)**

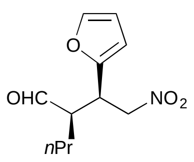

Obtained in 88% yield with a dr 74:26 and 72% ee. Chromatographic and spectroscopic data are in accordance with those previously reported [11]. Chiral HPLC analysis: Chiralcel® OD-H column, hexane/iPrOH 95:5, flow rate 0.8 mL min$^{-1}$, $\lambda$ = 220 nm, $t_R$ = 15.7 min (major) and 16.8 (minor). $^1$H NMR (400 MHz, $CDCl_3$): $\delta$ = 0.88 (t, *J* = 7.2 Hz, 3H), 1.20–1.57 (m, 4H), 2.78 (dddd, *J* = 8.2, 8.2, 4.0, 2.0 Hz, 1H), 4.00 (ddd, *J* = 9.0, 8.2, 5.2 Hz, 1H), 4.65 (dd, *J* = 12.8, 5.2 Hz, 1H), 4.72 (dd, *J* = 12.8, 9.2 Hz, 1H), 6.19 (dd, *J* = 3.2, 0.8 Hz, 1H), 6.31 (dd, *J* = 3.2, 1.8 Hz, 1H), 7.37 (dd, *J* = 1.8, 0.8 Hz, 1H), 9.71 (d, *J* = 2.0 Hz, 1H). $^{13}$C NMR (100 MHz, $CDCl_3$): $\delta$ = 14.2, 20.1, 29.2, 37.1, 52.3, 76.2, 108.9, 110.6, 142.8, 150.2, 202.5.

**(*R*)-2-[(*S*)-1-(4-Methoxyphenyl)-2-nitroethyl]pentanal (9h)**

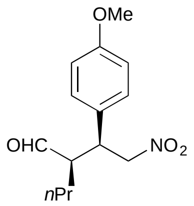

Obtained in nearly quantitative yield with a dr 87:14 and 80% ee. Chromatographic and spectroscopic data are in accordance with those previously reported [13]. Chiral HPLC analysis: Chiralpak® IC column, hexane/iPrOH 80:20, flow rate 0.8 mL min$^{-1}$, $\lambda$ = 220 nm, $t_R$ = 26.1 min (minor) and 29.2 (major). $^1$H NMR (400 MHz, $CDCl_3$): $\delta$ = 0.80 (t, *J* = 7.2 Hz, 3H), 1.11–1.22 (m, 1H), 1.26–1.40 (m, 2H), 1.42–1.52 (m, 1H), 2.65 (dddd, *J* = 9.4, 9.4, 3.2, 3.2 Hz, 1H), 3.72 (ddd, *J* = 9.8, 9.8, 5.2 Hz, 1H), 3.79 (s, 3H), 4.59 (dd, *J* =12.4, 9.6 Hz, 1H), 4.67 (dd, *J* =12.4, 5.2 Hz, 1H), 6.84–6.88 (m, 2H), 7.06–7.10 (m, 2H), 9.69 (d, *J* = 2.8 Hz, 1H). $^{13}$C NMR (100 MHz, $CDCl_3$): $\delta$ = 14.1, 19.9, 29.6, 42.6, 54.1, 55.4, 78.8, 114.6, 128.6, 129.2, 159.4, 203.5.

**(*R*)-2-[(*S*)-1-(4-Methylphenyl)-2-nitroethyl]pentanal (9i)**

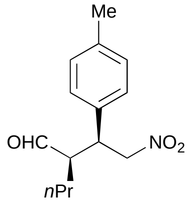

Obtained in nearly quantitative yield with a dr 89:11 and 84% ee. Chromatographic and spectroscopic data are in accordance with

those previously reported [11]. Chiral HPLC analysis: Chiralpak® IC column, hexane/iPrOH 90:10, flow rate 1.0 mL $min^{-1}$, $\lambda$ = 220 nm, $t_R$ = 27.5 min (minor) and 30.0 (major). $^1$H NMR (400 MHz, $CDCl_3$): $\delta$ = 0.80 (t, $J$ = 7.0 Hz, 3H), 1.10–1.23 (m, 1H), 1.24–1.40 (m, 2H), 1.42–1.53 (m, 1H), 2.32 (s, 3H), 2.67 (dddd, $J$ = 9.4, 9.4, 3.2, 3.2 Hz, 1H), 3.73 (ddd, $J$ = 9.6, 9.6, 5.2 Hz, 1H), 4.61 (dd, $J$ = 12.8, 9.6 Hz, 1H), 4.68 (dd, $J$ = 12.8, 5.2 Hz, 1H), 7.03–7.06 (m, 2H), 7.13–7.16 (m, 2H), 9.69 (d, $J$ = 2.8 Hz, 1H). $^{13}$C NMR (100 MHz, $CDCl_3$): $\delta$ = 14.0, 19.9, 21.2, 29.6, 43.0, 54.0, 128.0, 129.9, 133.8, 138.0, 203.5.

**(*R*)-2-[(*S*)-1-(4-Chlorophenyl)-2-nitroethyl]pentanal (9j)**

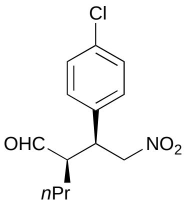


Obtained in nearly quantitative yield with a dr 88:12 and 85% ee. Chromatographic and spectroscopic data are in accordance with those previously reported [14]. Chiral HPLC analysis: Chiralpak® IC column, hexane/iPrOH 90:10, flow rate 1.0 mL $min^{-1}$, $\lambda$ = 220 nm, $t_R$ = 35.0 min (minor) and 37.6 (major). $^1$H NMR (400 MHz, $CDCl_3$): $\delta$ = 0.80 (t, $J$ = 7.2 Hz, 3H), 1.11–1.23 (m, 1H), 1.24–1.40 (m, 2H), 1.42–1.51 (m, 1H), 2.68 (dddd, $J$ = 9.2, 9.2, 3.4, 2.6 Hz, 1H), 3.76 (ddd, $J$ = 9.8, 9.8, 5.0 Hz, 1H), 4.61 (dd, $J$ = 12.8, 10.0 Hz, 1H), 4.70 (dd, $J$ = 12.8, 5.2 Hz, 1H), 7.10–7.14 (m, 2H), 7.30–7.34 (m, 2H), 9.69 (d, $J$ = 2.8 Hz, 1H). $^{13}$C NMR (100 MHz, $CDCl_3$): $\delta$ = 14.0, 19.8, 29.6, 42.6, 53.7, 78.3, 129.5, 129.5, 134.2, 135.5, 202.9.

**(*R*)-2-[(*S*)-1-(4-Bromophenyl)-2-nitroethyl]pentanal (9k)**

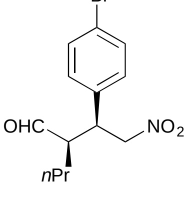


Obtained in 92% yield with a dr 89:11 and 82% ee. Chromatographic and spectroscopic data are in accordance with those previously reported [11]. Chiral HPLC analysis: Chiralcel® OD-H column, hexane/iPrOH 93:7, flow rate 1.0 mL $min^{-1}$, $\lambda$ = 220 nm, $t_R$ = 27.3 min (minor) and 29.9 (major). $^1$H NMR (400 MHz, $CDCl_3$): $\delta$ = 0.80 (t, $J$ = 7.0, 3H), 1.12–1.21 (m, 1H),

1.24–1.40 (m, 2H), 1.41–1.50 (m, 1H), 2.68 (dddd, $J$ = 9.2, 9.2, 3.6, 2.8 Hz, 1H), 3.75 (ddd, $J$ = 9.8, 9.8, 4.8 Hz, 1H), 4.60 (dd, $J$ = 12.8, 9.6 Hz, 1H), 4.69 (dd, $J$ = 12.8, 4.8 Hz, 1H), 7.04–7.08 (m, 2H), 7.46–7.49 (m, 2H), 9.68 (d, $J$ = 2.4 Hz, 1H). $^{13}$C NMR (100 MHz, $CDCl_3$): $\delta$ = 14.0, 19.8, 29.6, 42.7, 53.6, 78.2, 122.2, 129.8, 132.4, 136.0, 202.9.

**(*R*)-2-[(*S*)-1-(3-Bromophenyl)-2-nitroethyl]pentanal (9l)**

Obtained in nearly quantitative yield with a dr 93:7 and 84% ee. Chromatographic and spectroscopic data are in accordance with those previously reported [11]. Chiral HPLC analysis: Chiralpak® IC column, hexane/iPrOH 95:5, flow rate 1.0 mL min$^{-1}$, $\lambda$ = 220 nm, $t_R$ = 40.0 min (minor) and 46.4 (major). $^1$H NMR (400 MHz, $CDCl_3$): $\delta$ = 0.81 (t, $J$ = 7.2 Hz, 3H), 1.13–1.52 (m, 4H), 2.70 (dddd, $J$ = 9.2, 9.2, 3.6, 2.4 Hz, 1H), 3.74 (ddd, $J$ = 9.6, 9.6, 5.2 Hz, 1H), 4.62 (dd, $J$ = 12.8, 9.6 Hz, 1H), 4.70 (dd, $J$ = 12.8, 5.2 Hz, 1H), 7.12 (ddd, $J$ = 7.8, 1.8, 0.8 Hz, 1H), 7.22 (dd, $J$ = 7.8, 7.8 Hz, 1H), 7.33 (dd, $J$ = 1.8, 1.8 Hz, 1H), 7.44 (ddd, $J$ = 8.0, 2.0, 0.8 Hz, 1H), 9.69 (d, $J$ = 2.4 Hz, 1H). $^{13}$C NMR (100 MHz, $CDCl_3$): $\delta$ = 14.1, 19.8, 29.6, 42.8, 53.7, 78.1, 123.3, 126.8, 130.8, 131.2, 131.5, 139.5, 202.8.

**(*R*)-2-[(*S*)-1-(2-Bromophenyl)-2-nitroethyl]pentanal (9m)**

Obtained in nearly quantitative yield with a dr 92:8 and 82% ee. Spectroscopic data are in accordance with those previously reported [11]. Chiral HPLC analysis: Chiralpak® IC column, hexane/iPrOH/acetone 95:4:1, flow rate 1.0 mL min$^{-1}$, $\lambda$ = 220 nm, $t_R$ = 17.9 min (minor) and 18.9 (major). $^1$H NMR (400 MHz, $CDCl_3$): $\delta$ = 0.83 (t, $J$ = 7.0 Hz, 3H), 1.18-1.40 (m, 4H), 2.93-3.00 (m, 1H), 4.33-4.39 (m, 1H), 4.67 (dd, $J$ = 13.2, 4.8 Hz, 1H), 4.86 (dd, $J$ = 13.2, 9.2 Hz, 1H), 7.16 (ddd, $J$ = 8.0, 7.2, 1.6 Hz, 1H), 7.21 (dd, $J$ = 8.0, 1.6 Hz, 1H), 7.32 (ddd, $J$ = 8.0, 7.2, 1.2 Hz, 1H), 7.61 (dd, $J$ =

8.0, 1.2 Hz, 1H), 9.72 (d, *J* = 2.4 Hz, 1H). $^{13}C$ NMR (100 MHz, $CDCl_3$): 14.1, 20.0, 29.7, 41.9, 53.3, 76.9, 128.2, 129.0, 129.6, 131.1, 134.0, 136.4, 203.3.

## 3. References


1. Reddy, S. R., Titu, D., Chadha, A. *J. Am. Oil. Chem. Soc*. **2010**, *87*, 747–754.

2. Leonard, N. M., Oswald, M. C., Frieberg, D. A., Nattier, B. A., Smith, R. C., Mohan, R. S., *J. Org. Chem.* **2002**, *67*, 5202–5207.

3. Napolitano, E., Fiaschi, R., Mastrorilli, E. *Synthesis* **1986**, 122–125.

4. Reese, C. B., Yan, H. *J. Chem. Soc. Perkin Trans. 1* **2001**, 1807–1815.

5. Gálvez, J. A., Díaz-de-Villegas, M. D., Badorrey, R., López-Ram-de-Víu, M. P. *Org. Biomol. Chem.* **2011**, *9*, 8155–8162.

6. Residual solvent signals set according to Fulmer, G. R., Miller, A. J. M., Sherden, N. H., Gottlieb, H. E., Nudelman, A., Stolz, B. M., Bercaw, J. E., Goldberg, K. I. *Organometallics* **2010**, *29*, 2176–2179.

7. Hellriegel, C., Rück, A. *Analitix* **2012**, No 4, 8–9.

8. Ötvös, S. B., Mándity, I. M., Fülöp, F. *ChemSusChem* **2012**, *5*, 266–269.

9. Zheng, Z., Perkins, B. L., Ni, B. *J. Am. Chem. Soc.* **2010**, *132*, 50–51.

10. Sagamanova, I., Rodríguez-Escrich, C., Molnár, I. G.; Sayalero, S.; Gilmour, R.; Pericàs, M. S. *ACS Catal*. **2015**, *2015*, 6241–6248.

11. He, C., Ren, X., Feng, Y., Chai, Y., Zhang, S., Chen, W. *Tetrahedron Lett.* **2015**, *56*, 4036–4038.

12. Wang, C., Yu, C., Liu, C. L., Peng, Y. G. *Tetrahedron Lett.* **2009**, *50*, 2363–2366.

13. Ghosh, S., Qiao,Y., Ni, B., Headley, A. D.*Org. Biomol. Chem.* **2013**, *11*, 1801–1804.

14. Wang, W. H.; Abe, T.; Wang, X. B.; Kodama, K.; Hirose, T.: Zhang, G. Y. *Tetrahedron: Asymmetry* **2010**, *21*, 2925–2933.
