# Supplementary material for: Synthesis of new pyrrolidine-based organocatalysts and study of their use in the asymmetric Michael addition of aldehydes to nitroolefins
Source: Beilstein J Org Chem. 2017 Mar 27;13:612–9. doi: 10.3762/bjoc.13.59 (PMC5389197; doi:10.3762/bjoc.13.59)
Supplement: File 2 — NMR spectra and HPLC chromatograms. [file Beilstein_J_Org_Chem-13-612-s002.pdf]

Supporting Information

for

Synthesis of new pyrrolidine-based organocatalysts and study of their use in the asymmetric Michael addition of aldehydes to nitroolefins

Alejandro Castán, Ramón Badorrey*, José A. Gálvez, and María D. Díaz-de-Villegas*

Address: Instituto de Síntesis Química y Catálisis Homogénea (ISQCH), CSIC - Universidad de Zaragoza, Departamento de Química Orgánica, Pedro Cerbuna 12, E-50009 Zaragoza, Spain

Email: Ramón Badorrey - badorrey@unizar.es; María D. Díaz-de-Villegas - loladiaz@unizar.es

*Corresponding author

NMR spectra and HPLC chromatograms

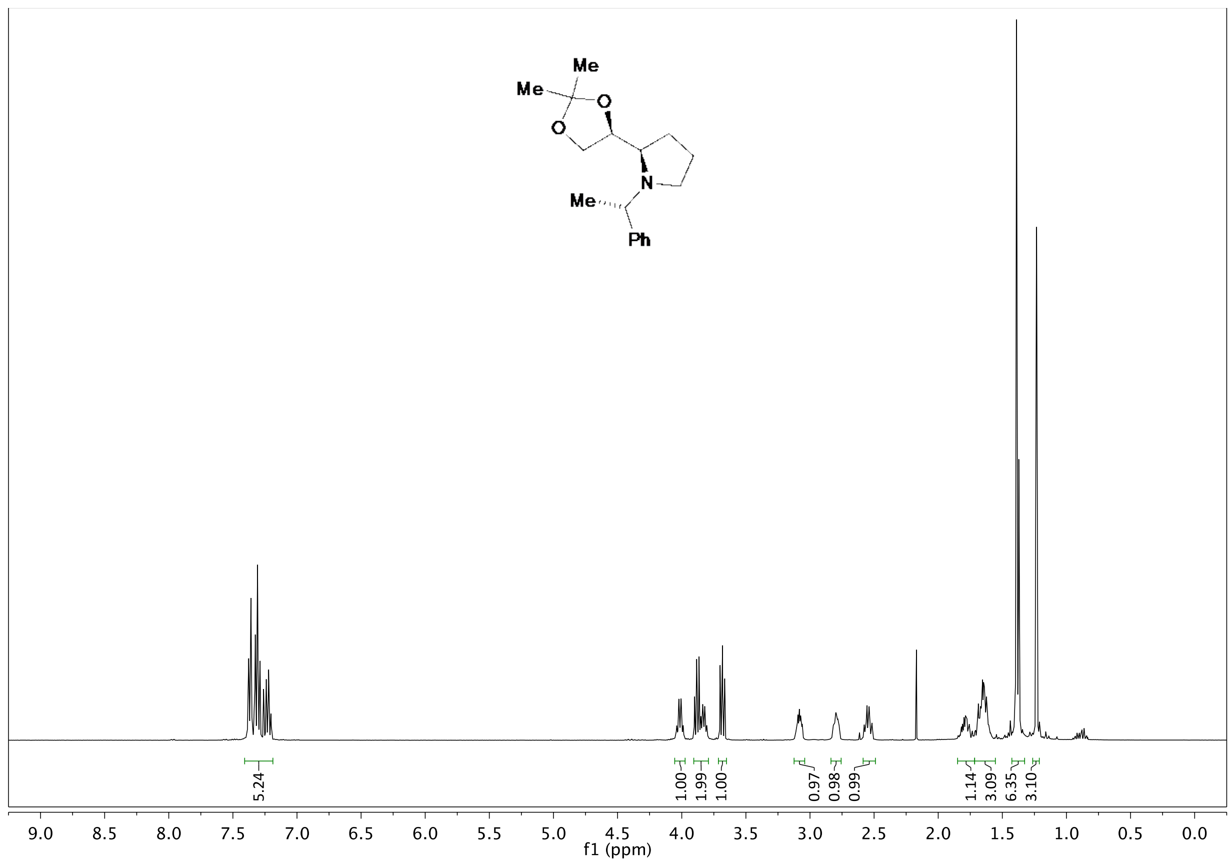

^{1}H NMR of **2**

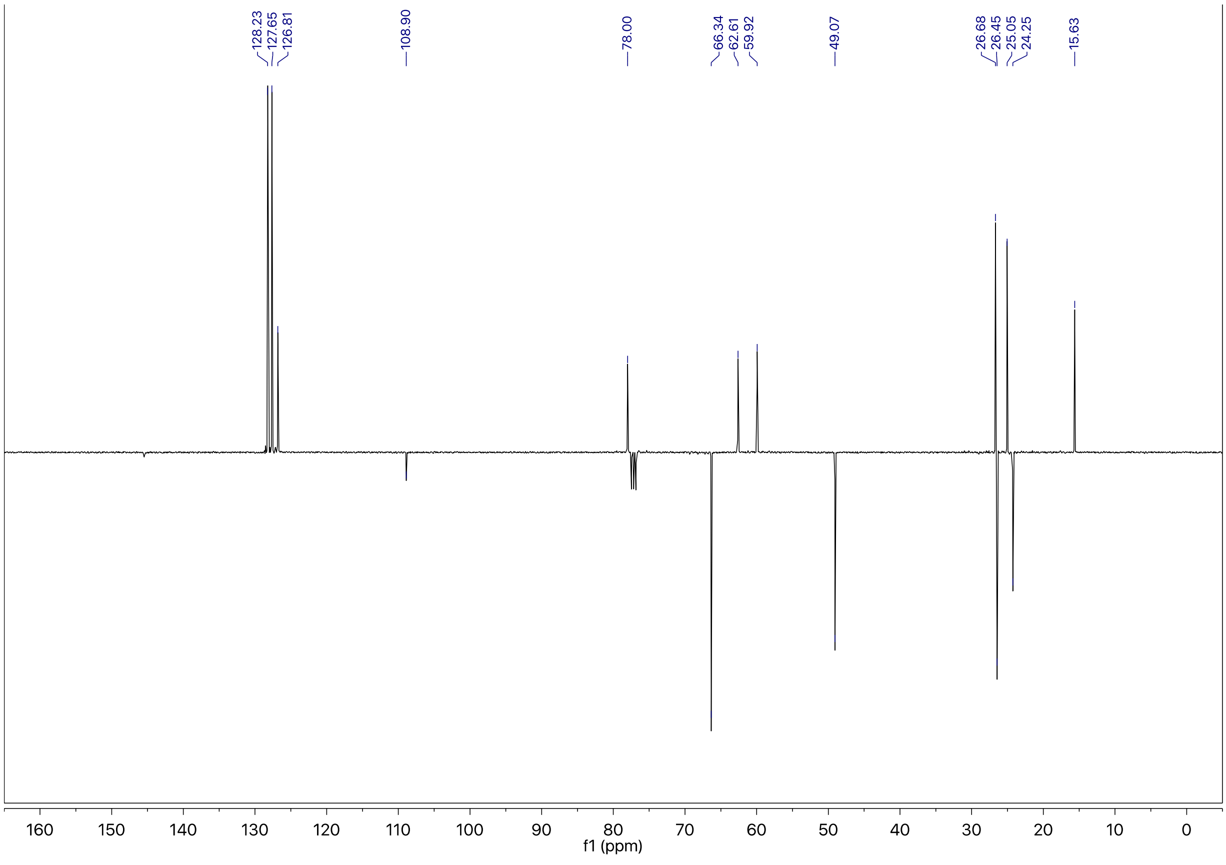

^{13}C NMR of **2**

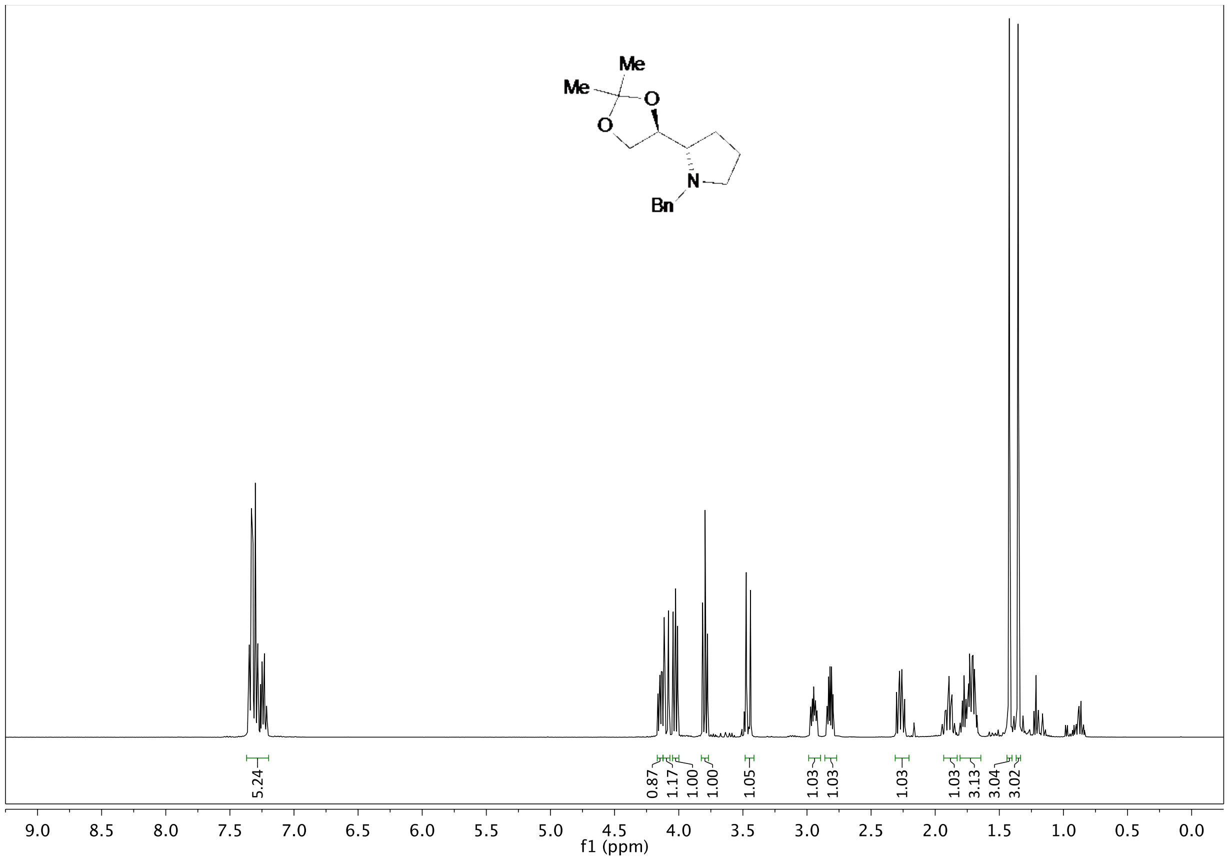

^{1}H NMR of **4**

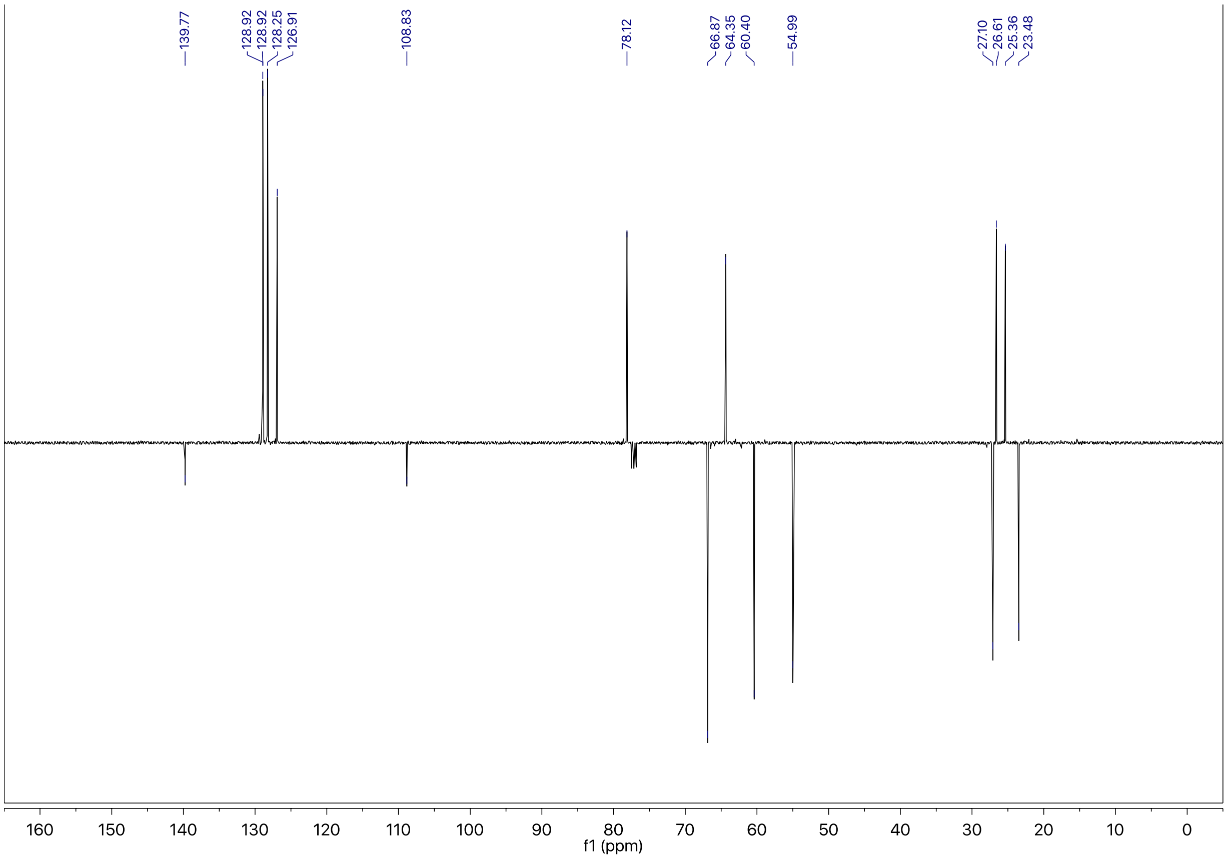

^{13}C NMR of **4**

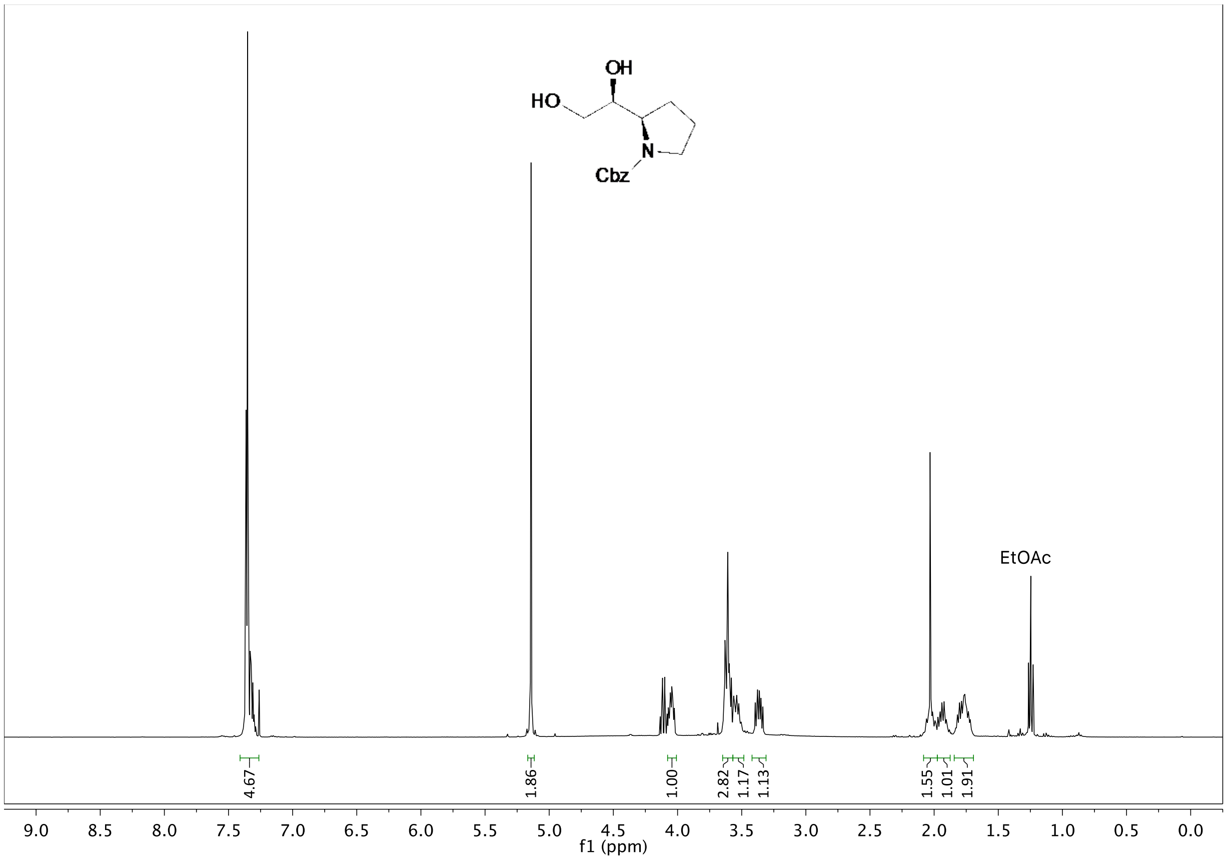

^{1}H NMR of **5**

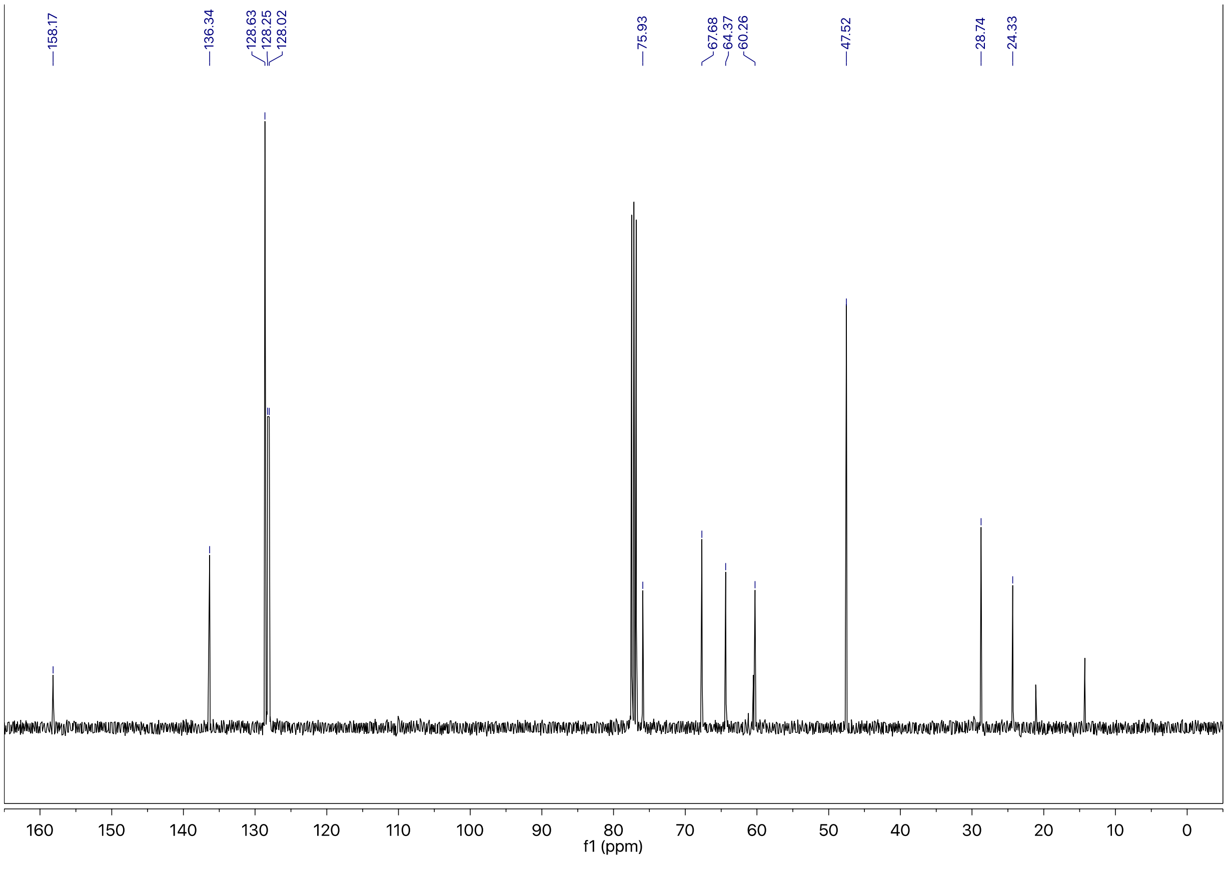

^{13}C NMR of **5**

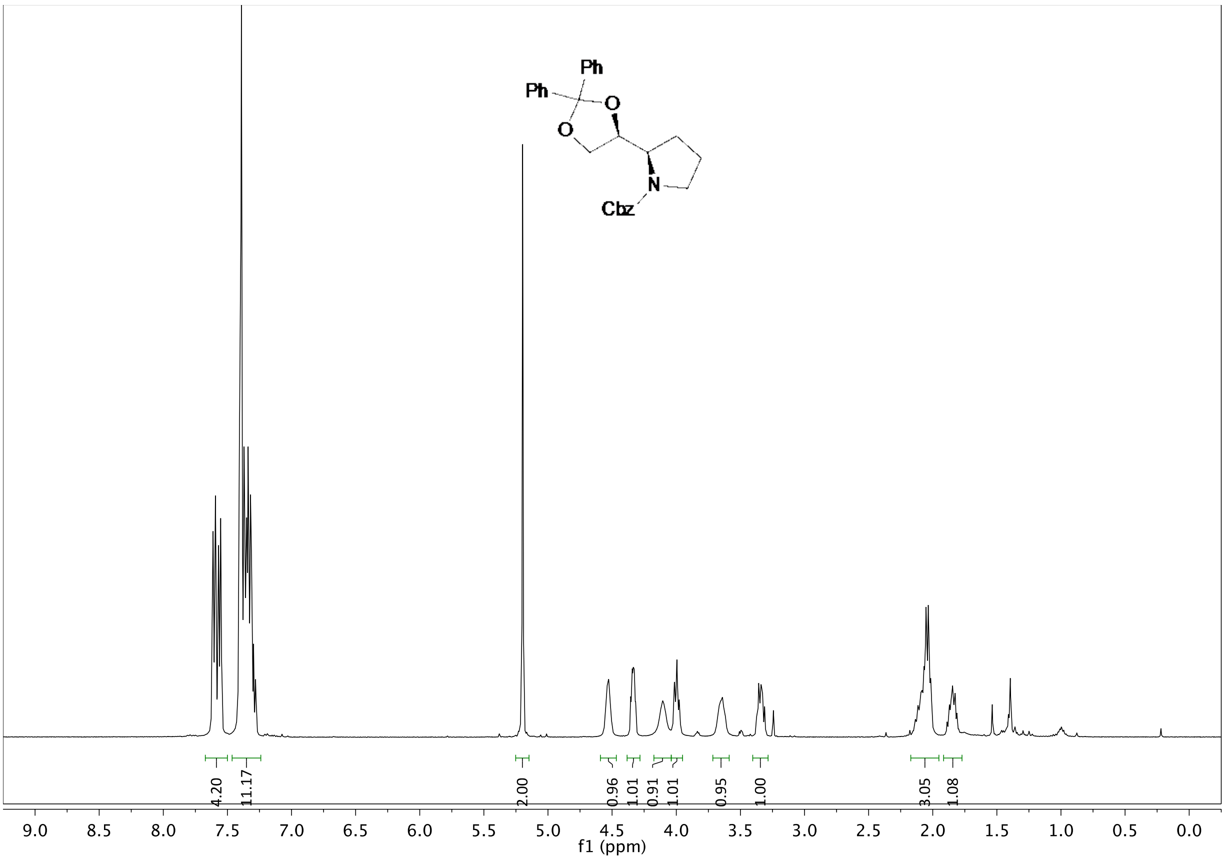

^{1}H NMR of **6**

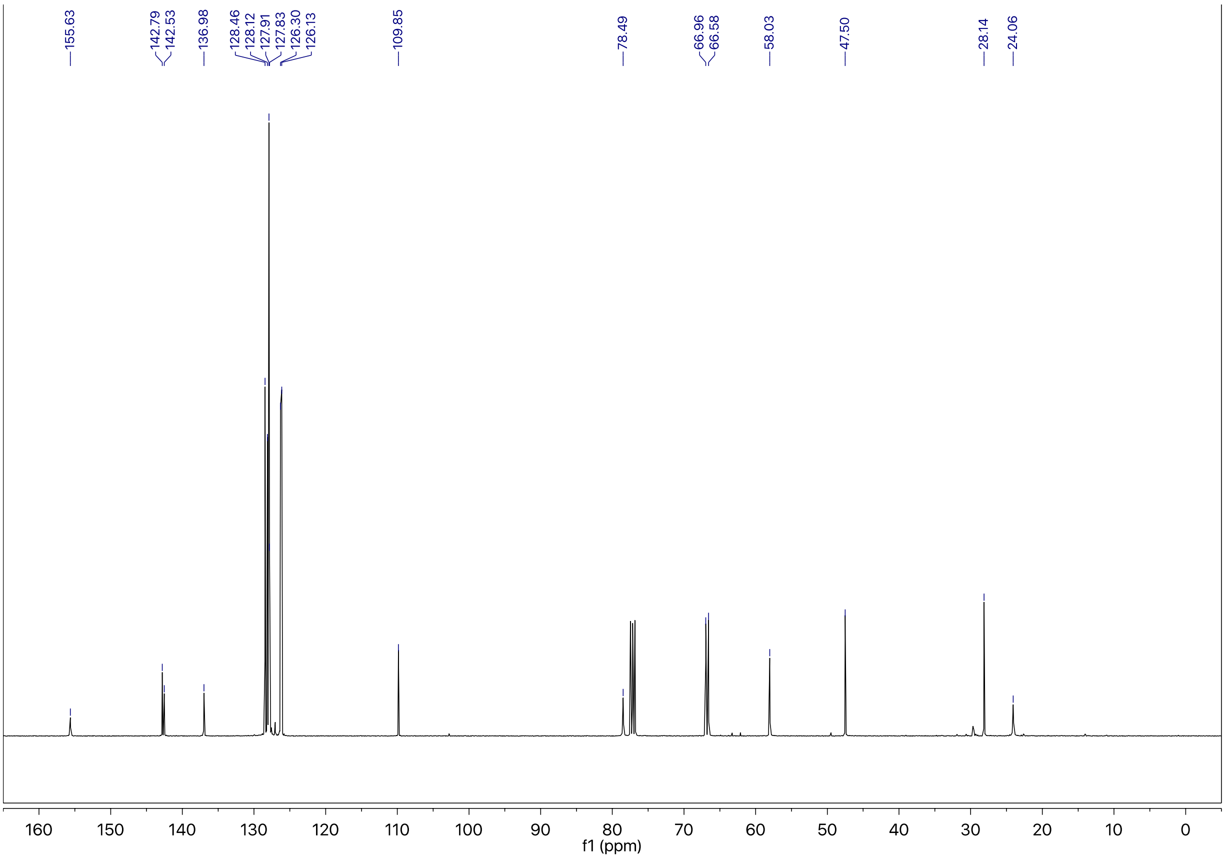

^{13}C NMR of **6**

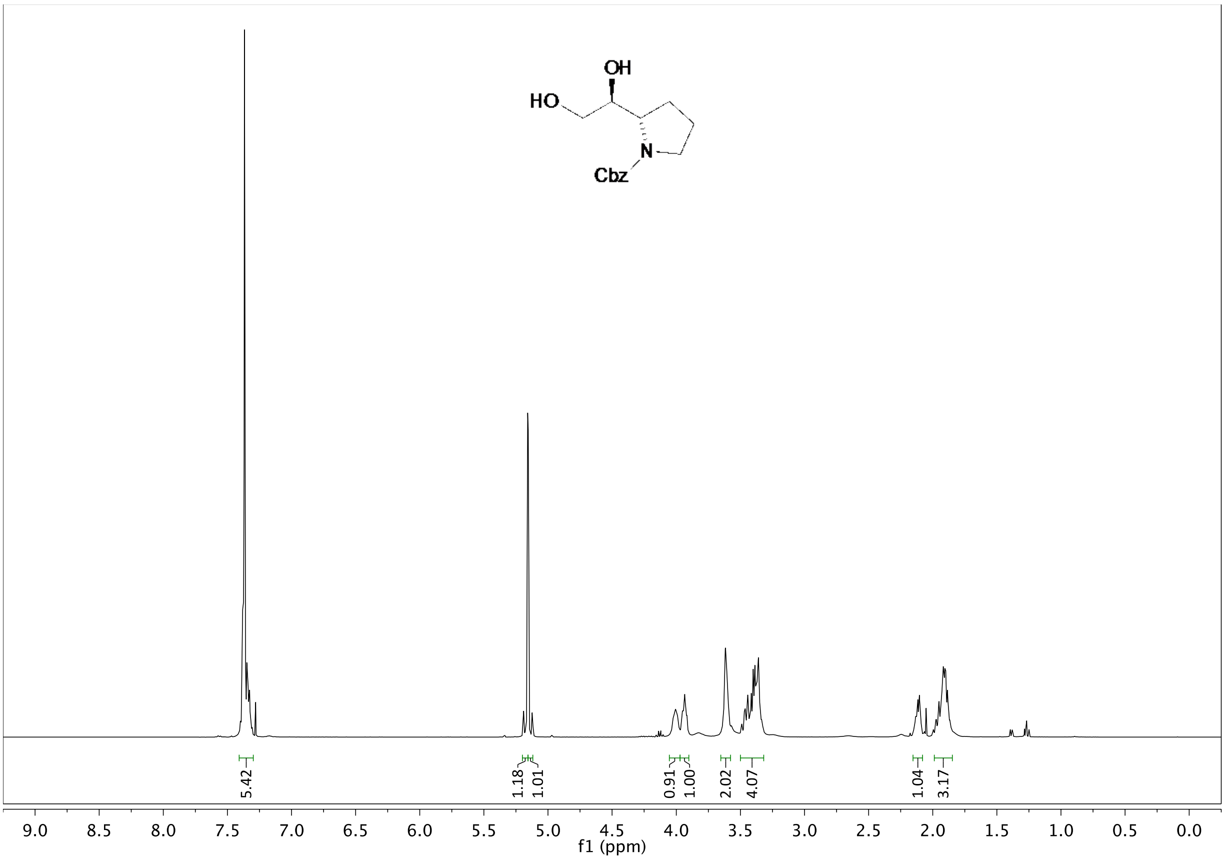

^{1}H NMR of **7**

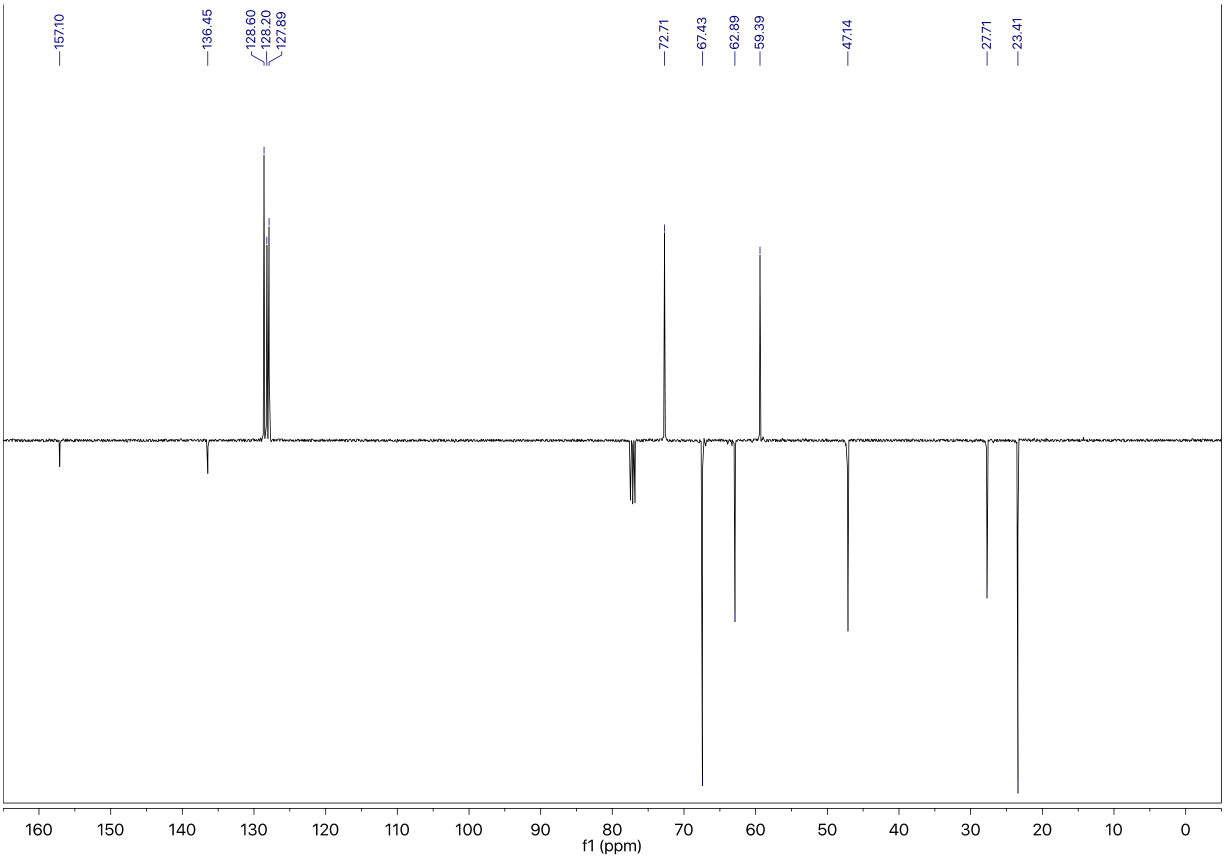

^{13}C NMR of **7**

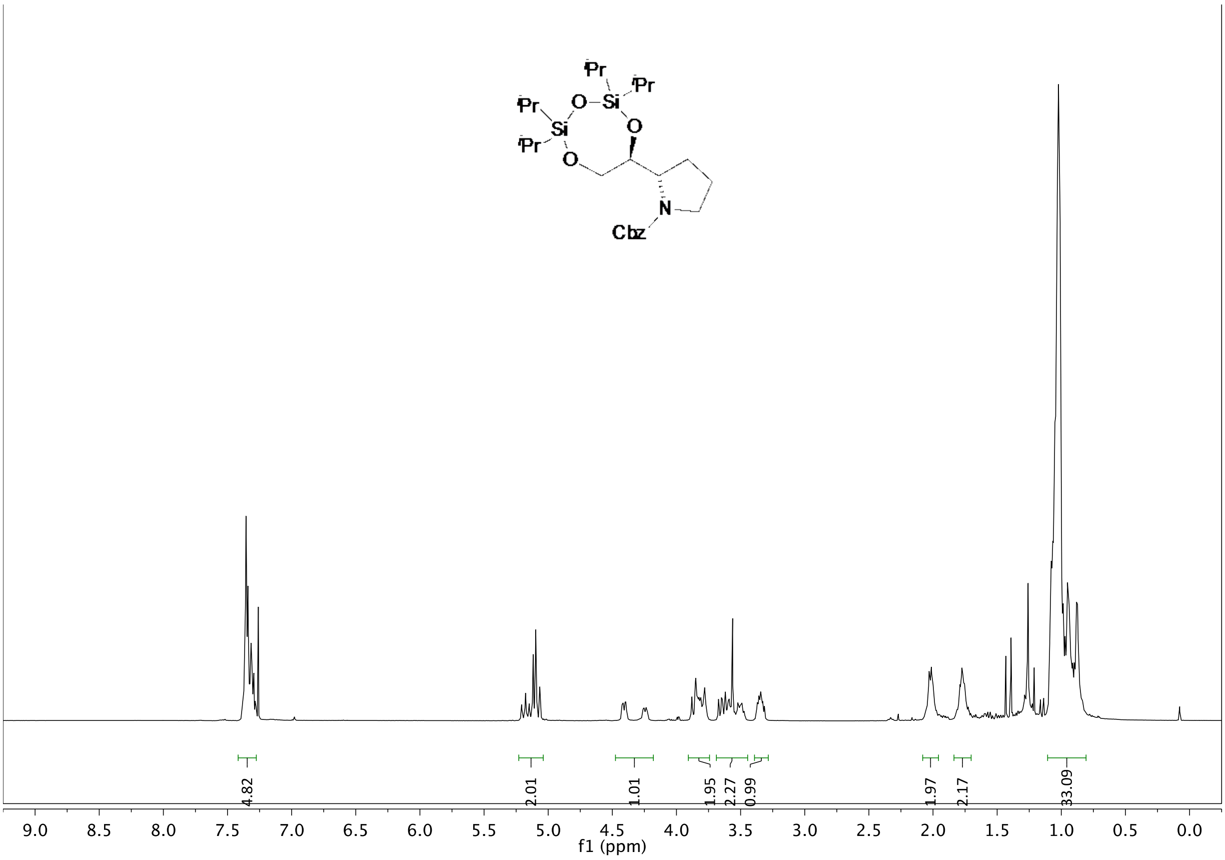

^{1}H NMR of **8**

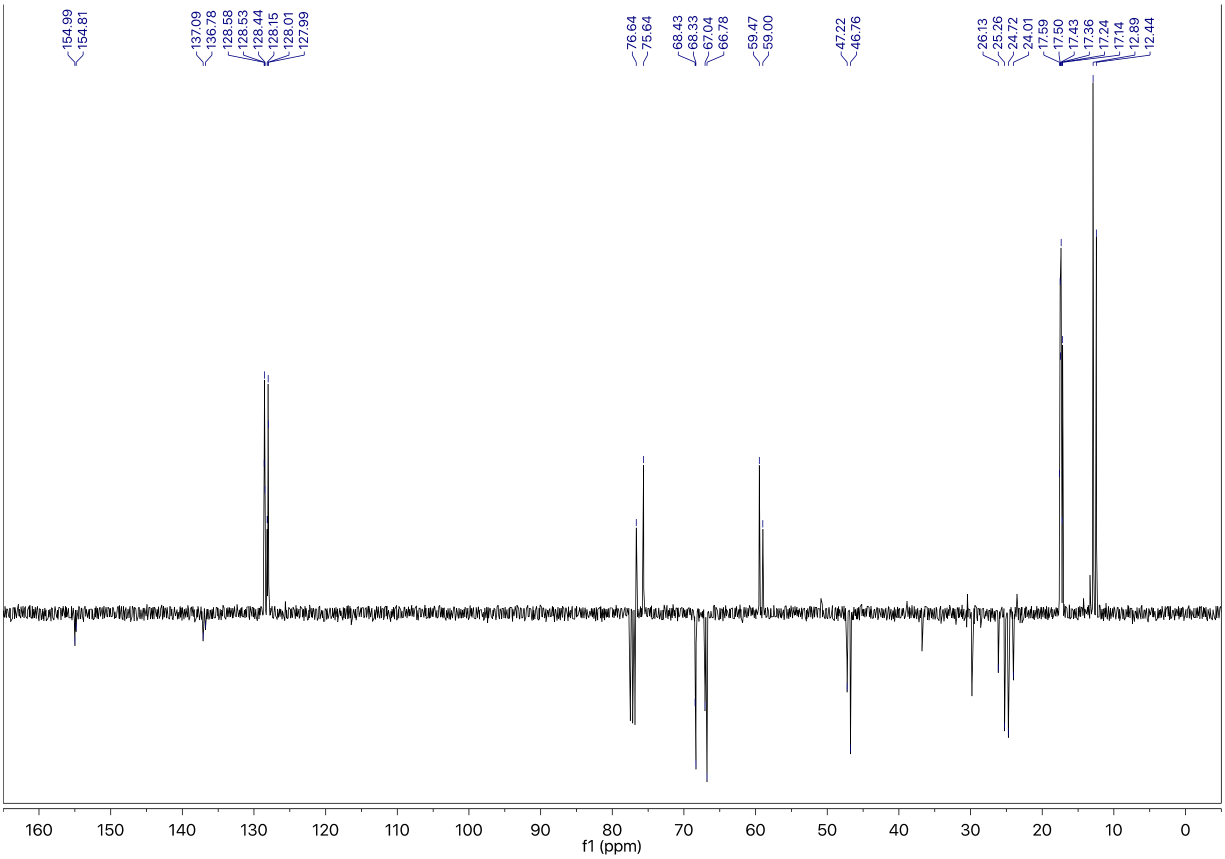

^{13}C NMR of **8**

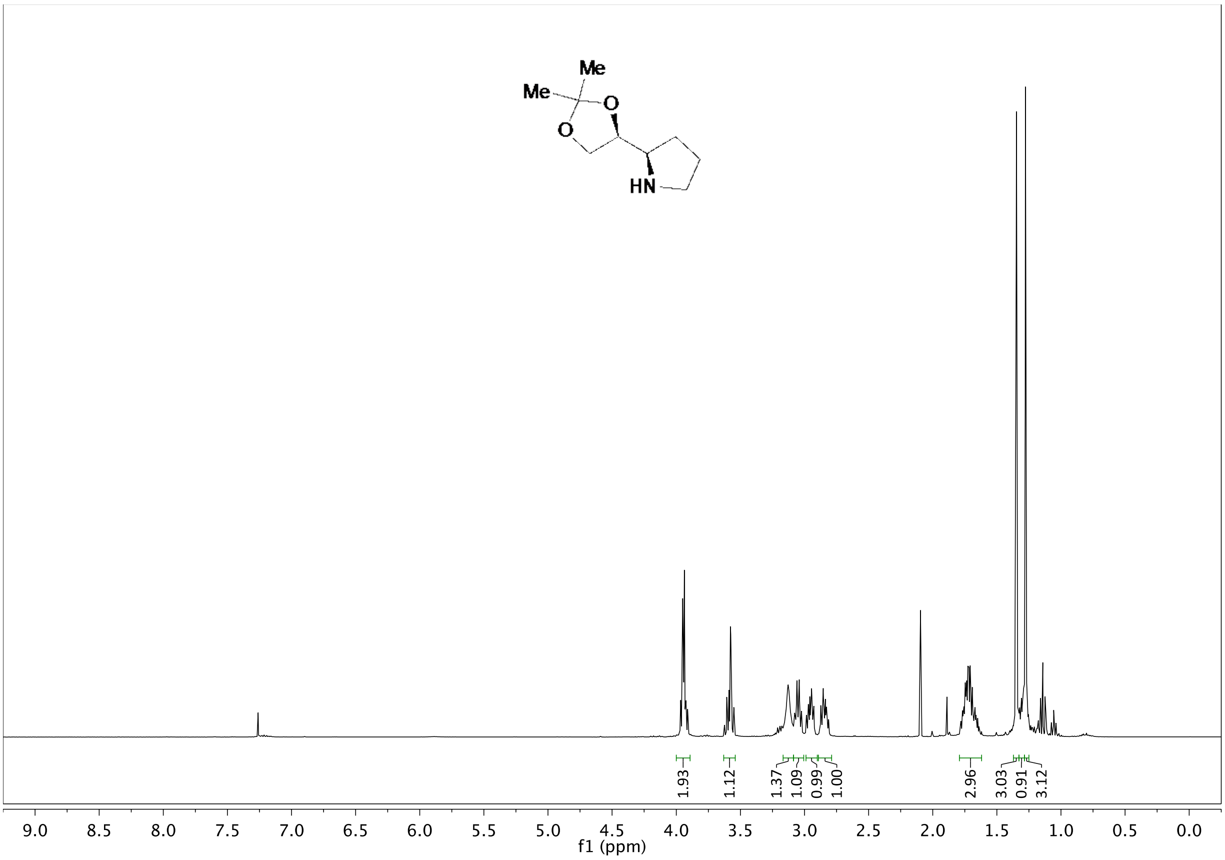

^{1}H NMR of **OC1**

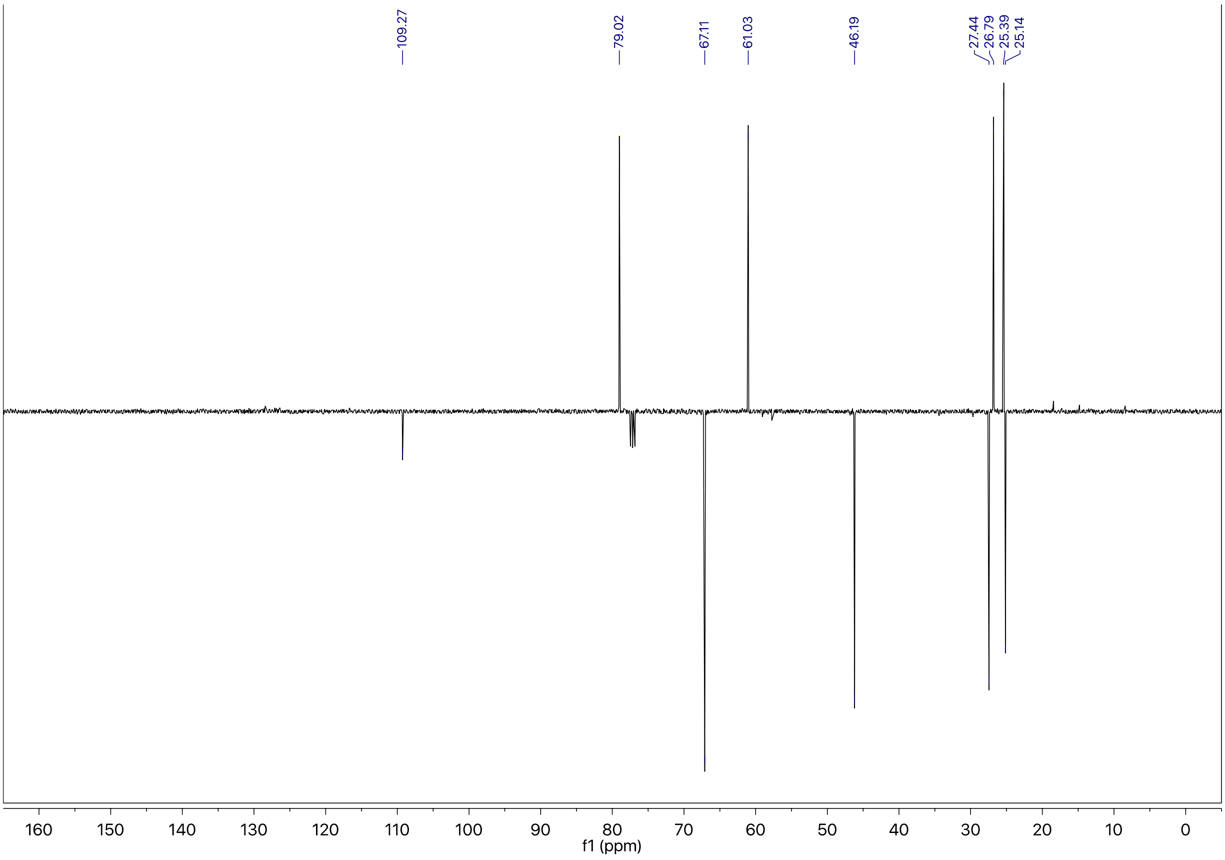

^{13}C NMR of **OC1**

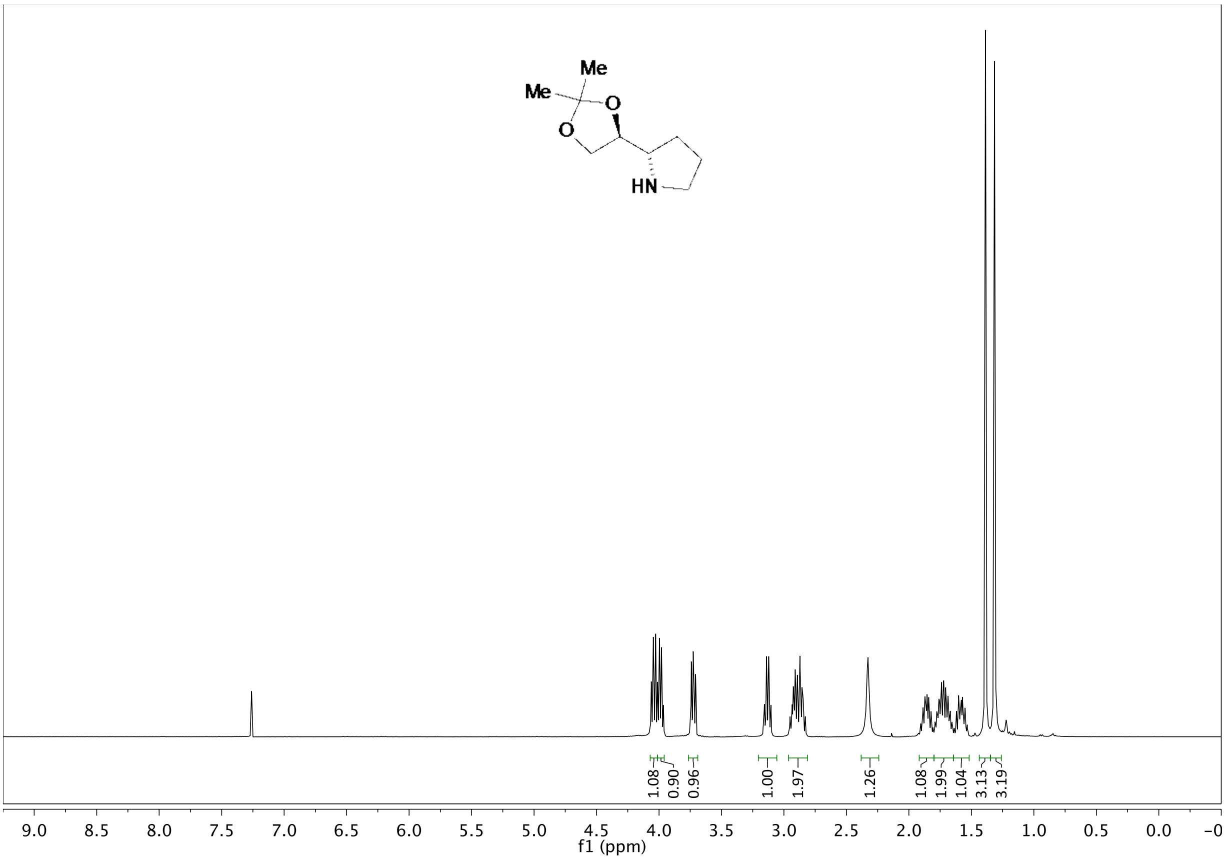

^{1}H NMR of **OC2**

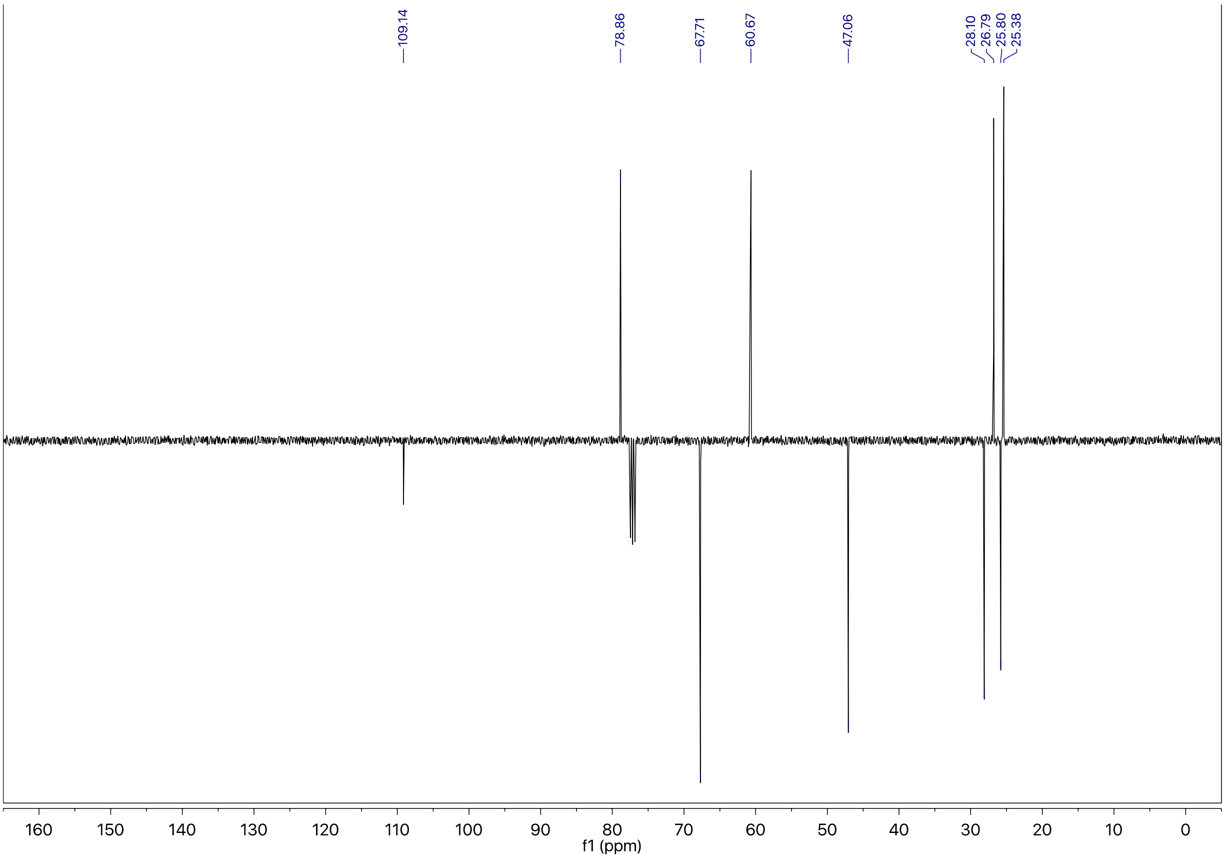

^{13}C NMR of **OC2**

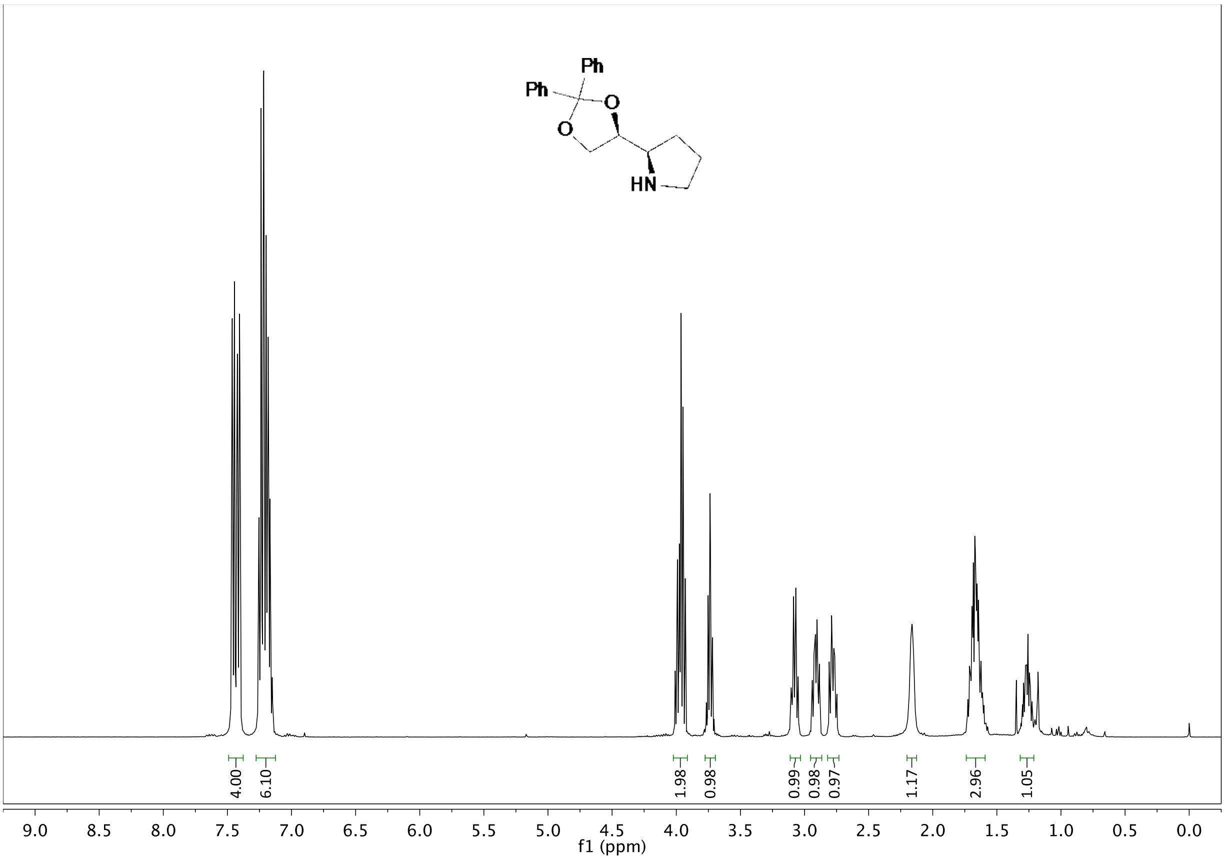

^{1}H NMR of **OC3**

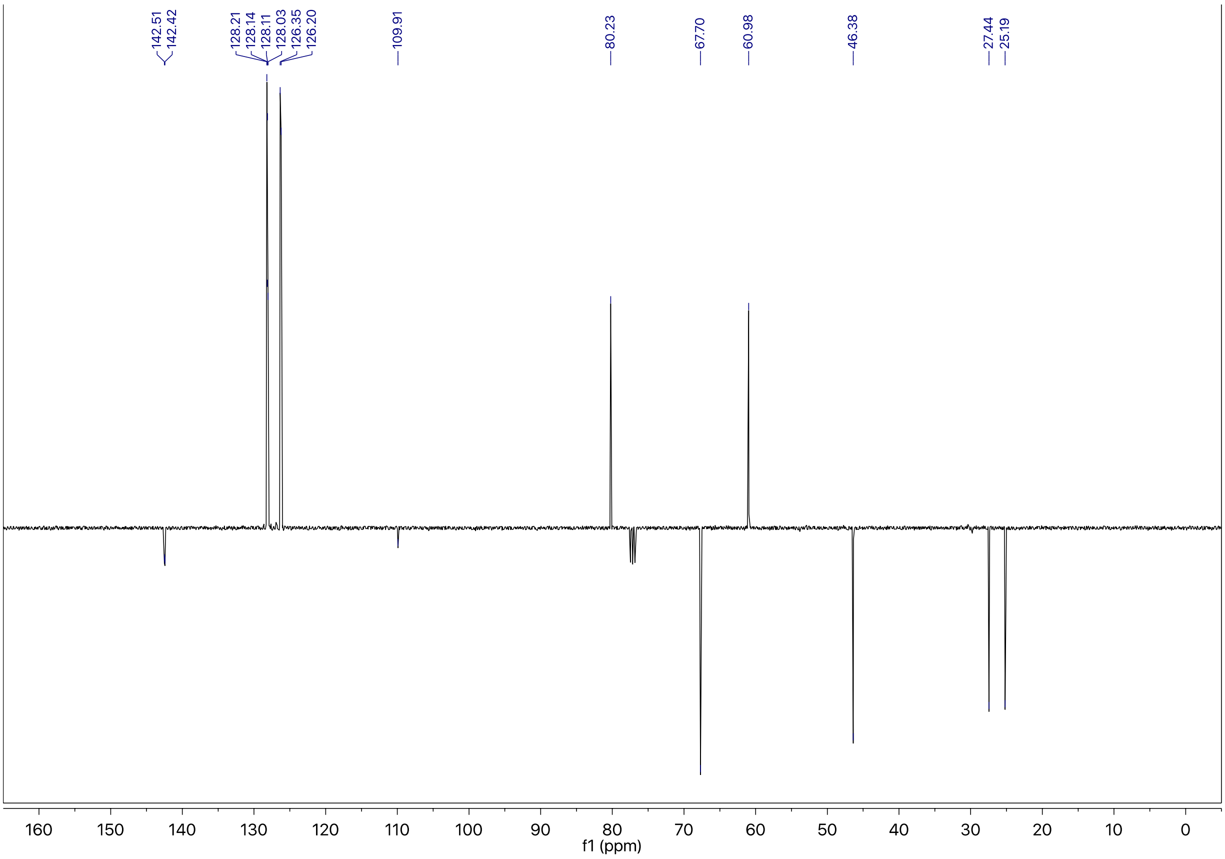

^{13}C NMR of **OC3**

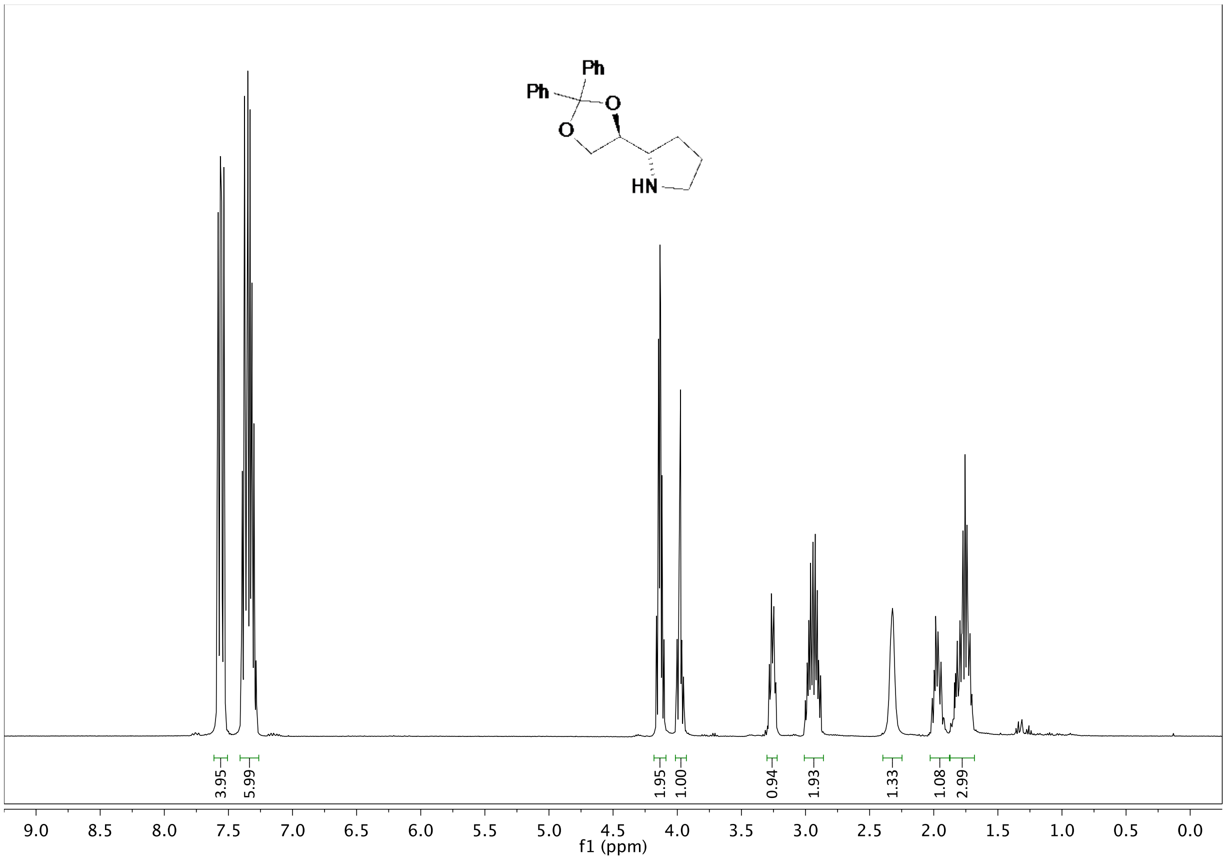

^{1}H NMR of **OC4**

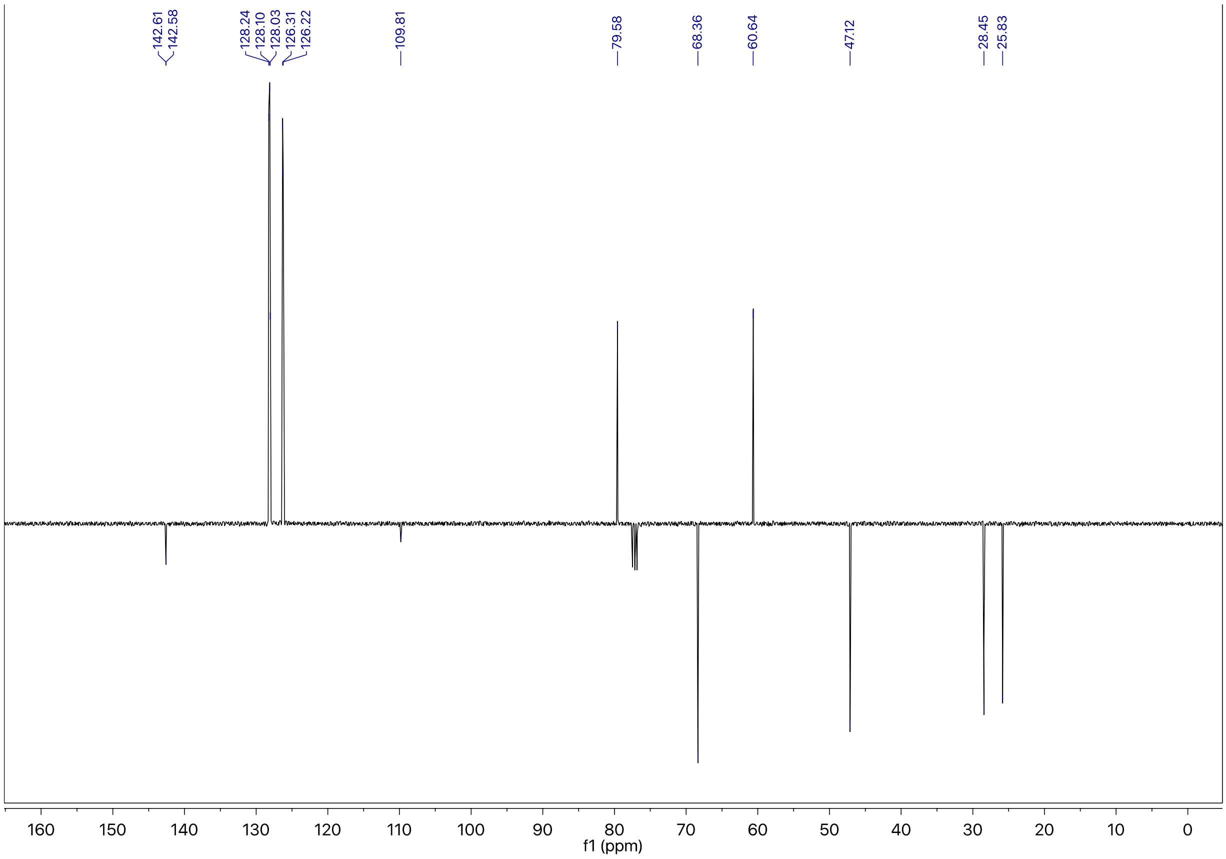

^{13}C NMR of **OC4**

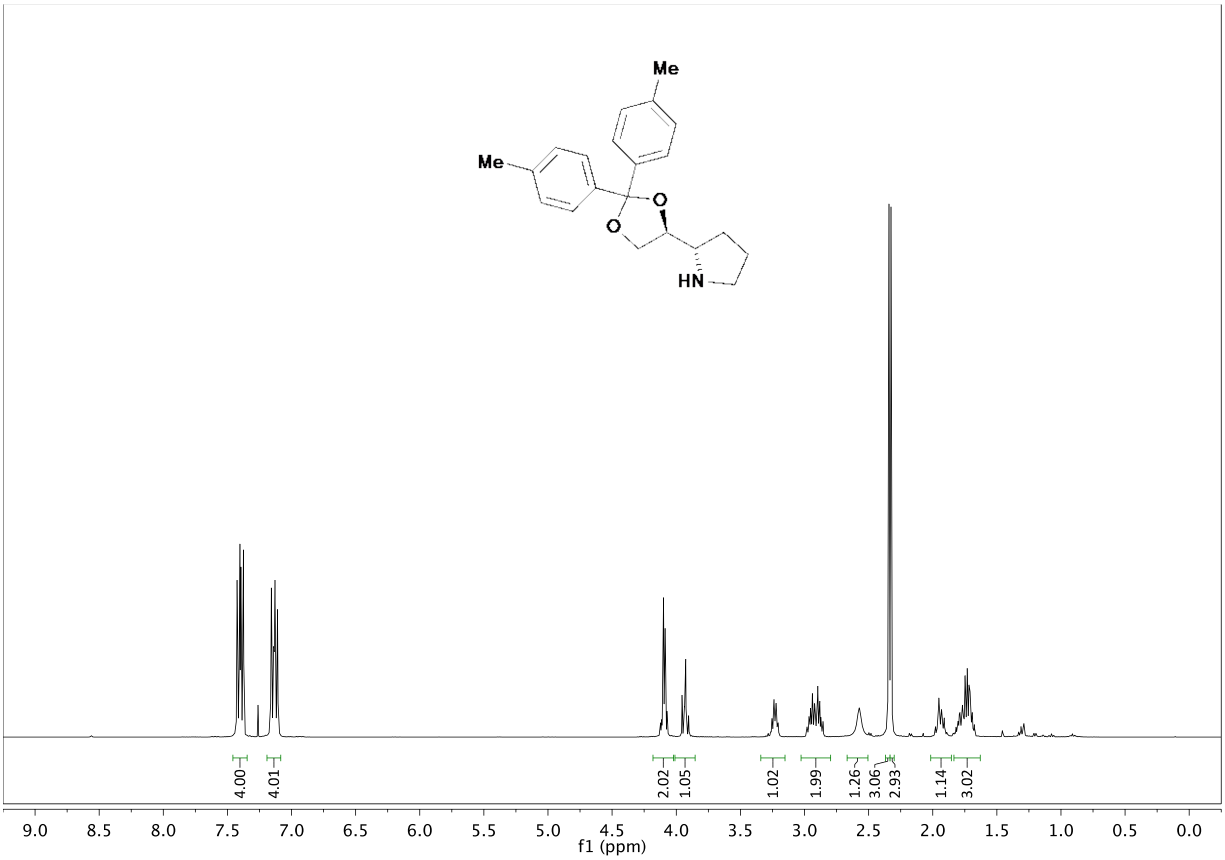

^{1}H NMR of **OC5**

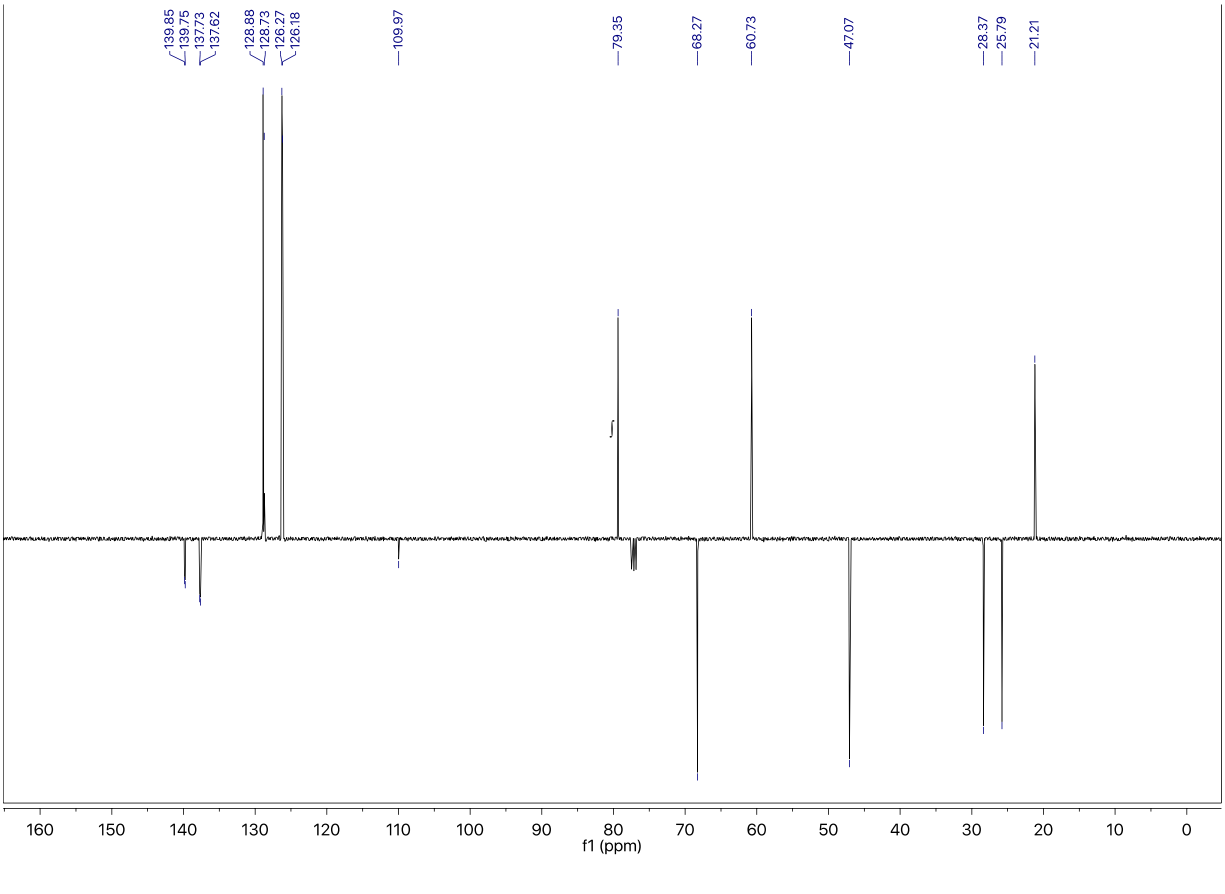

^{13}C NMR of **OC5**

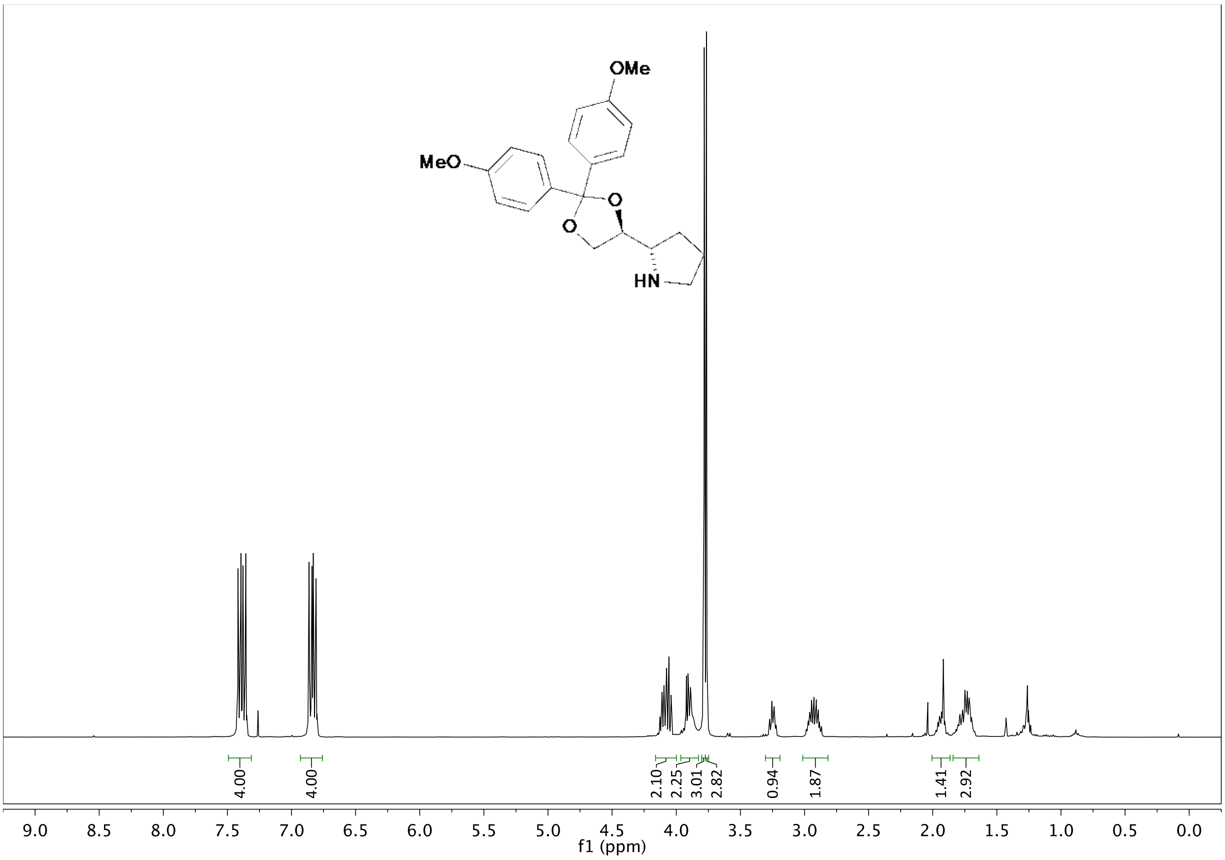

^{1}H NMR of **OC6**

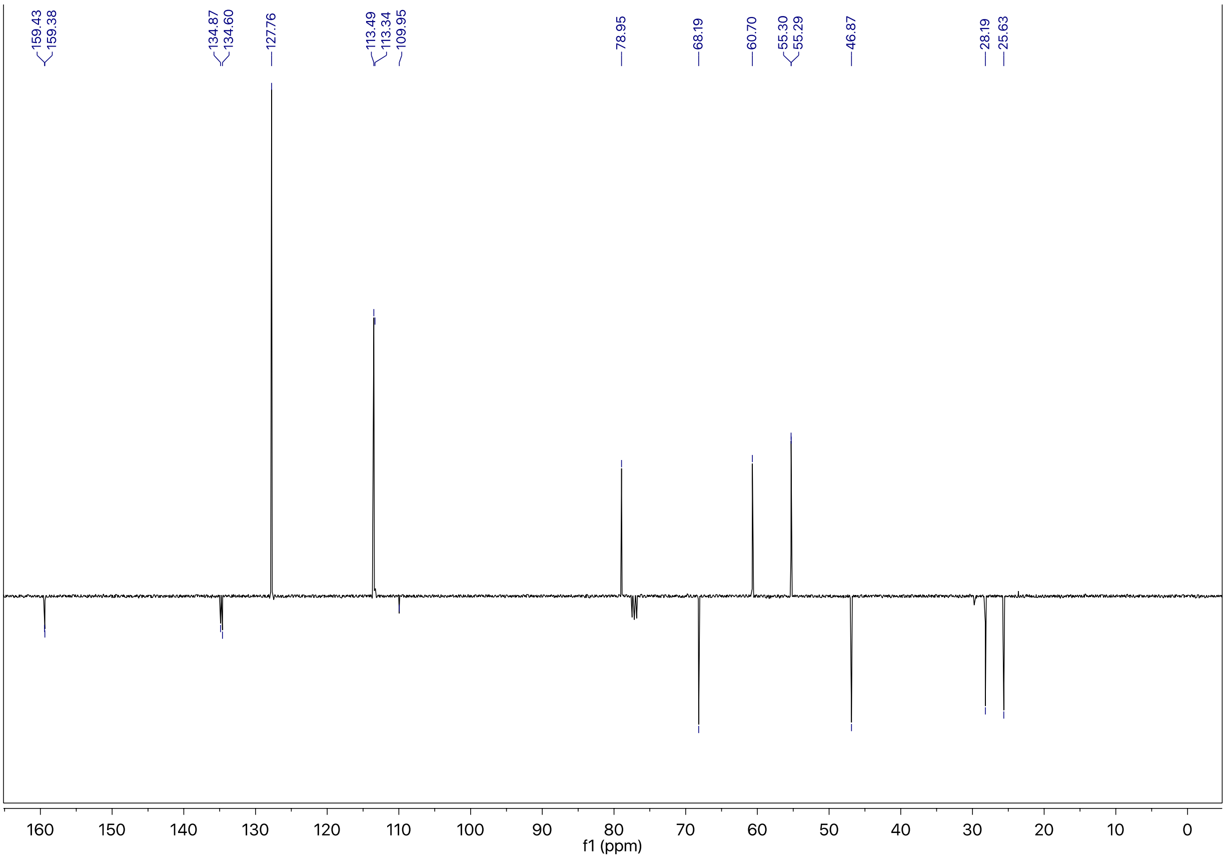

^{13}C NMR of **OC6**

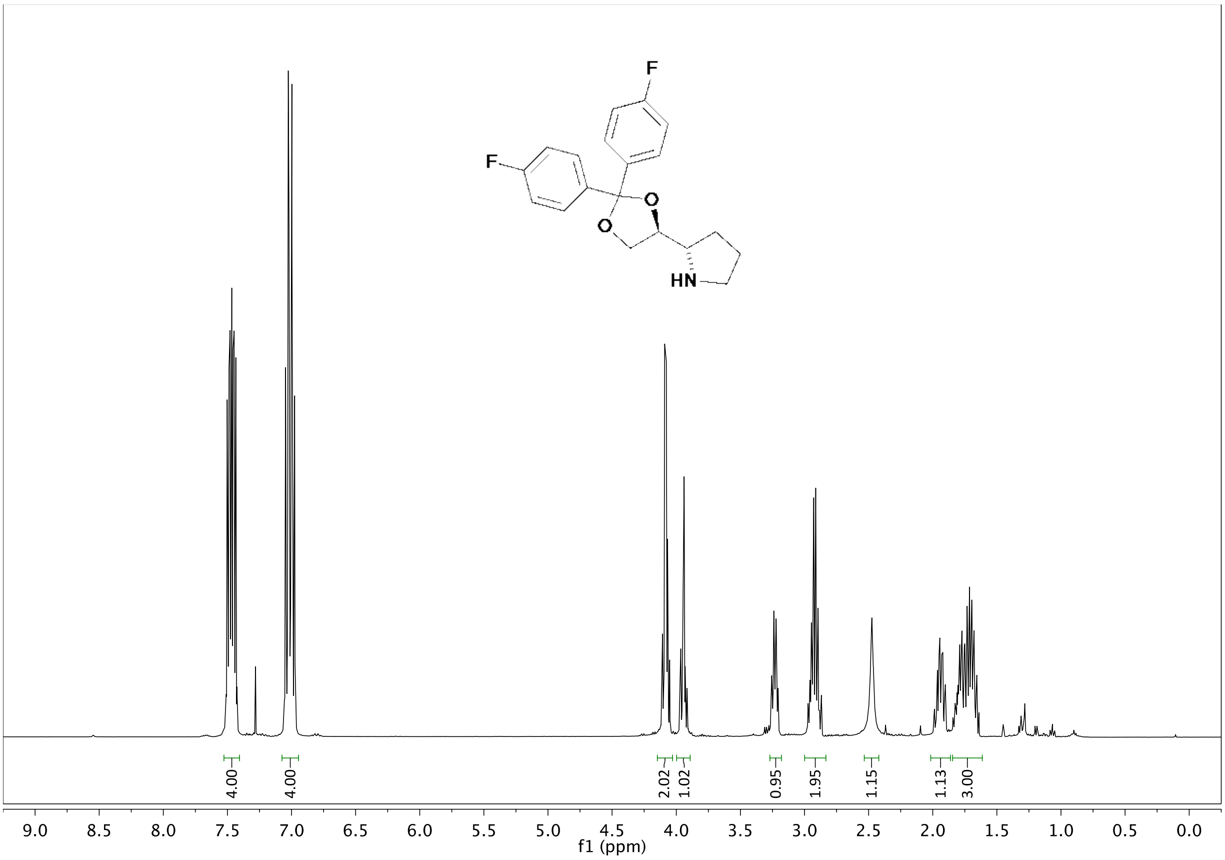

^{1}H NMR of **OC7**

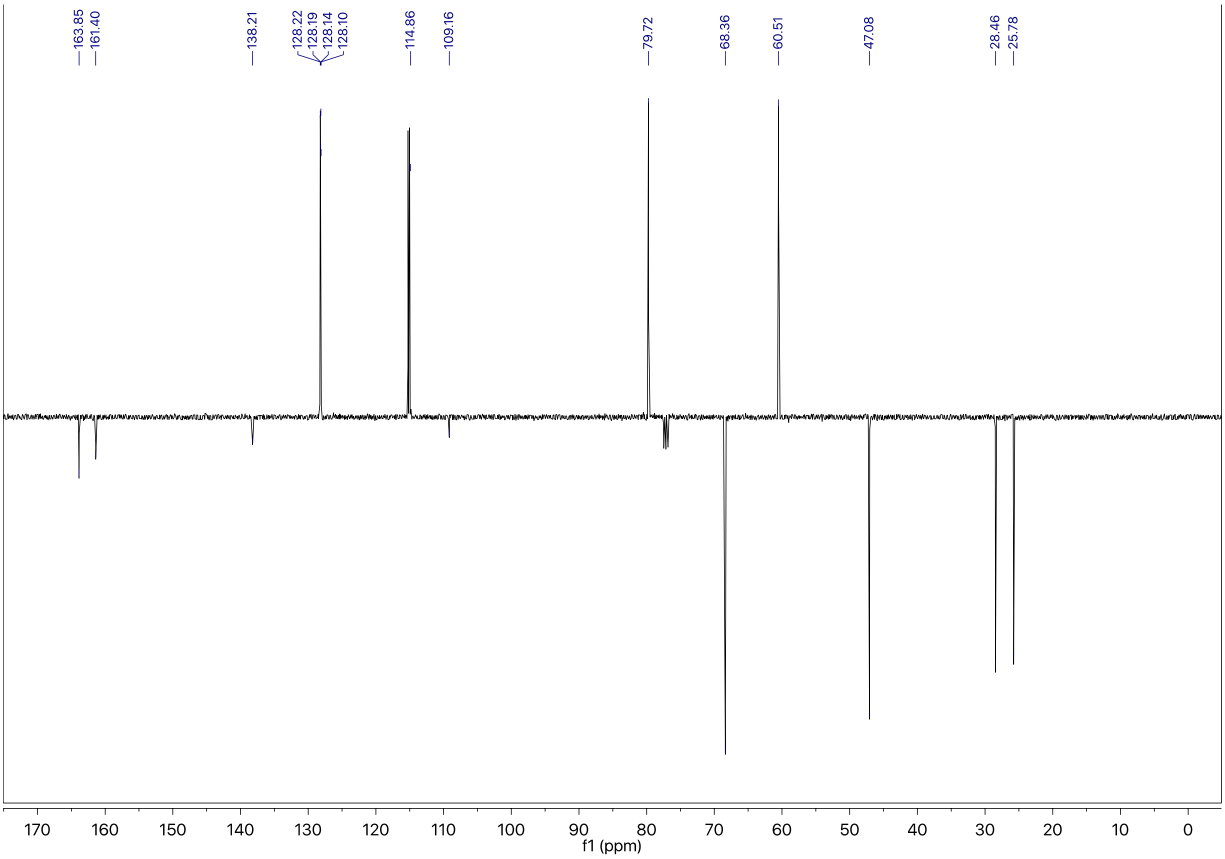

^{13}C NMR of **OC7**

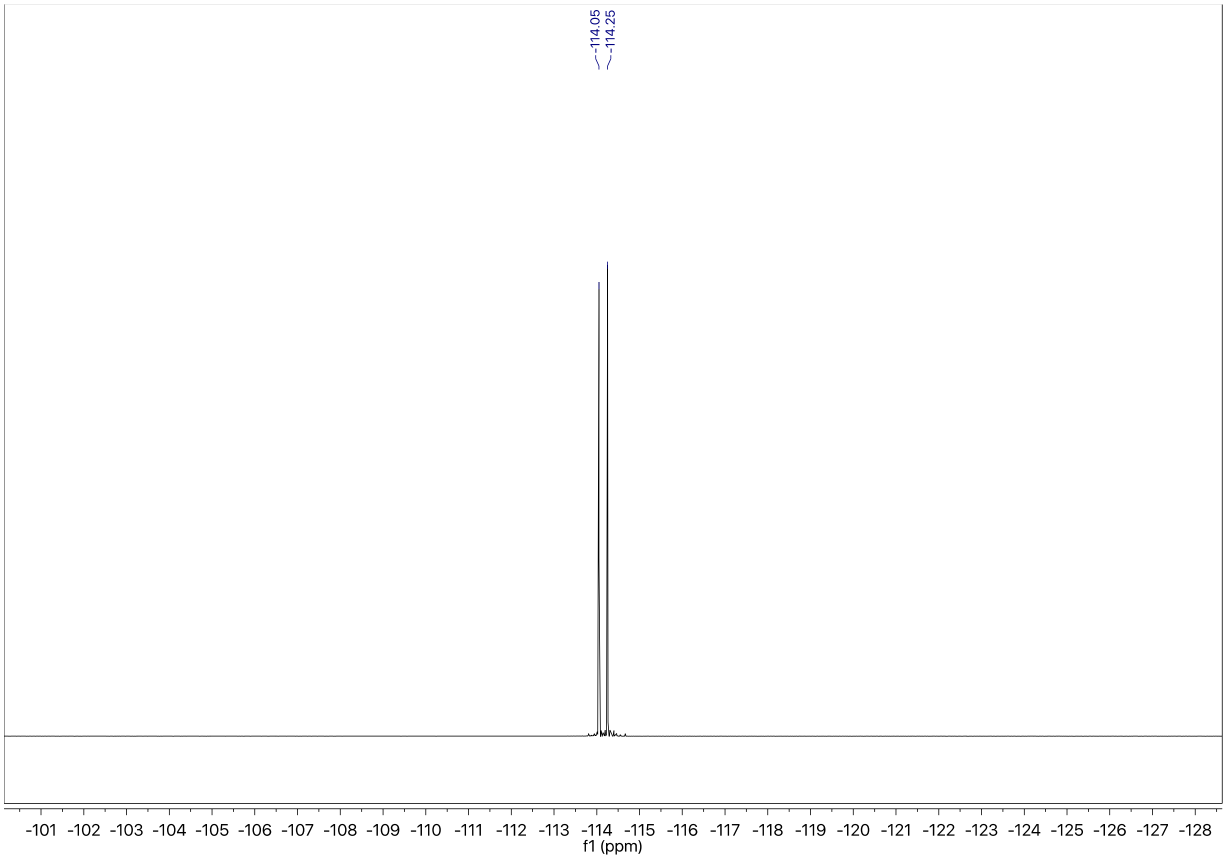

^{19}F NMR of **OC7**

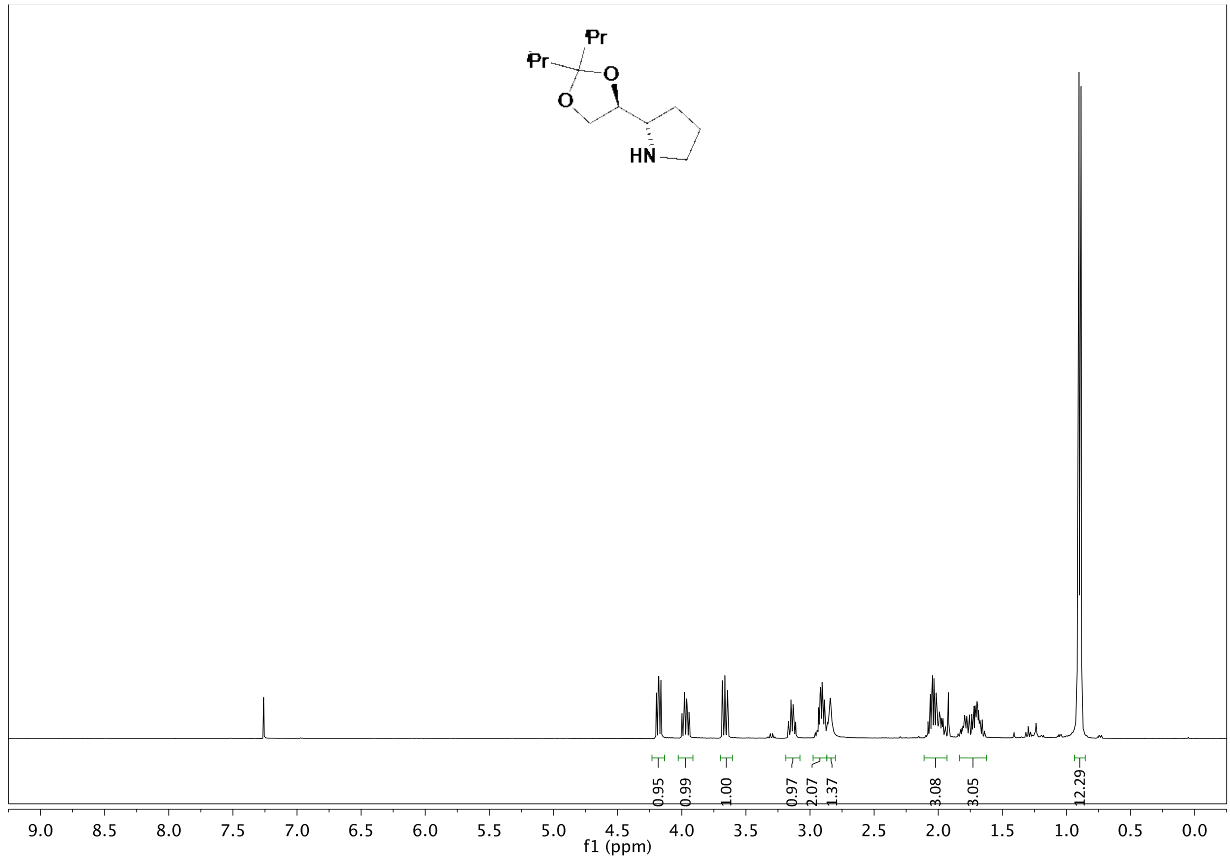

^{1}H NMR of **OC8**

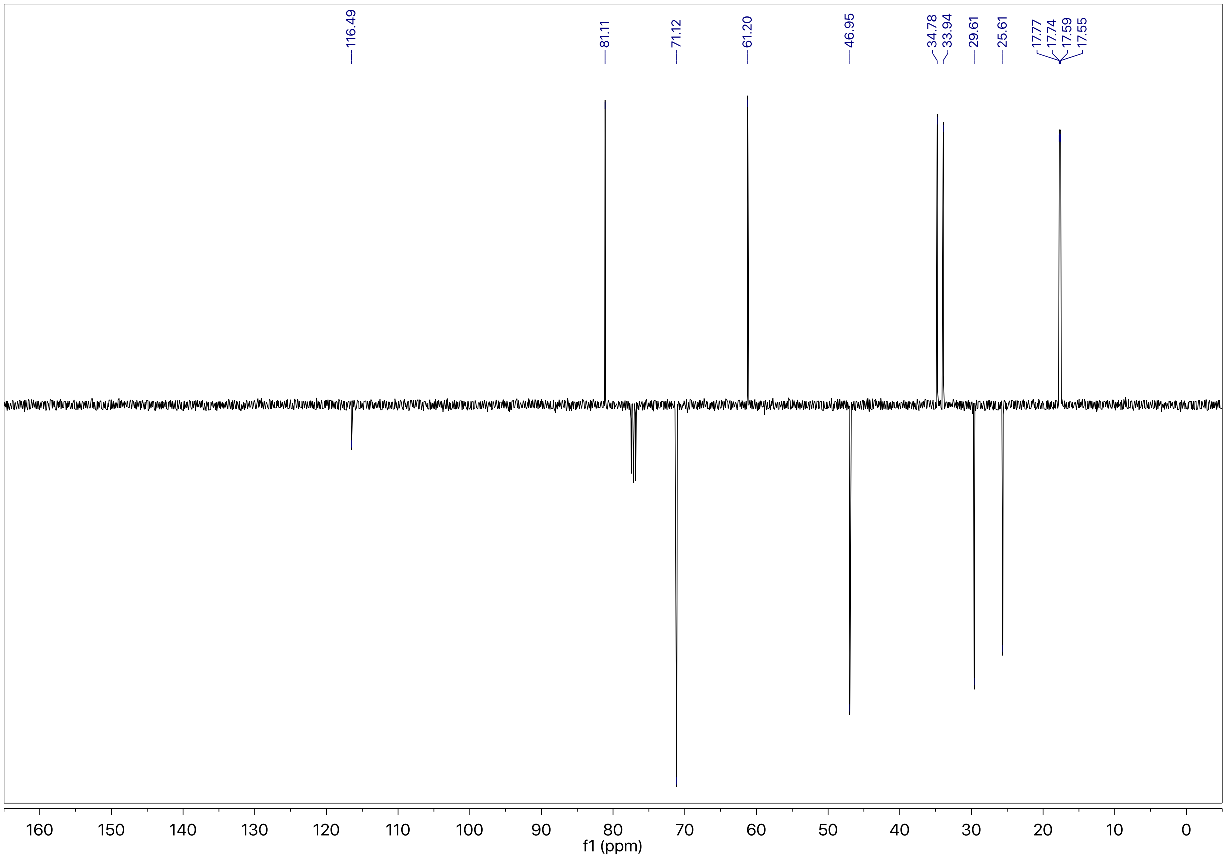

^{13}C NMR of **OC8**

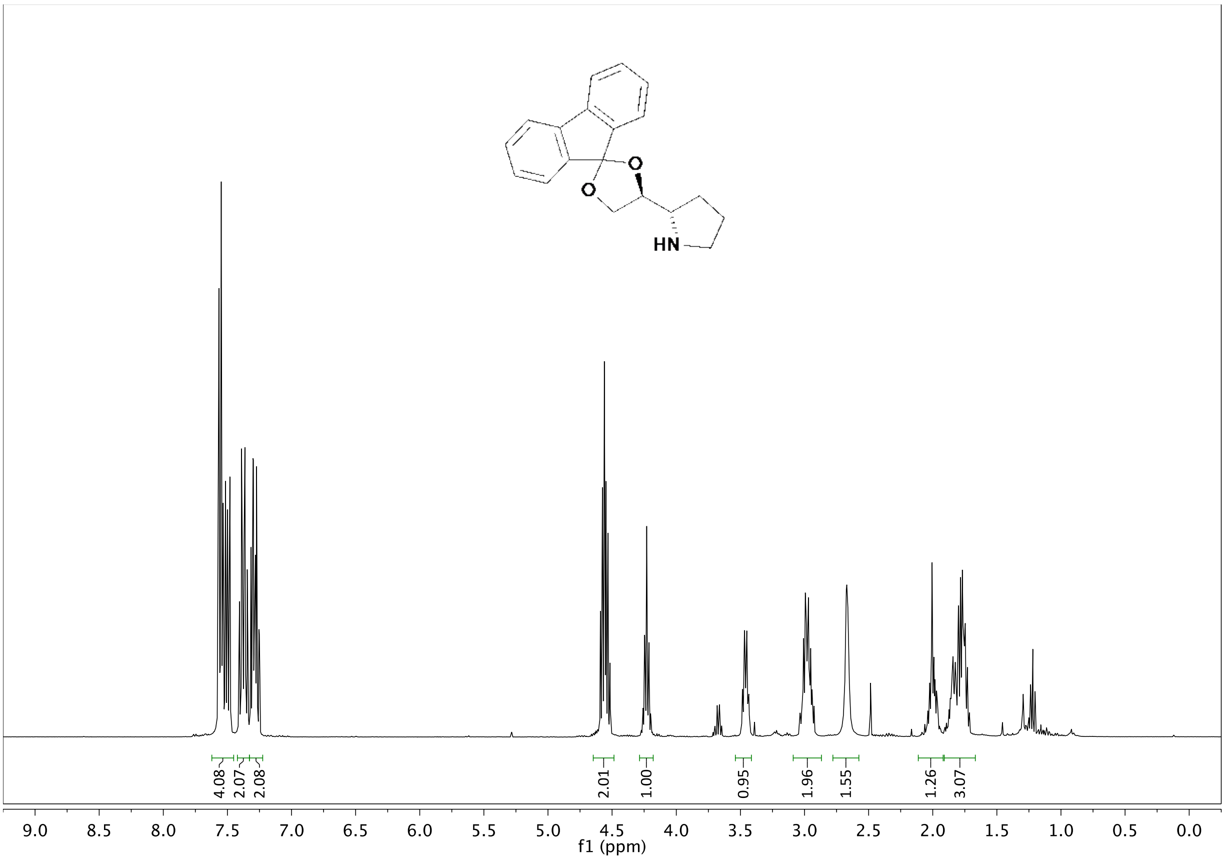

^{1}H NMR of **OC9**

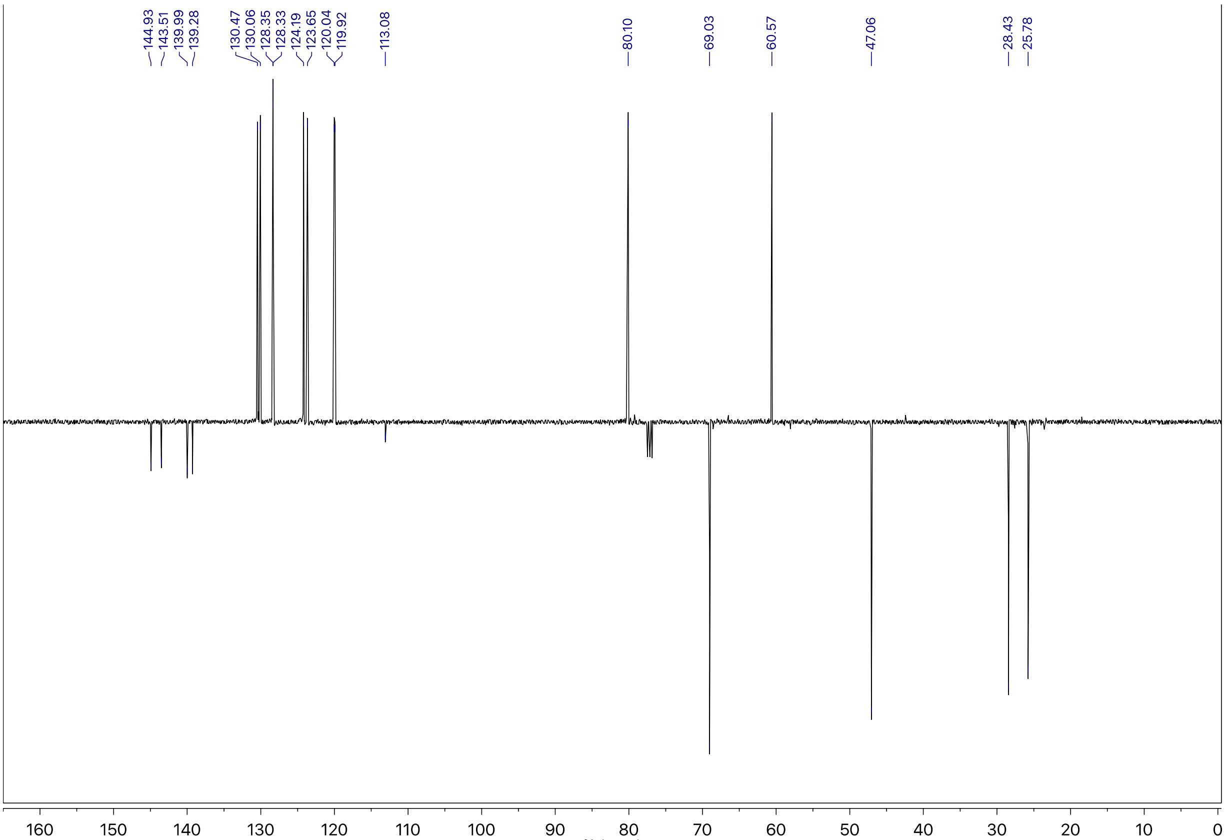

^{13}C NMR of **OC9**

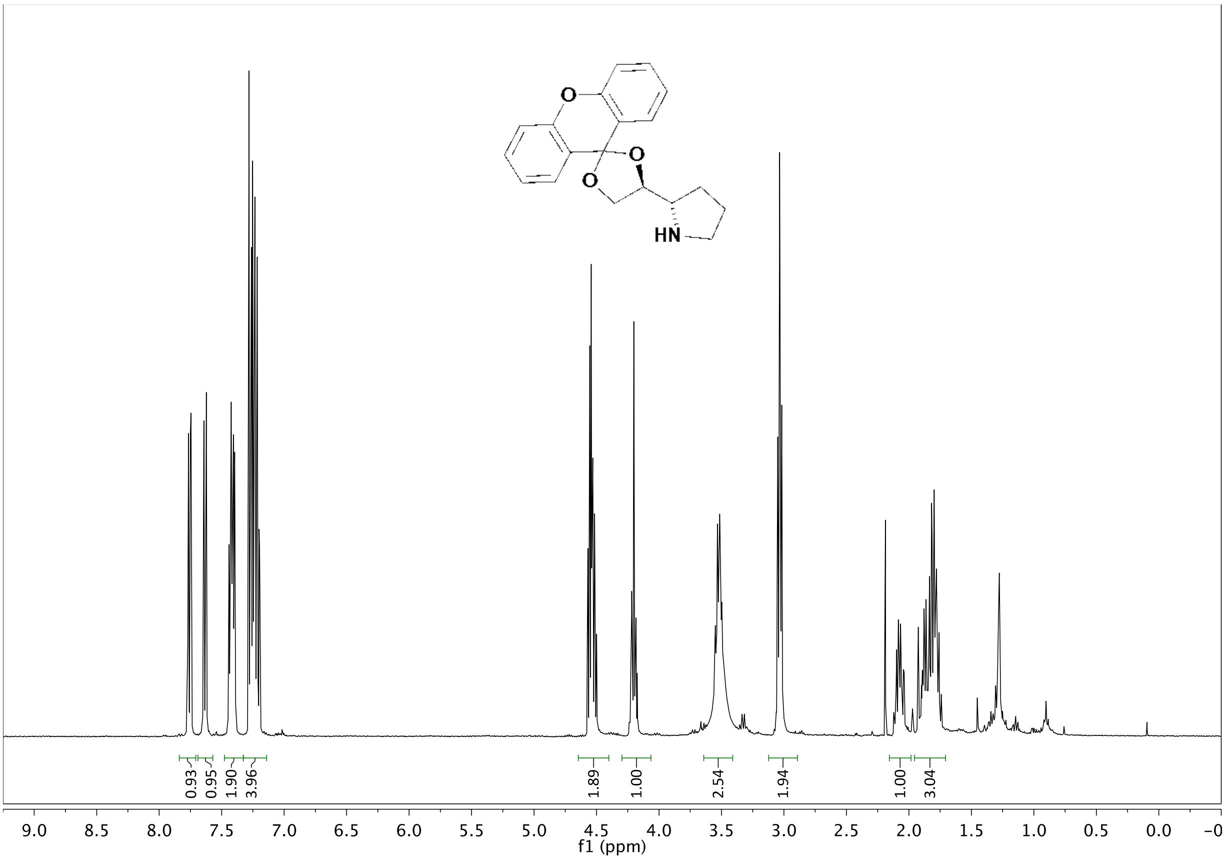

^{1}H NMR of **OC10**

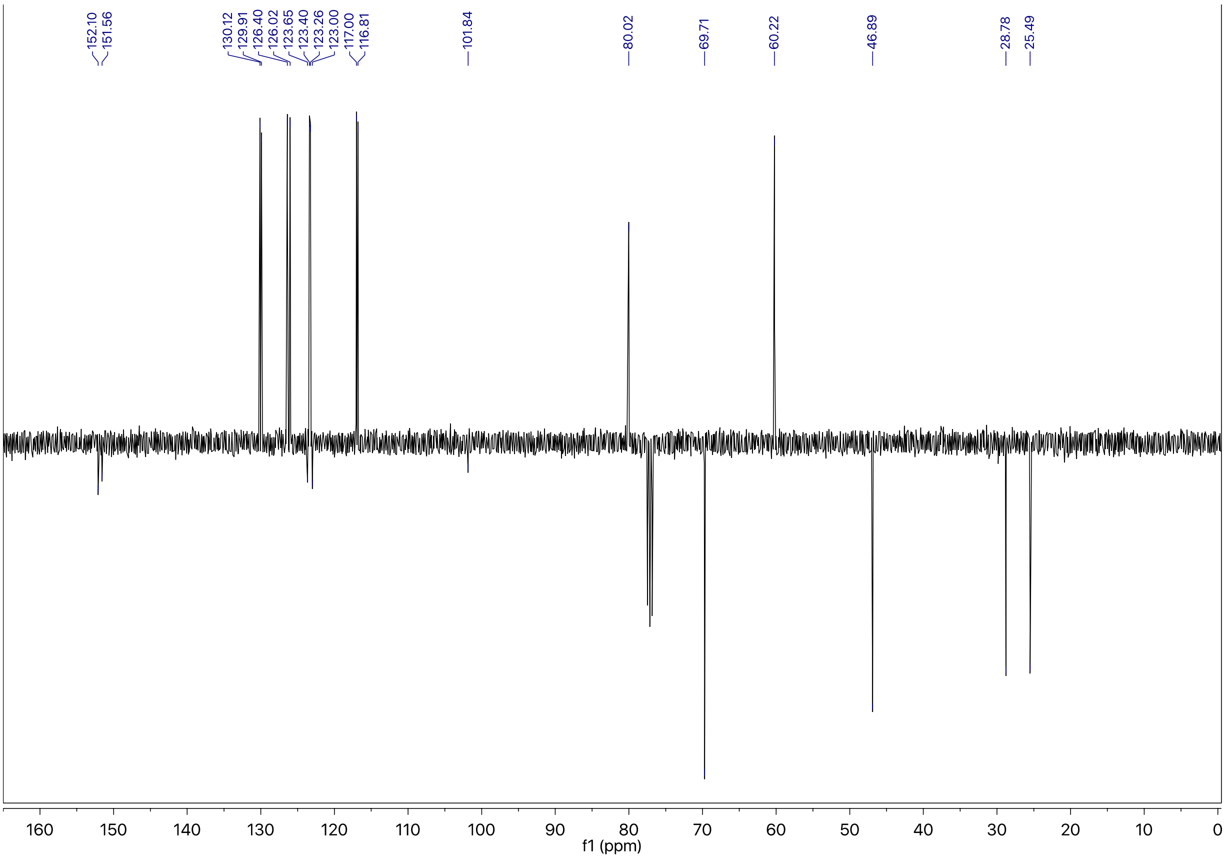

^{13}C NMR of **OC10**

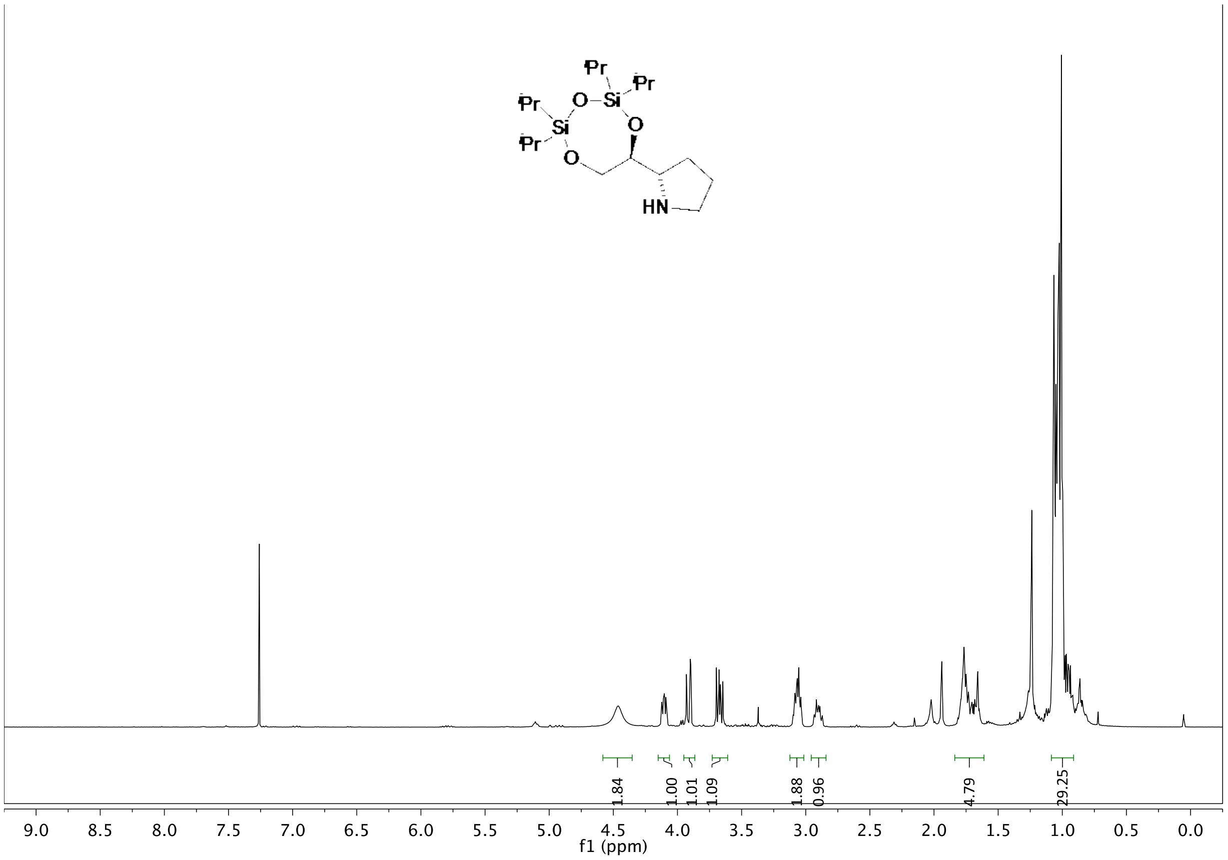

^{1}H NMR of **OC11**

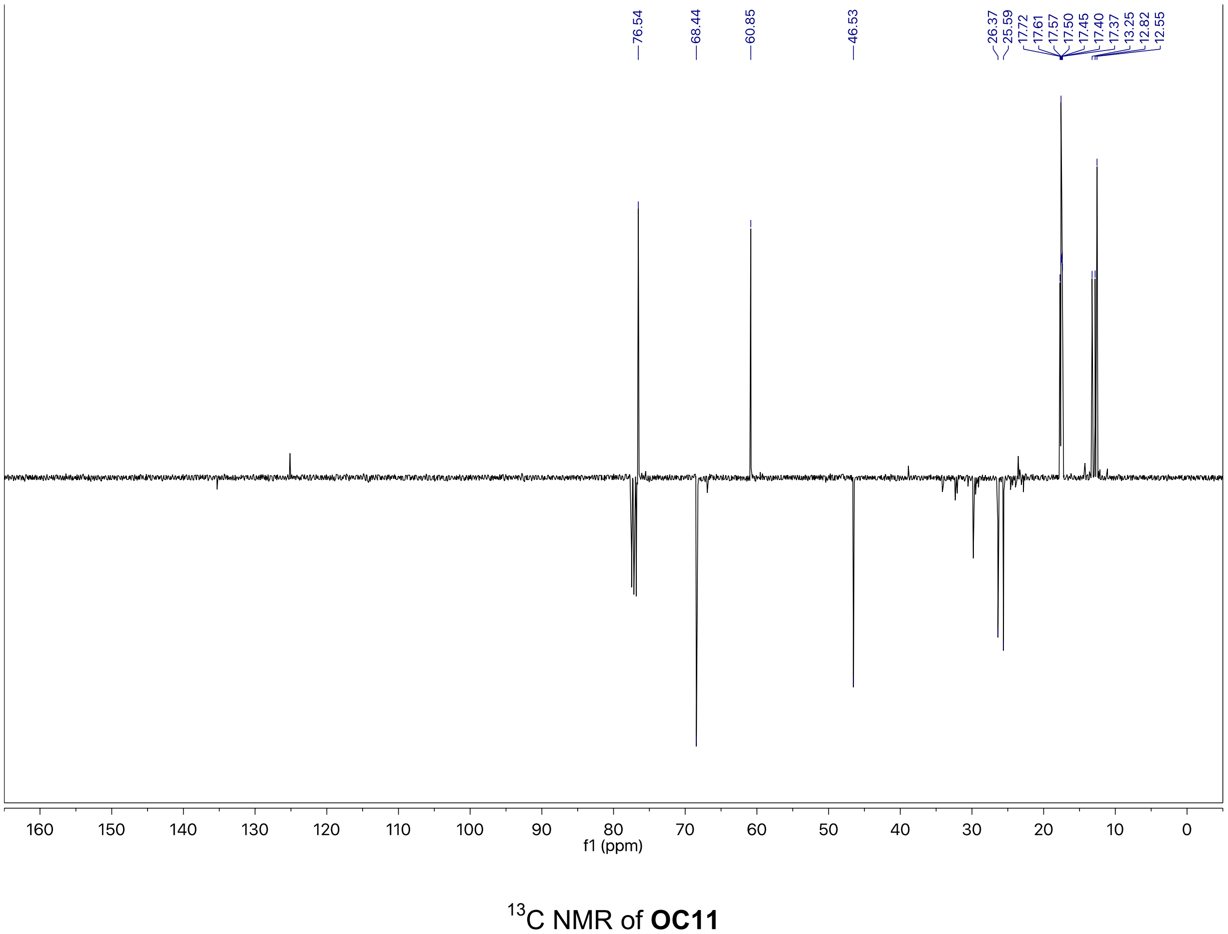

^{13}C NMR of **OC11**

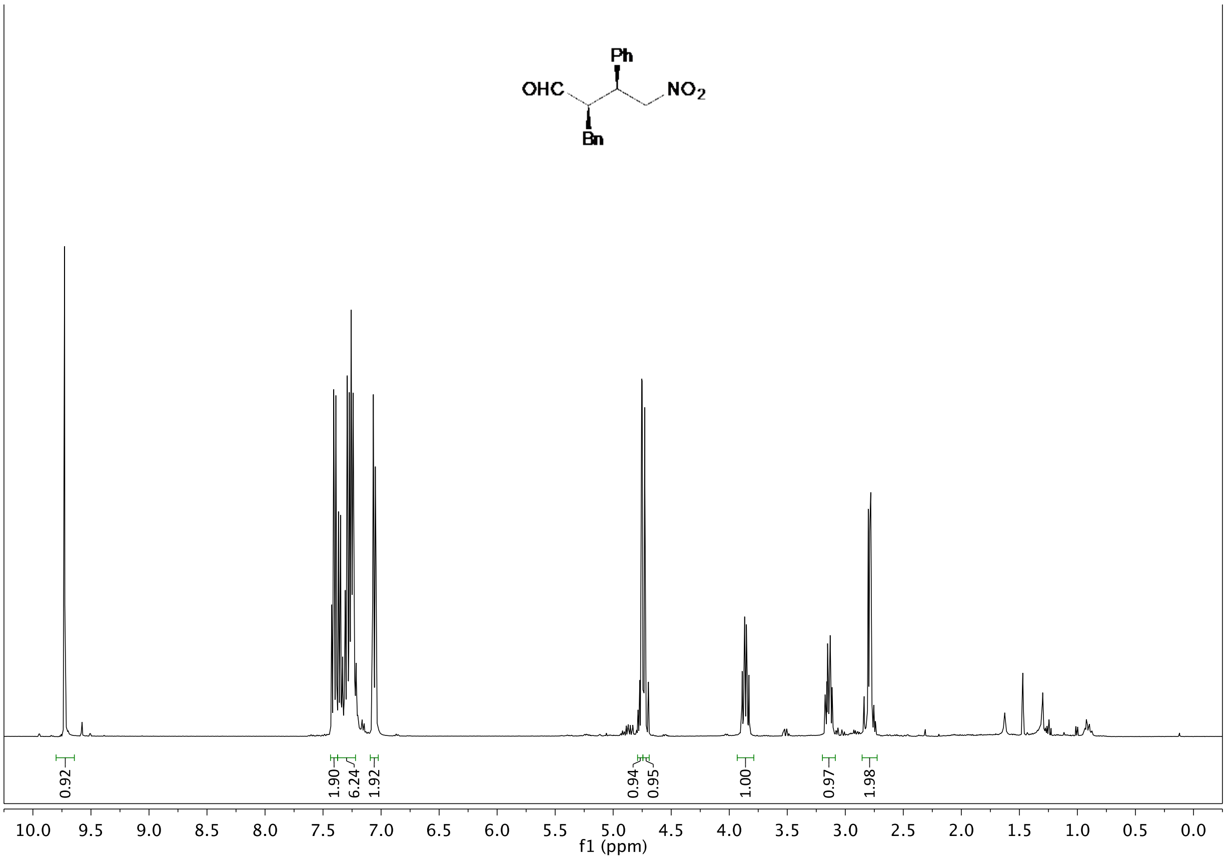

^{1}H NMR of (2*R*,3*S*)-2-benzyl-4-nitro-3-phenylbutanal **9a**

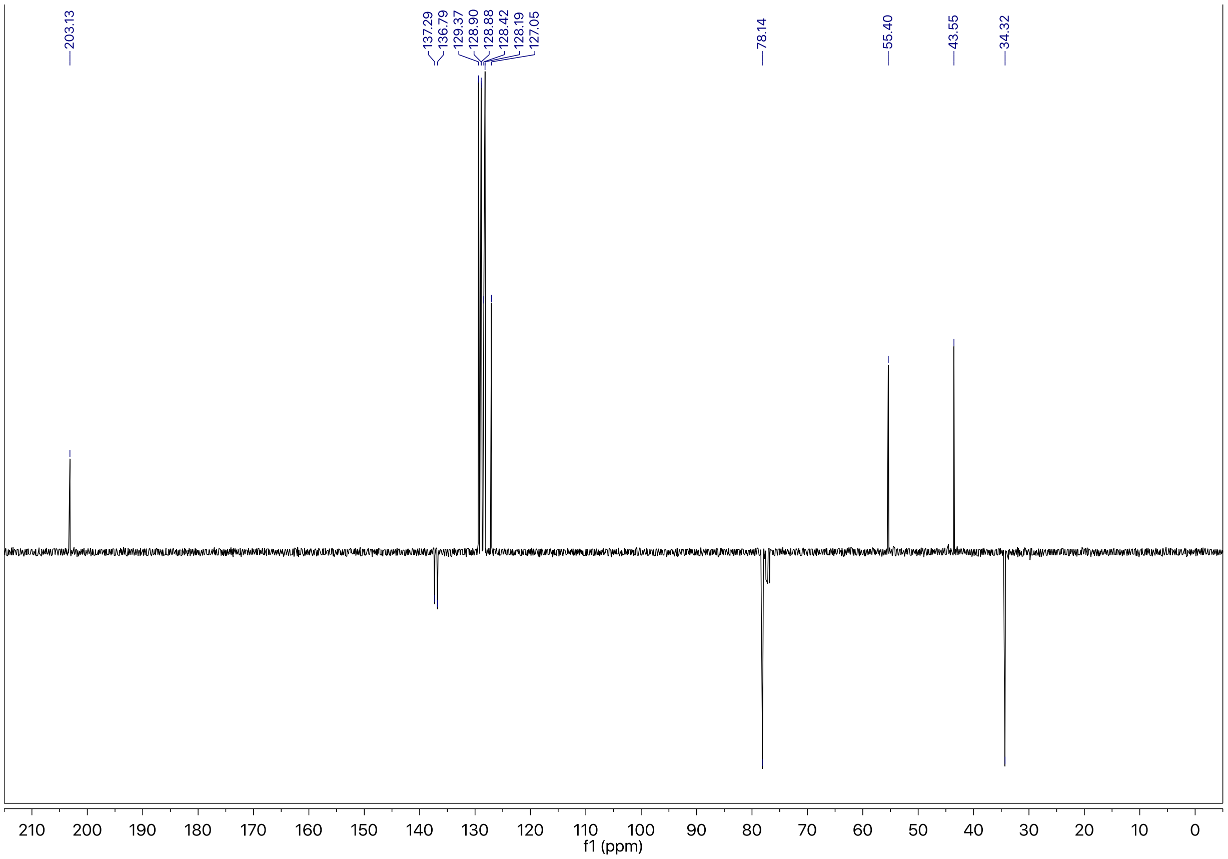

^{13}C NMR of (2*R*,3*S*)-2-benzyl-4-nitro-3-phenylbutanal **9a**

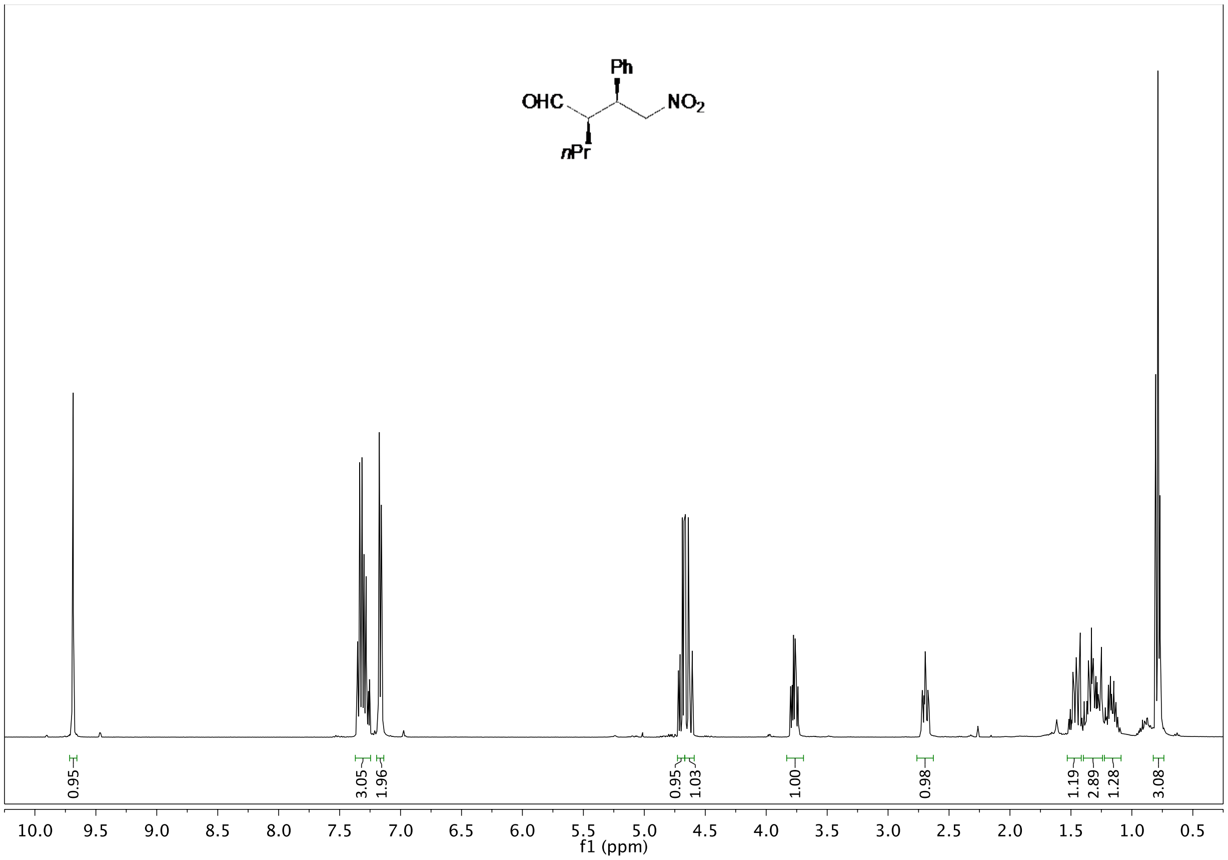

^{1}H NMR of (*R*)-2-[(*S*)-2-nitro-1-phenylethyl]pentanal **9b**

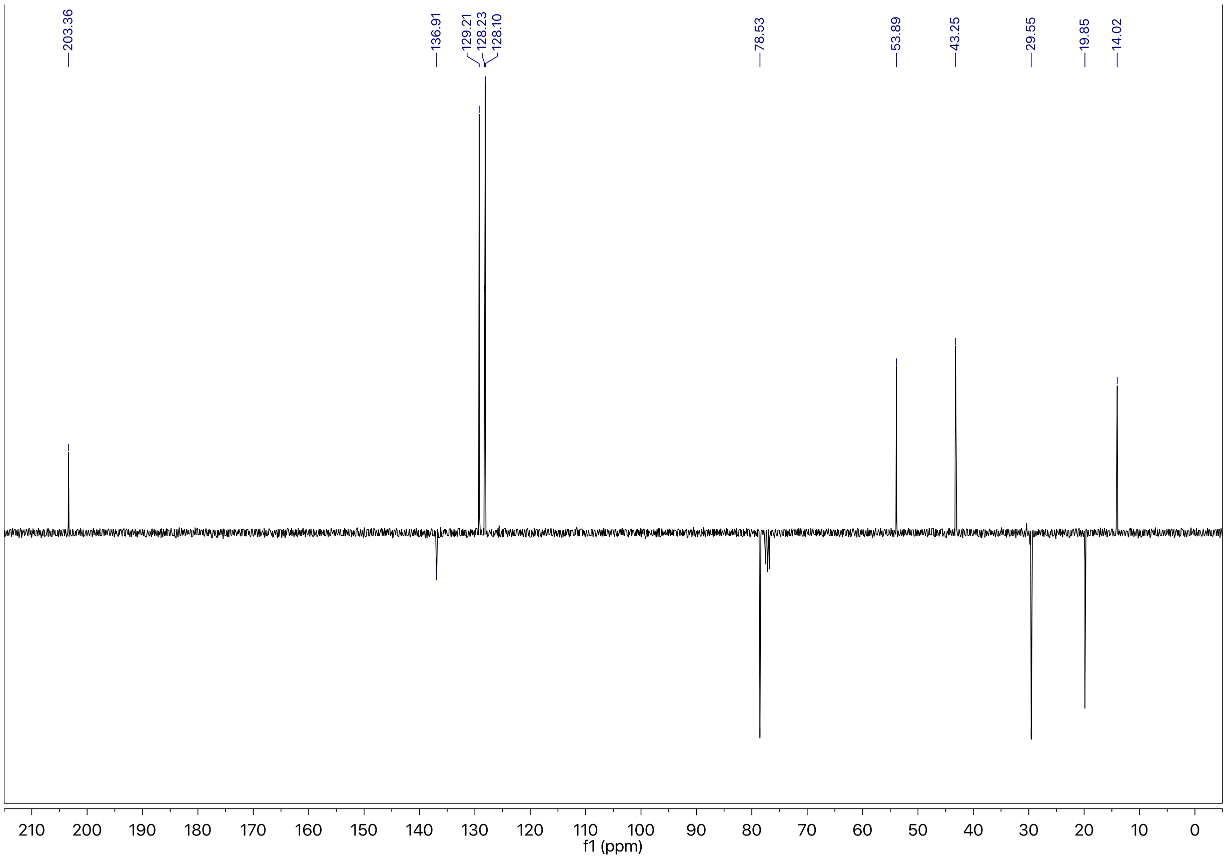

^{13}C NMR of (*R*)-2-[(*S*)-2-nitro-1-phenylethyl]pentanal **9b**

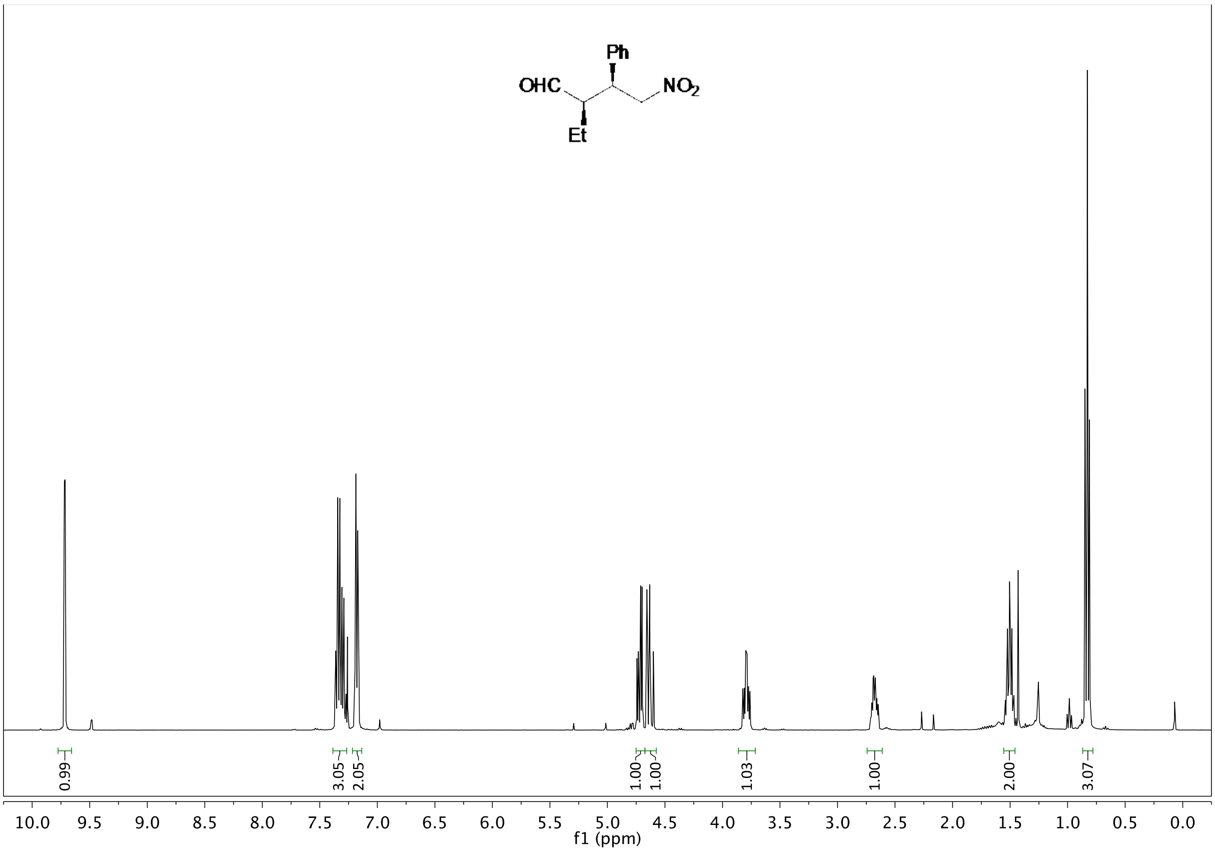

^{1}H NMR of (2*R*,3*S*)-2-ethyl-4-nitro-3-phenylbutanal **9c**

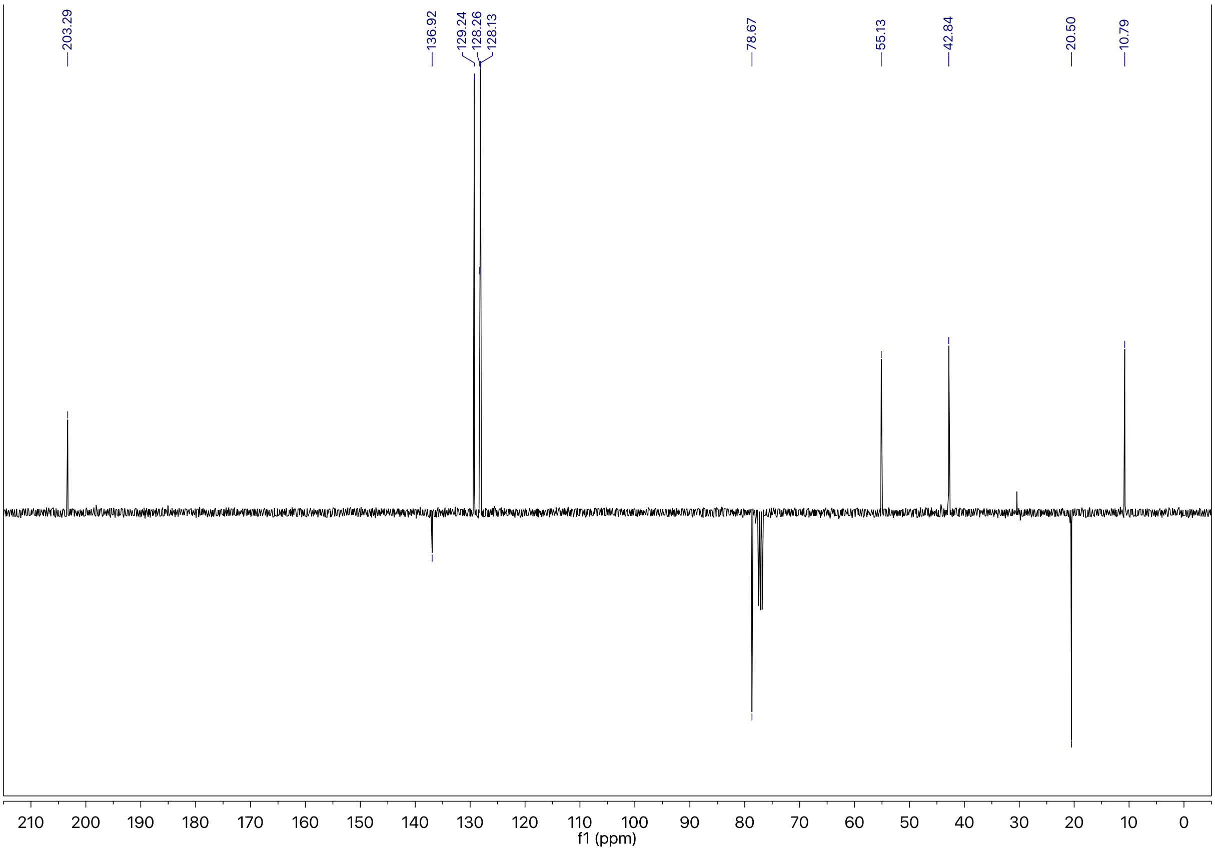

^{13}C NMR of (2*R*,3*S*)-2-ethyl-4-nitro-3-phenylbutanal **9c**

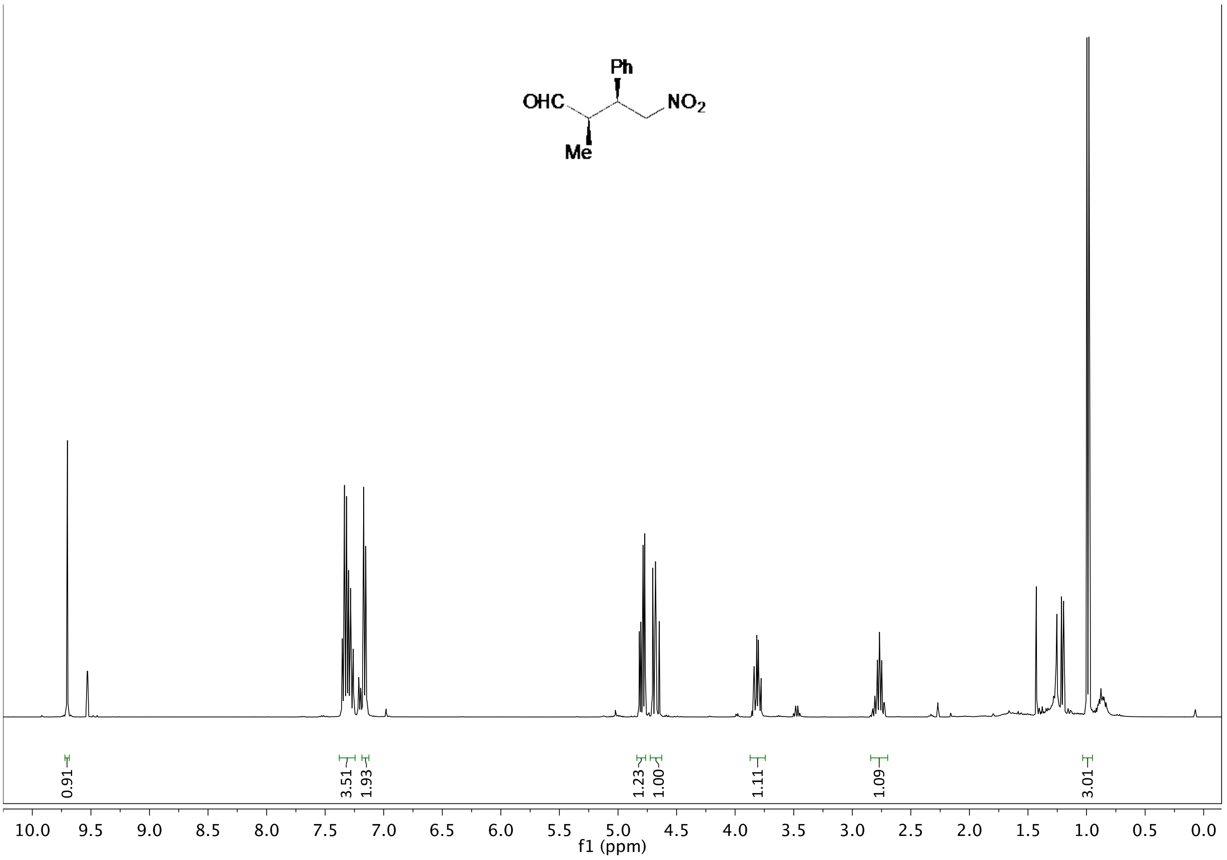

^{1}H NMR of (2*R*,3*S*)-2-methyl-4-nitro-3-phenylbutanal **9d**

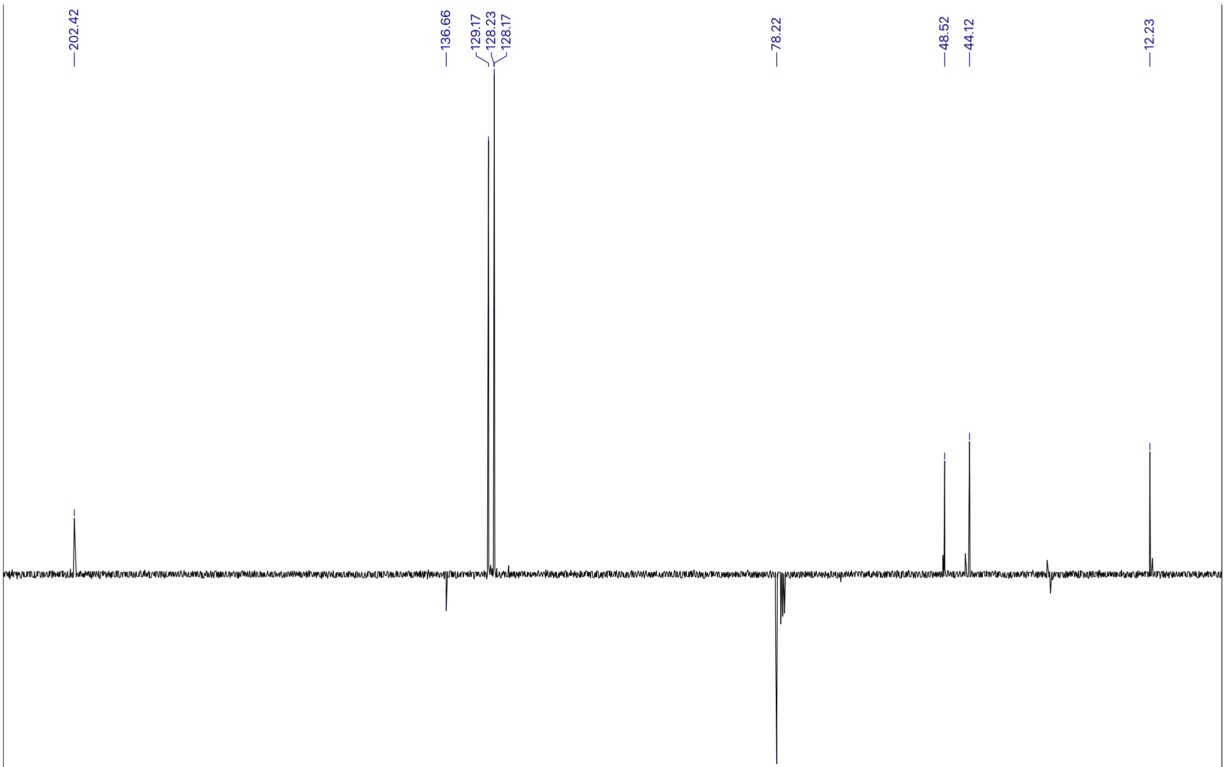

^{13}C NMR of (2*R*,3*S*)-2-methyl-4-nitro-3-phenylbutanal **9d**

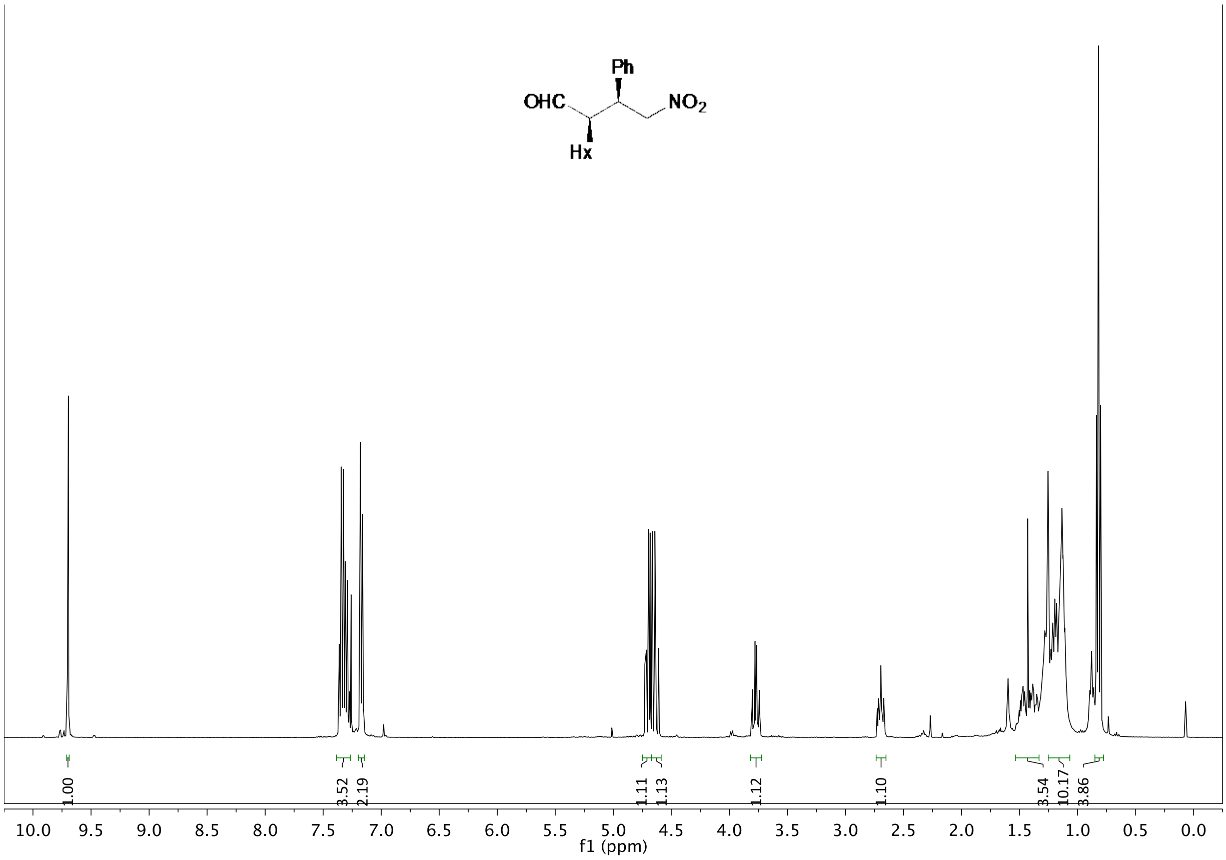

^{1}H NMR of (*R*)-2-[(*S*)-2-nitro-1-phenylethyl]octanal **9e**

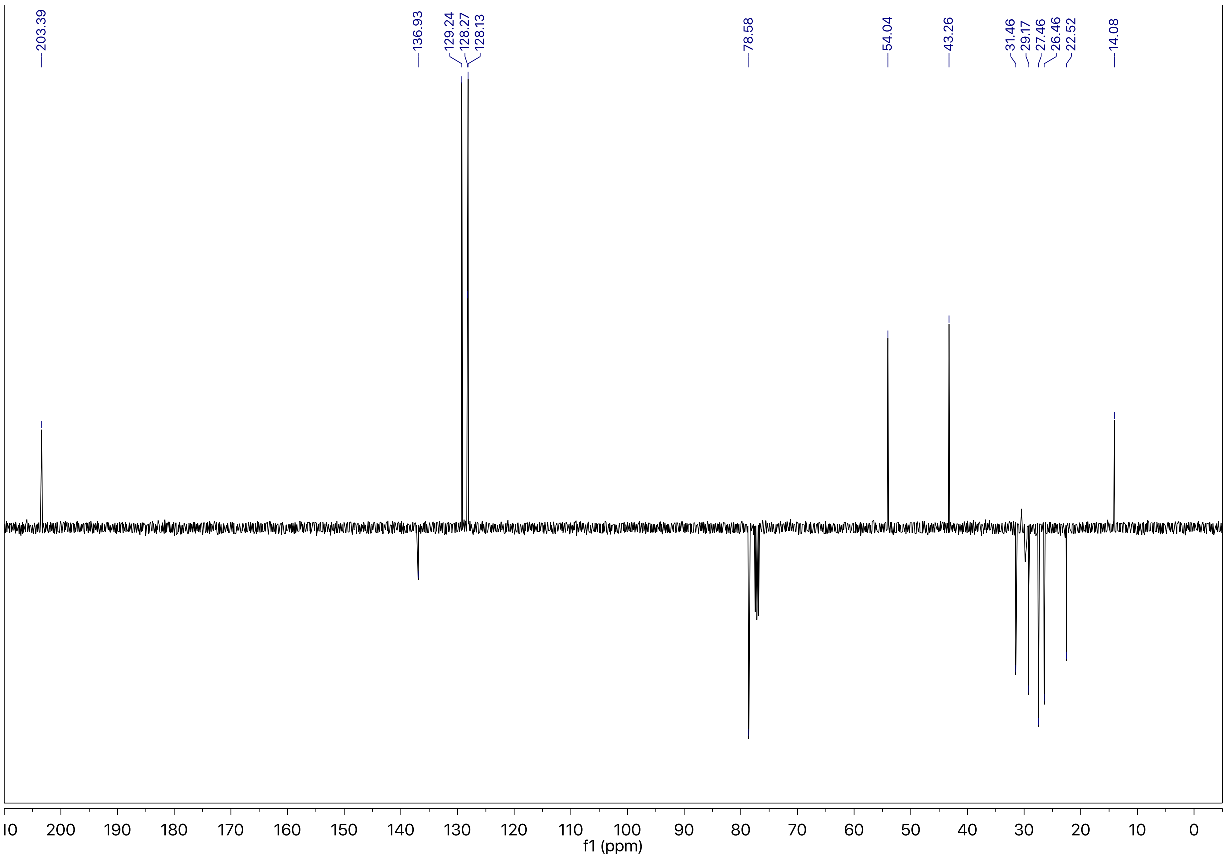

^{13}C NMR of (*R*)-2-[(*S*)-2-nitro-1-phenylethyl]octanal **9e**

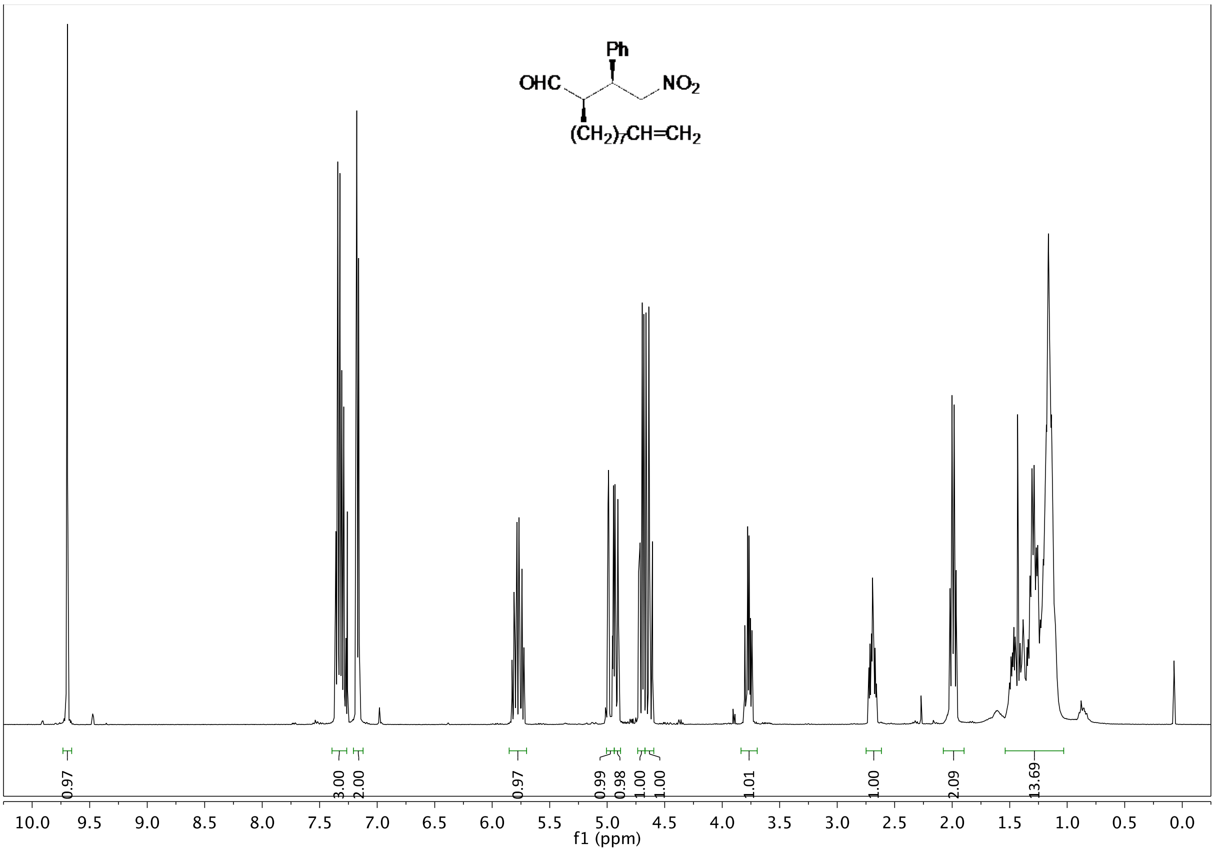

^{1}H NMR of (*R*)-2-[(*S*)-2-nitro-1-phenylethyl]undec-10-enal **9f**

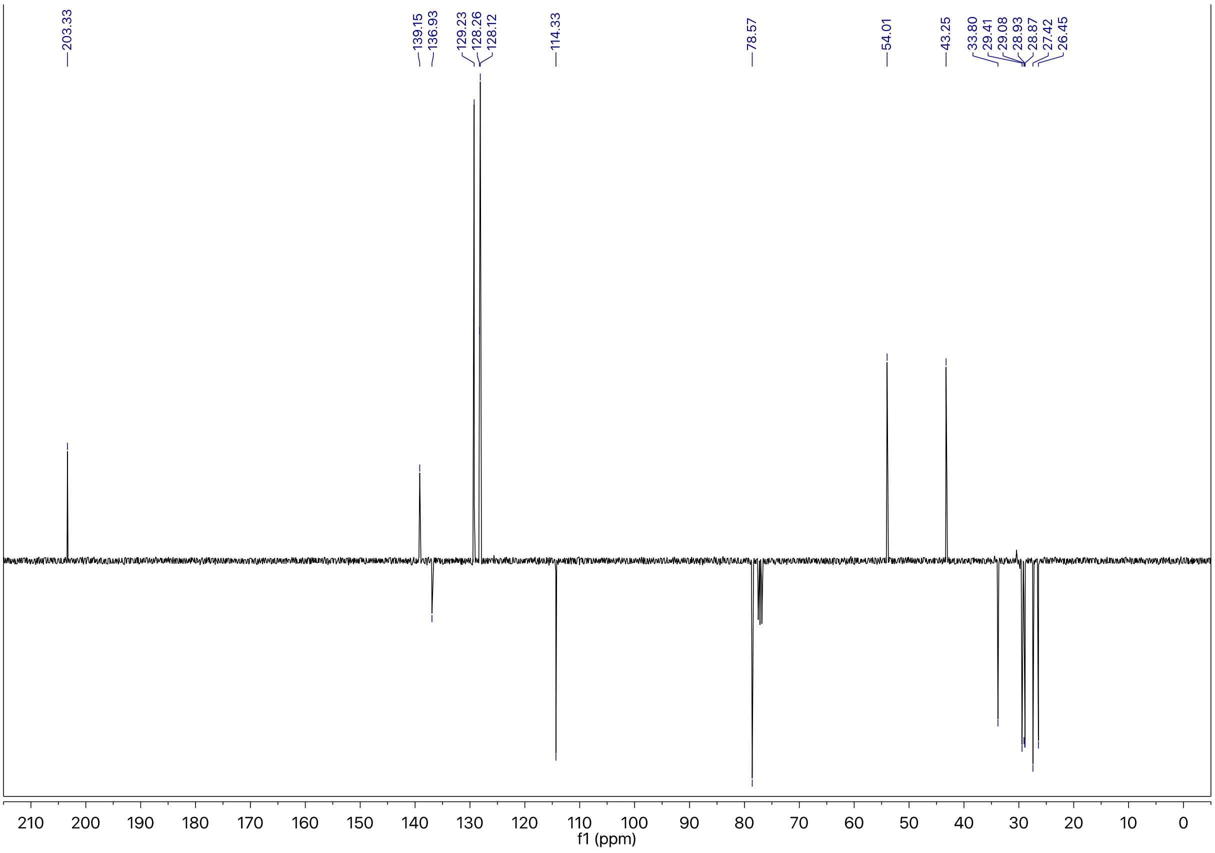

^{13}C NMR of (*R*)-2-[(*S*)-2-nitro-1-phenylethyl]undec-10-enal **9f**

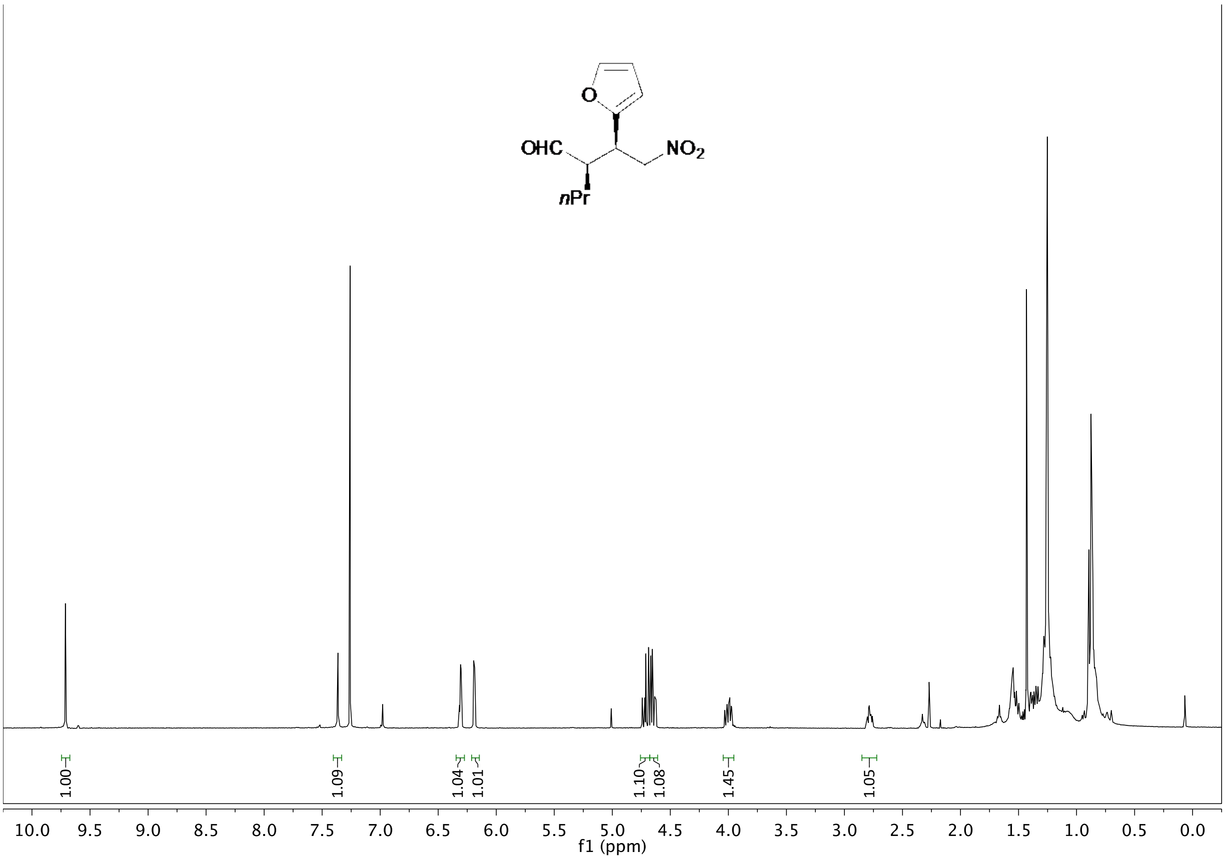

^{1}H NMR of (*R*)-2-[(*R*)-1-(furan-2-yl)-2-nitroethyl]pentanal **9g**

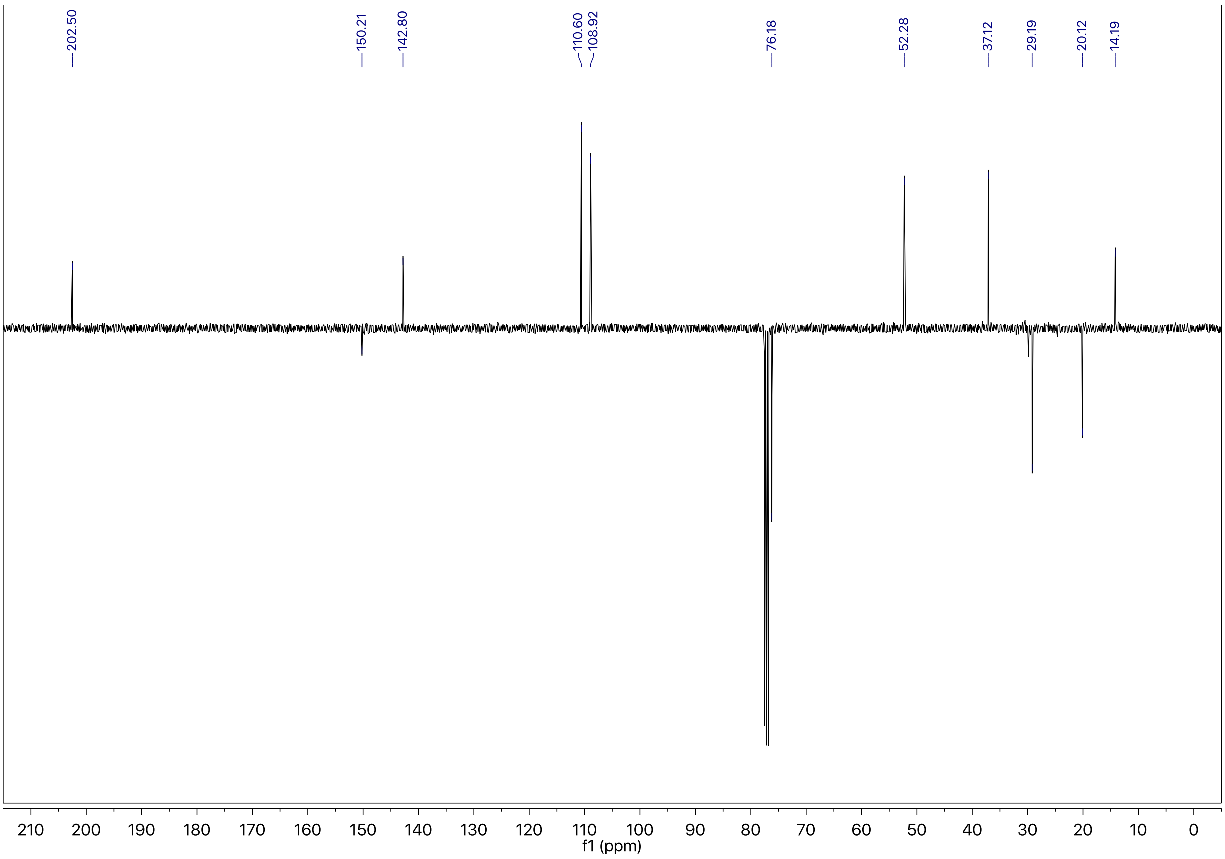

^{13}C NMR of (*R*)-2-[(*R*)-1-(furan-2-yl)-2-nitroethyl]pentanal **9g**

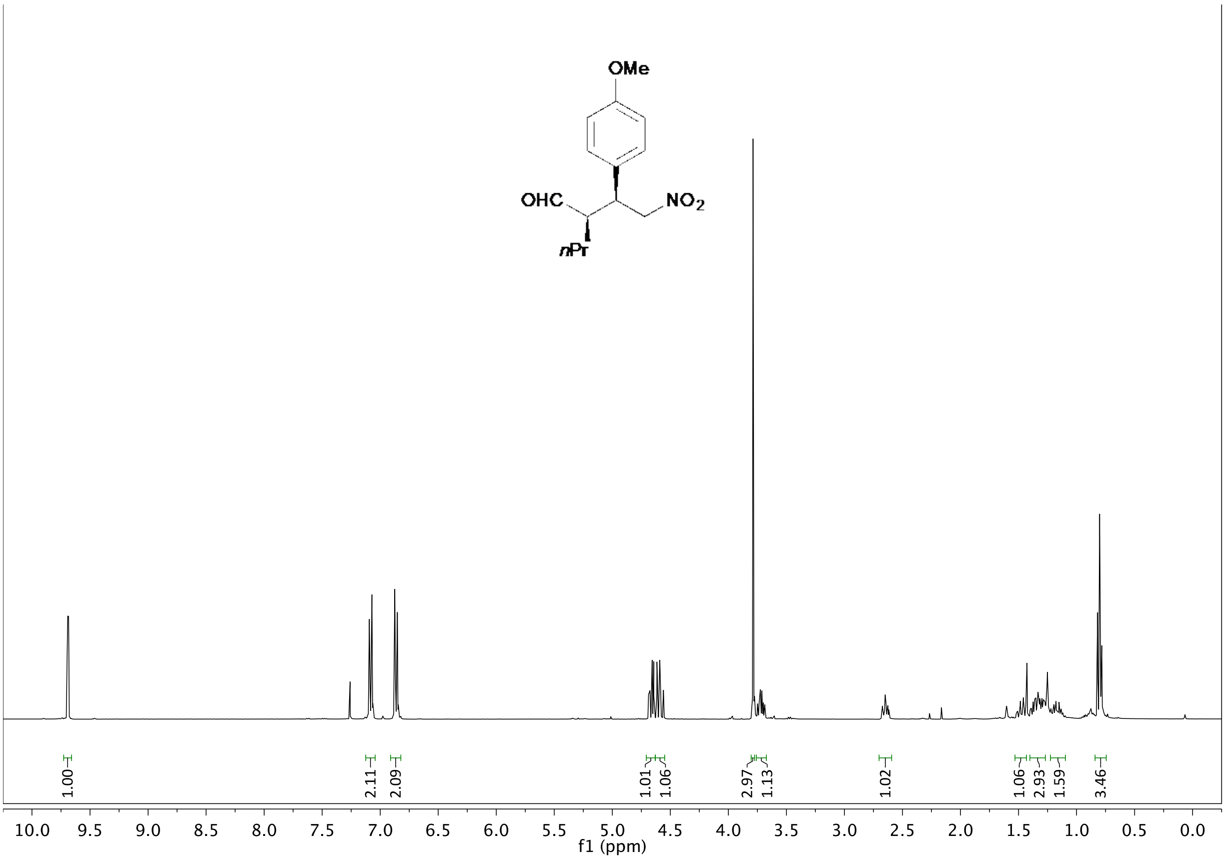

^{1}H NMR of (*R*)-2-[(*S*)-1-(4-methoxyphenyl)-2-nitroethyl]pentanal **9h**

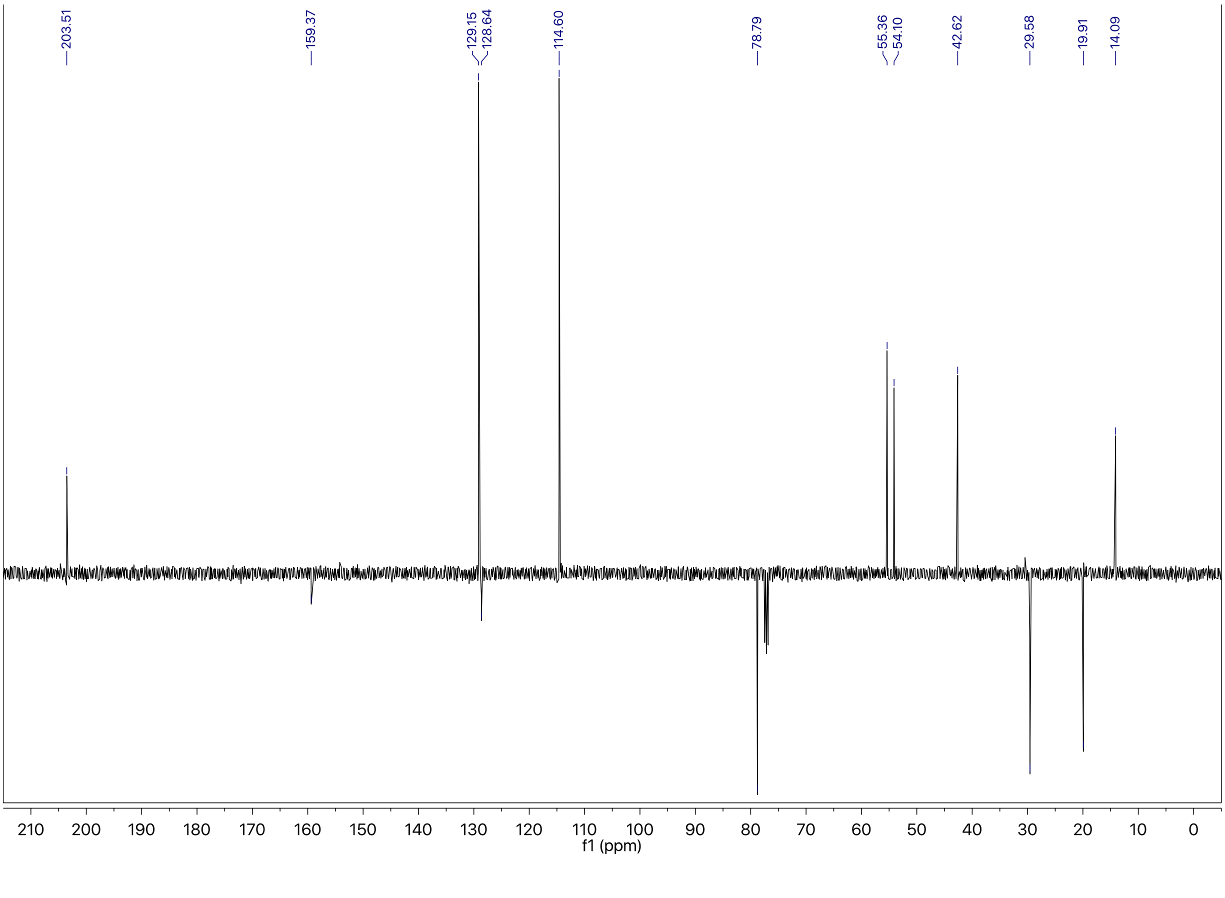

^{13}C NMR of (*R*)-2-[(*S*)-1-(4-methoxyphenyl)-2-nitroethyl]pentanal **9h**

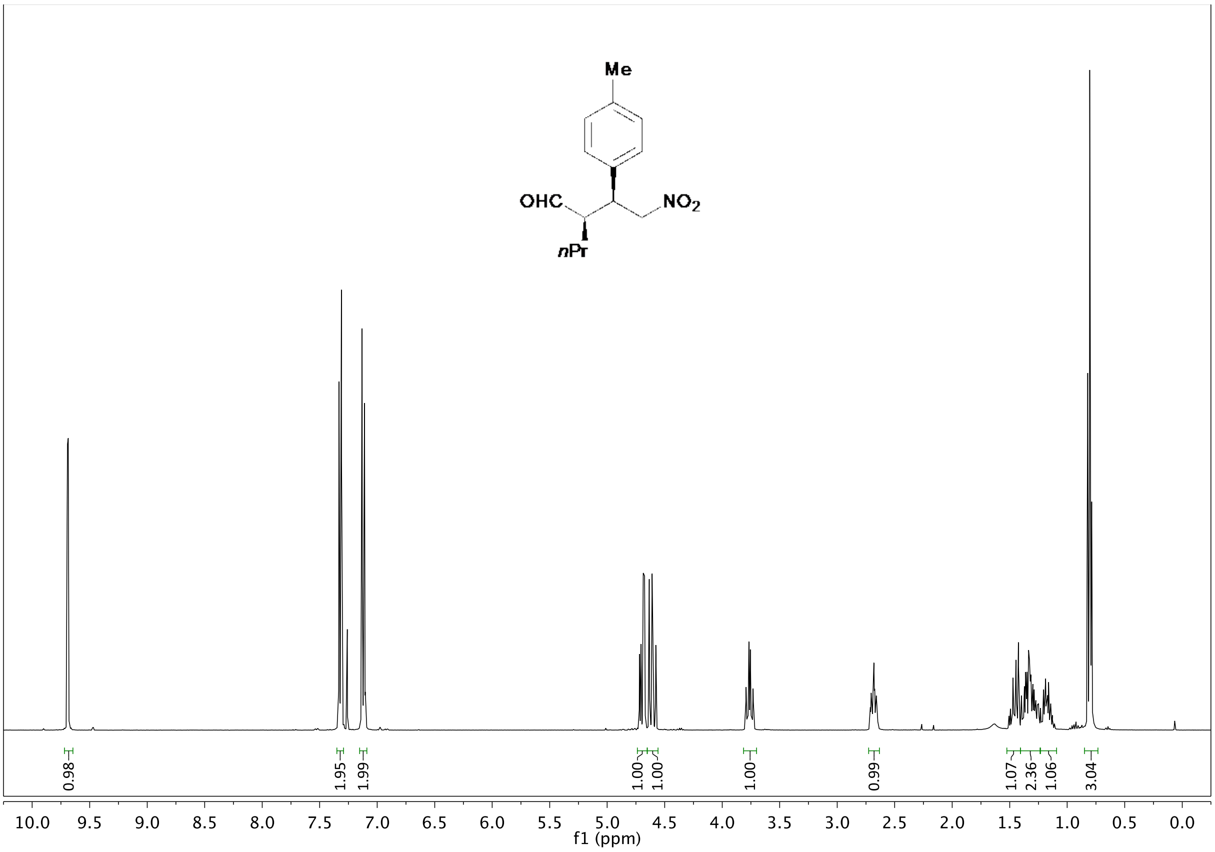

^{1}H NMR of (*R*)-2-[(*S*)-1-(4-methylphenyl)-2-nitroethyl]pentanal **9i**

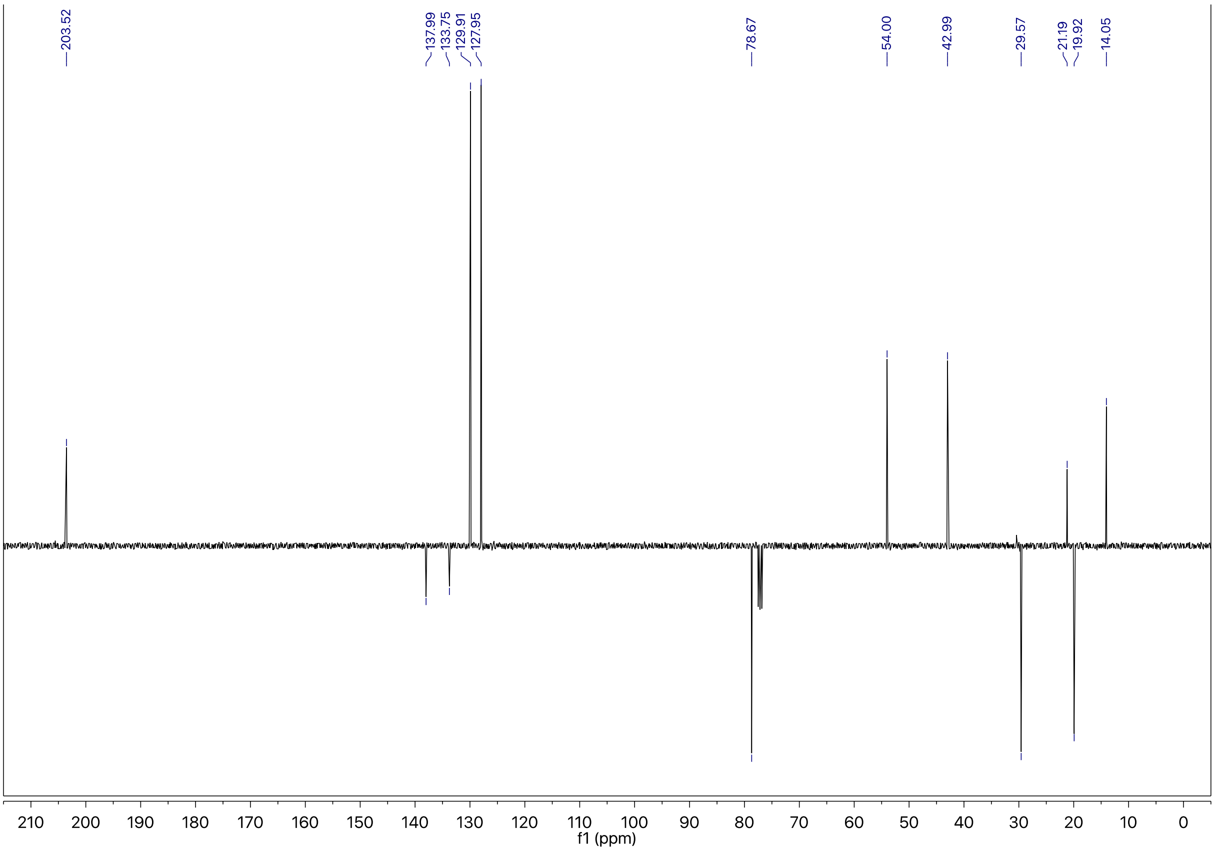

^{13}C NMR of (*R*)-2-[(*S*)-1-(4-methylphenyl)-2-nitroethyl]pentanal **9i**

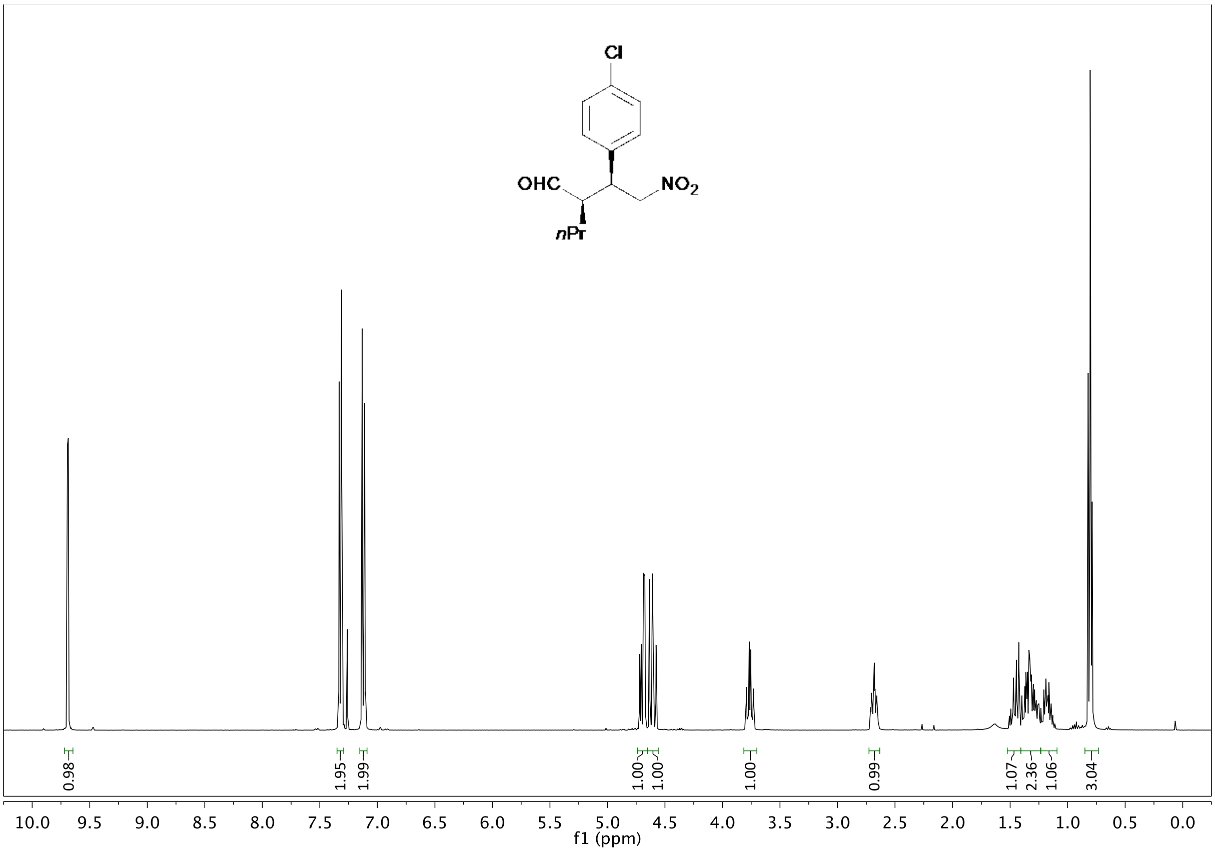

^{1}H NMR of (*R*)-2-[(*S*)-1-(4-chlorophenyl)-2-nitroethyl]pentanal **9j**

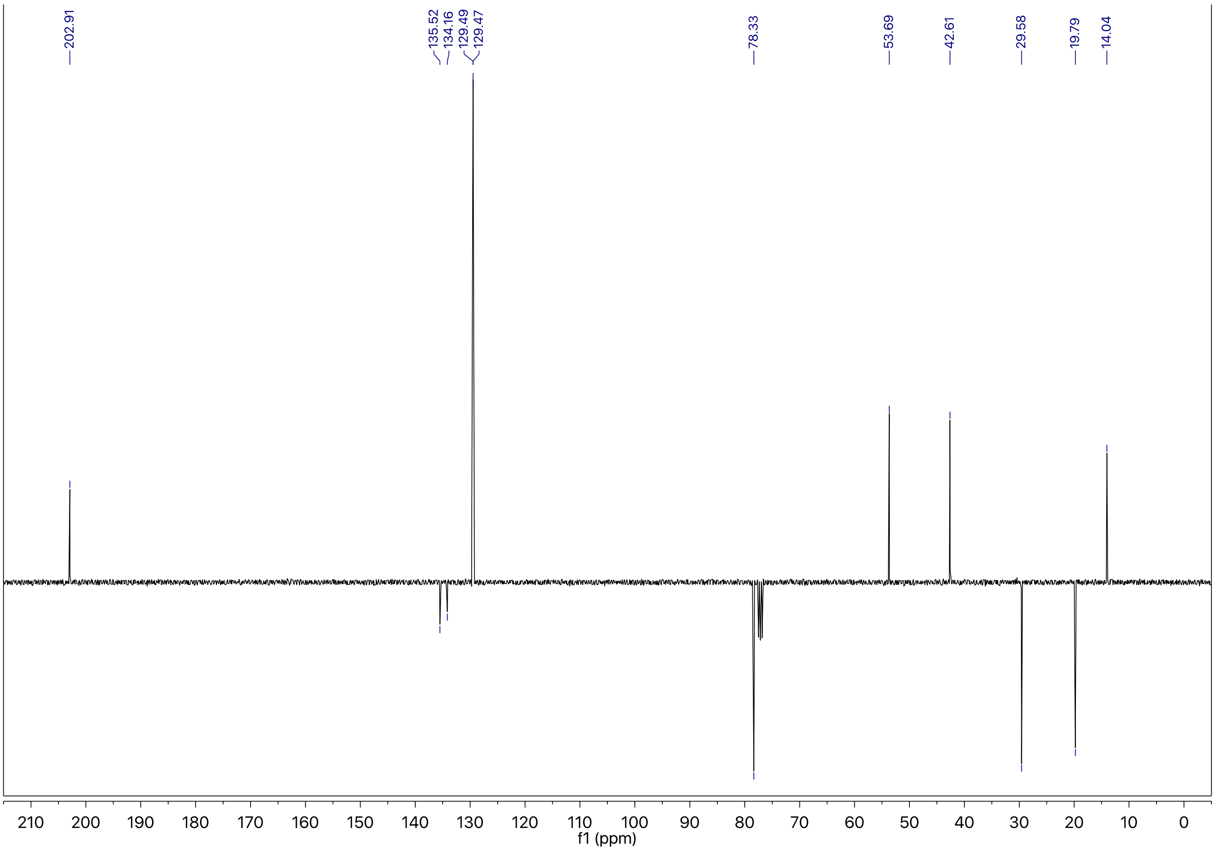

^{13}C NMR of (*R*)-2-[(*S*)-1-(4-chlorophenyl)-2-nitroethyl]pentanal **9j**

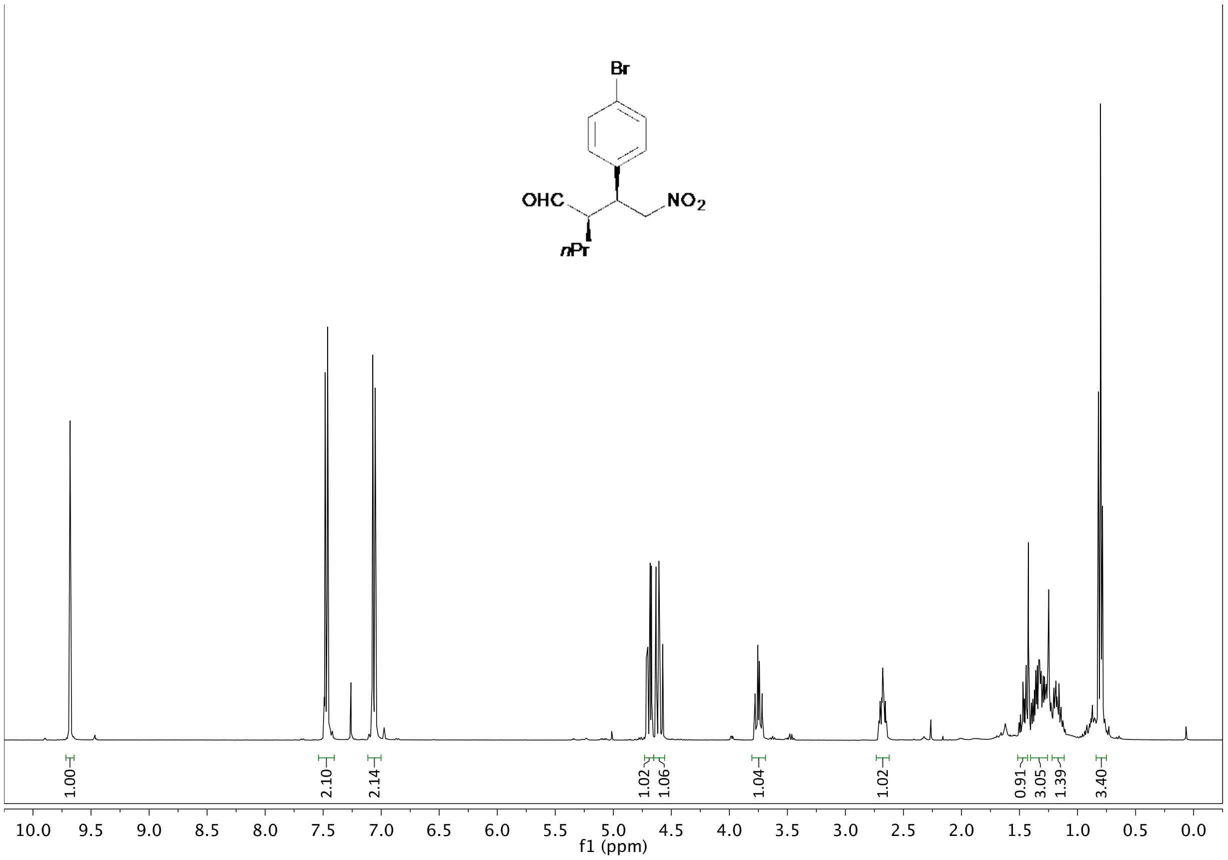

^{1}H NMR of (*R*)-2-[(*S*)-1-(4-bromophenyl)-2-nitroethyl]pentanal **9k**

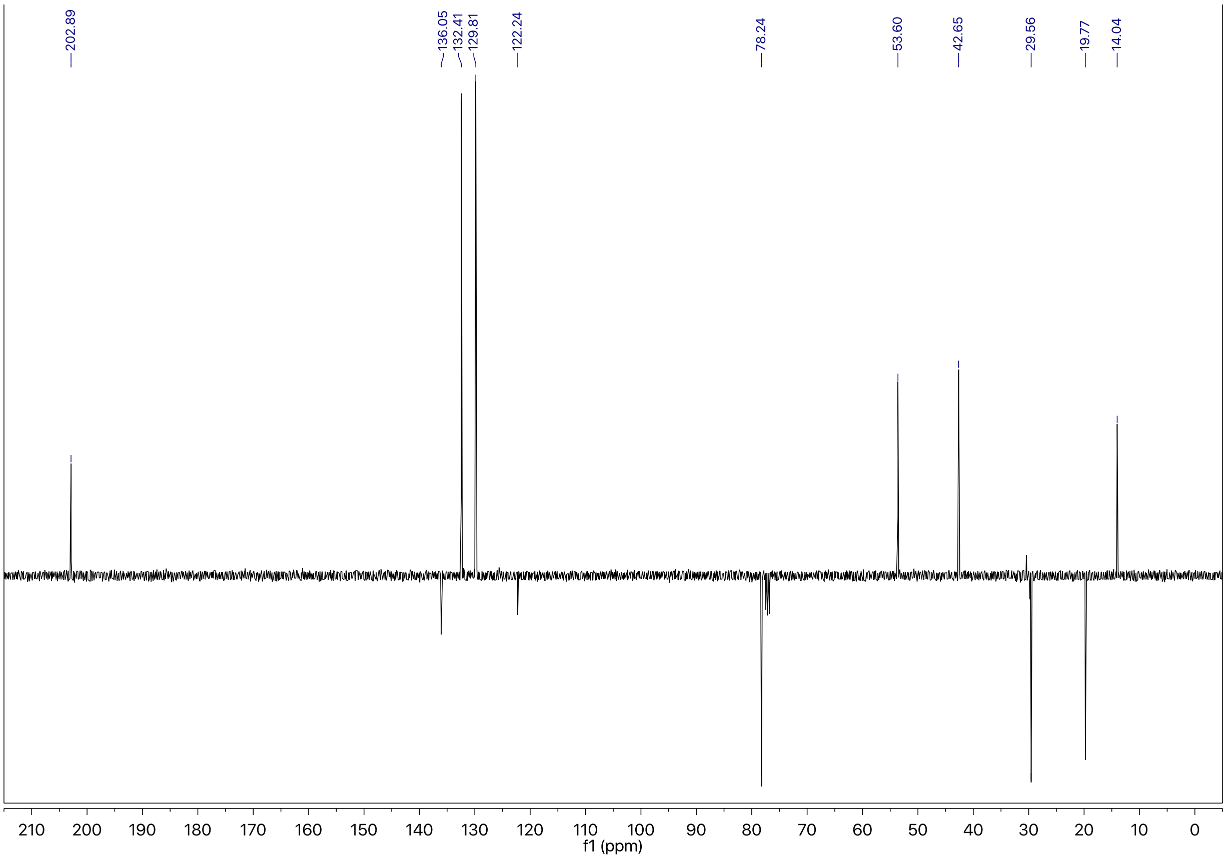

^{13}C NMR of (*R*)-2-[(*S*)-1-(4-bromophenyl)-2-nitroethyl] pentanal **9k**

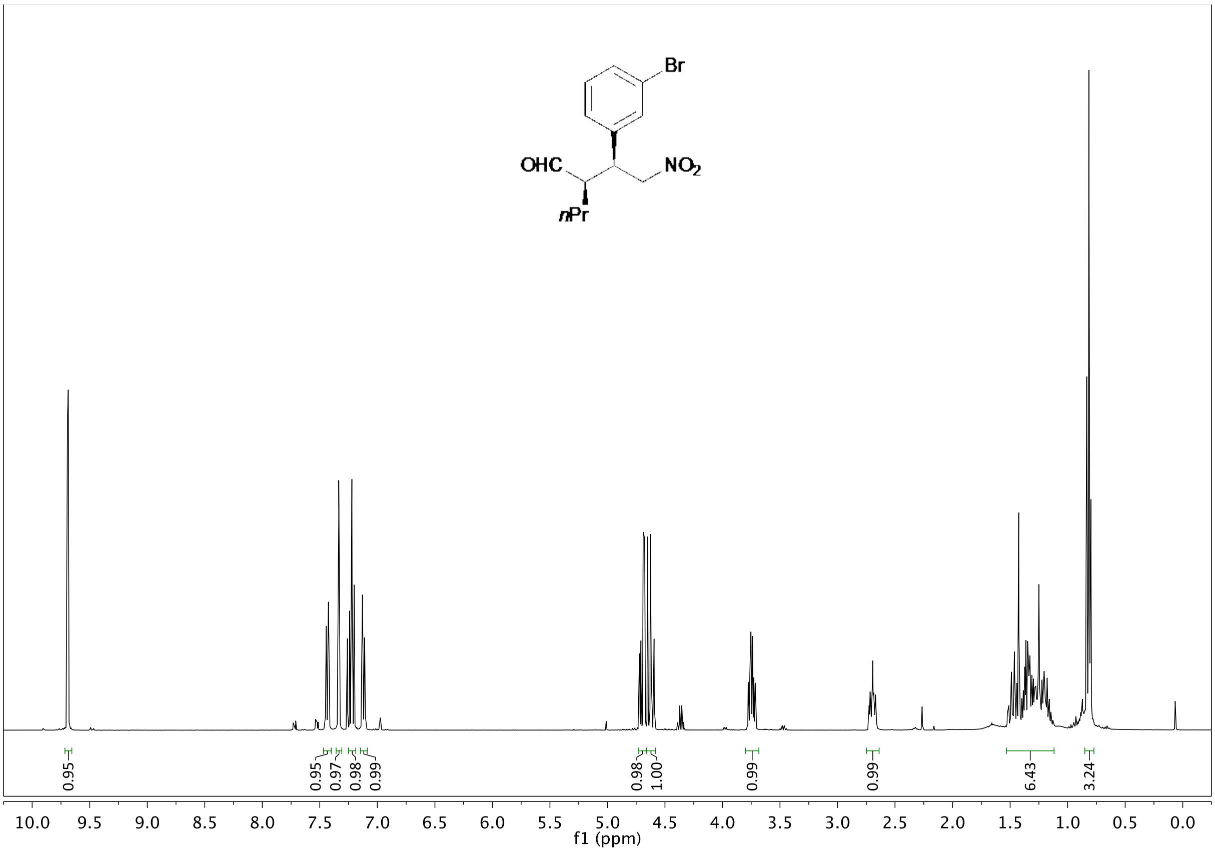

^{1}H NMR of (*R*)-2-[(*S*)-1-(3-bromophenyl)-2-nitroethyl]pentanal **9l**

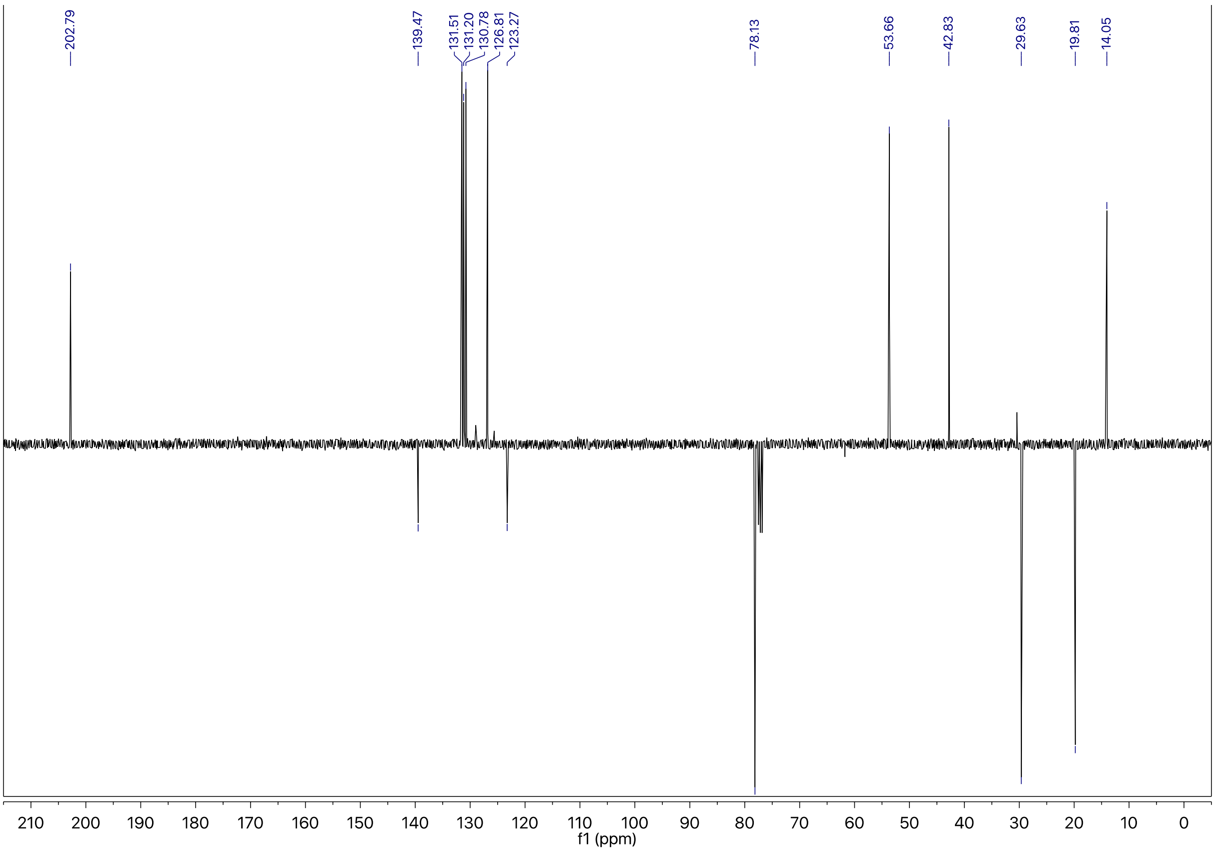

^{13}C NMR of (*R*)-2-[(*S*)-1-(3-bromophenyl)-2-nitroethyl]pentanal **9l**

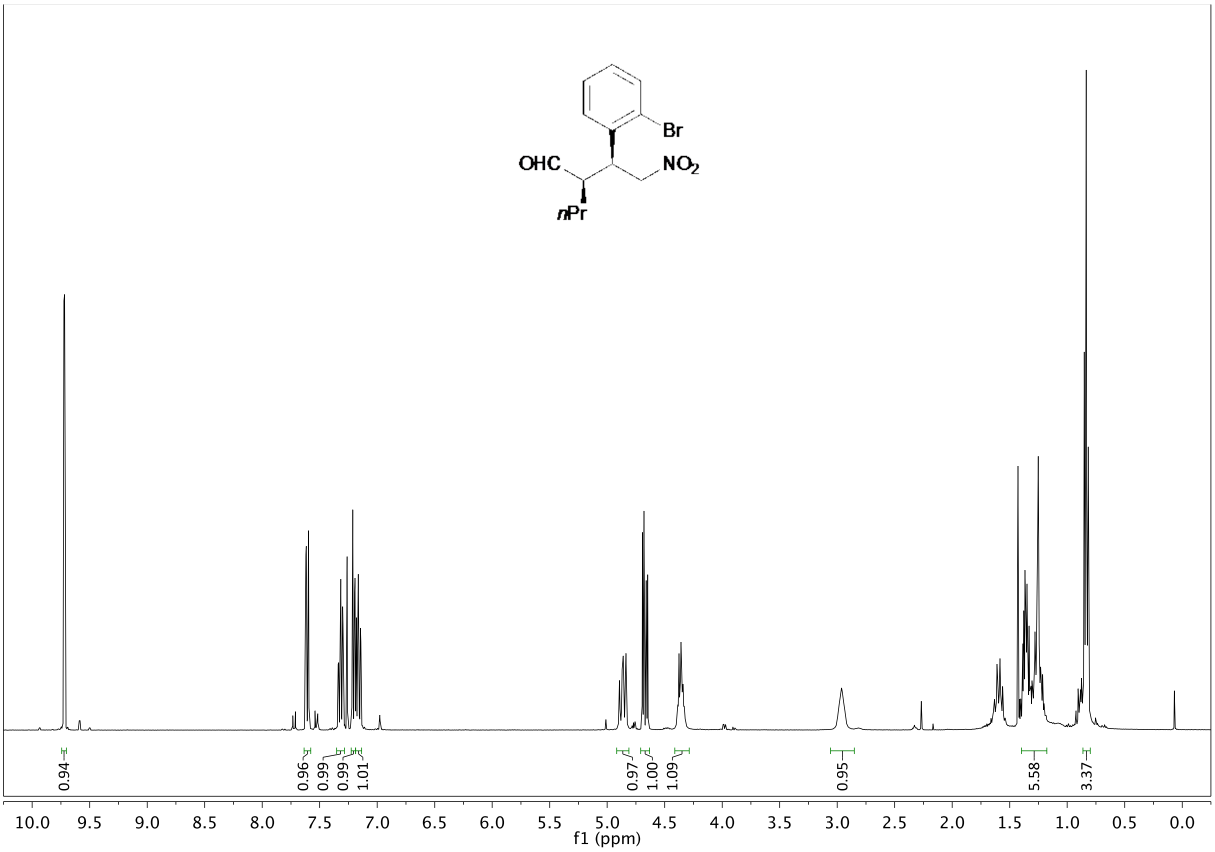

^{1}H NMR of (*R*)-2-[(*S*)-1-(2-bromophenyl)-2-nitroethyl]pentanal **9m**

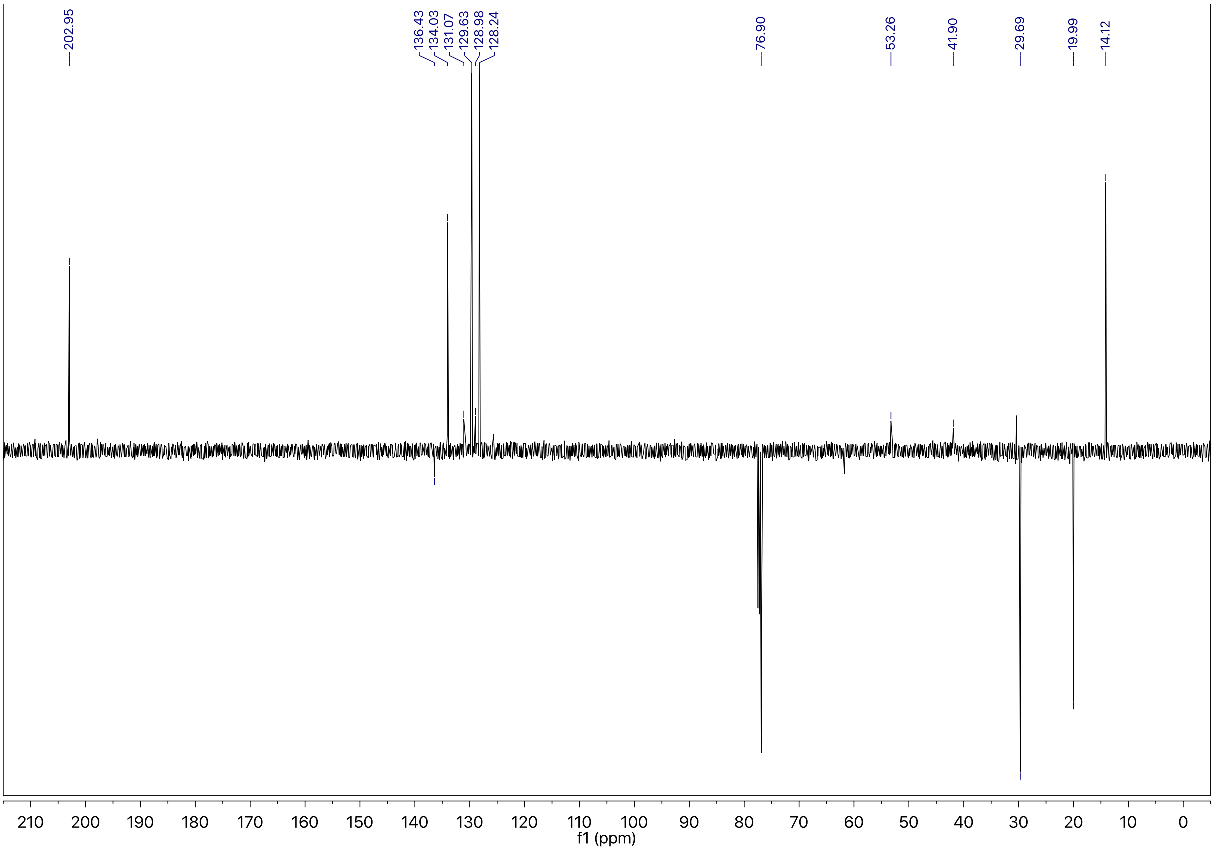

^{13}C NMR of (*R*)-2-[(*S*)-1-(2-bromophenyl)-2-nitroethyl]pentanal **9m**

2-Benzyl-4-nitro-3-phenylbutanal

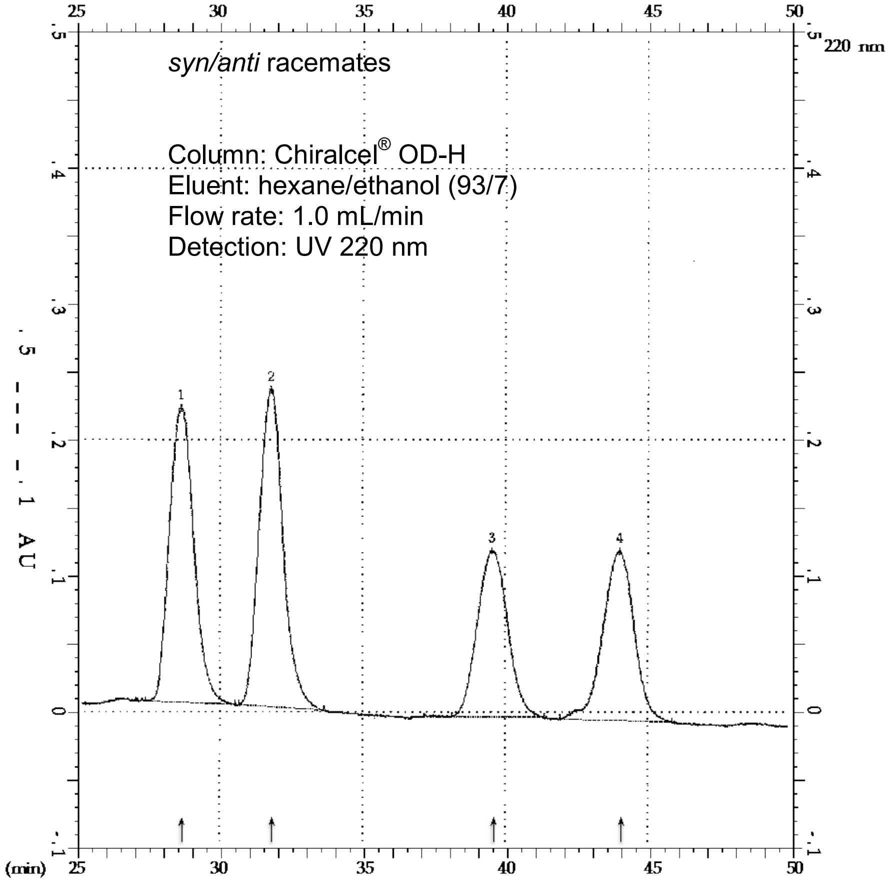

220 nm Report File YBAN2001.DT3

No.	Retention time	Height [AU]	Left time	Right time	Area [AU*min]	Area [%]	Mark
1	28.65	0.2171	27.39	30.53	0.216229	28.654	I
2	31.79	0.2338	30.66	33.66	0.227620	30.163	I
3	39.53	0.1223	38.12	41.35	0.151201	20.036	I
4	44.01	0.1251	41.91	45.84	0.159579	21.147	I

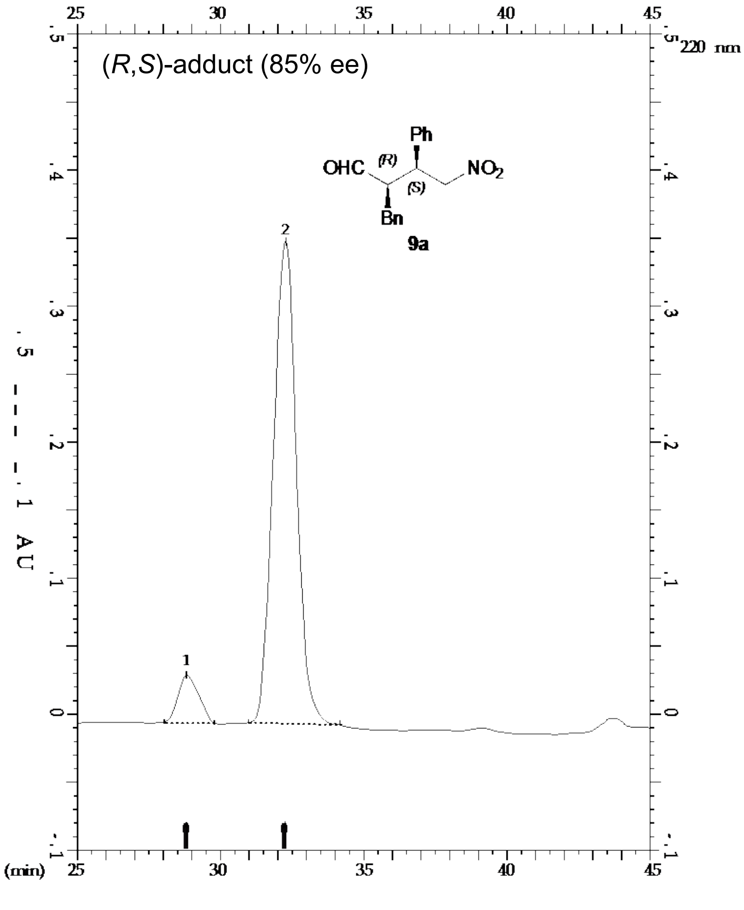

220 nm Report File yban3001.DT3

No.	Retention time	Height [AU]	Left time	Right time	Area [AU*min]	Area [%]	Mark
1	28.74	0.0303	27.93	29.66	0.026068	7.427	I
2	32.14	0.3521	31.01	33.85	0.324902	92.573	I

2-(2-Nitro-1-phenylethyl)pentanal

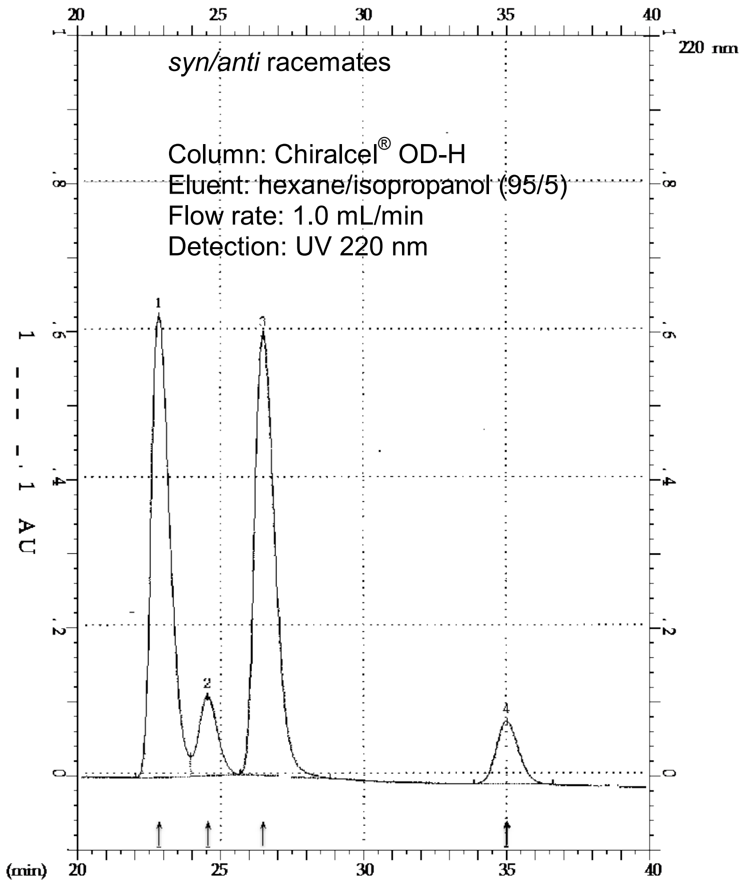

220 nm Report File PUL23001.DT3

No.	Retention time	Height [AU]	Left time	Right time	Area [AU*min]	Area [%]	Mark
1	22.85	0.6223	22.00	23.00	0.477169	42.197	V
2	24.54	0.1069	23.94	25.67	0.082345	7.282	I
3	26.50	0.5952	25.64	28.83	0.492885	43.586	I
4	35.01	0.0847	33.88	36.64	0.078422	6.935	

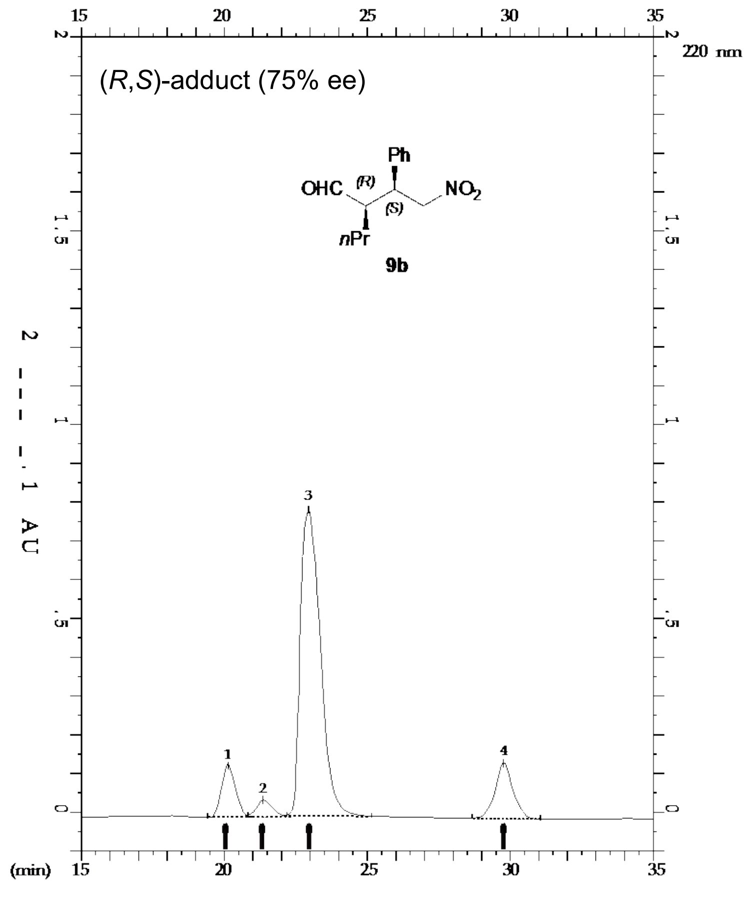

220 nm Report File YBAN2001.DT3

No.	Retention time	Height [AU]	Left time	Right time	Area [AU*min]	Area [%]	Mark
1	20.12	0.1265	19.48	20.86	0.086142	10.609	V
2	21.40	0.0402	20.86	22.27	0.028260	3.480	I
3	23.00	0.7790	22.23	25.19	0.607107	74.771	I
4	29.74	0.1286	28.71	31.10	0.090446	11.139	

(2*R*,3*S*)-2-Ethyl-4-nitro-3-phenylbutanal

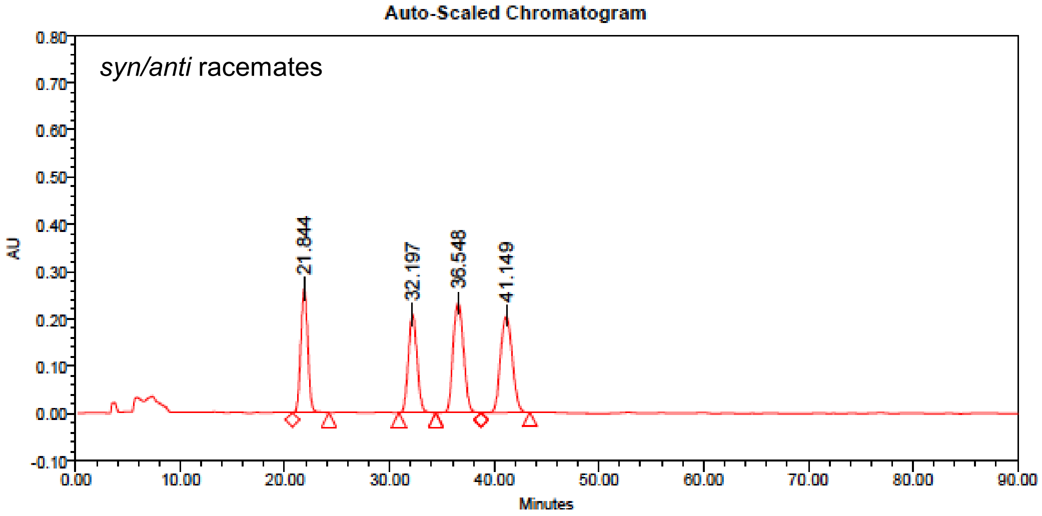

Processed Channel:

	Peak Name	Retention Time (min)	Area	% Area	Height
1	Peak1	21.844	12369715	21.76	261765
2	Peak2	32.197	12309531	21.65	207955
3	Peak3	36.548	16133522	28.38	231364
4	Peak4	41.149	16043287	28.22	203876

Column: Chiralpak® IC
Eluent: hexane/isopropanol (90/10)
Flow rate: 1 mL/min
Detection: UV 220 nm

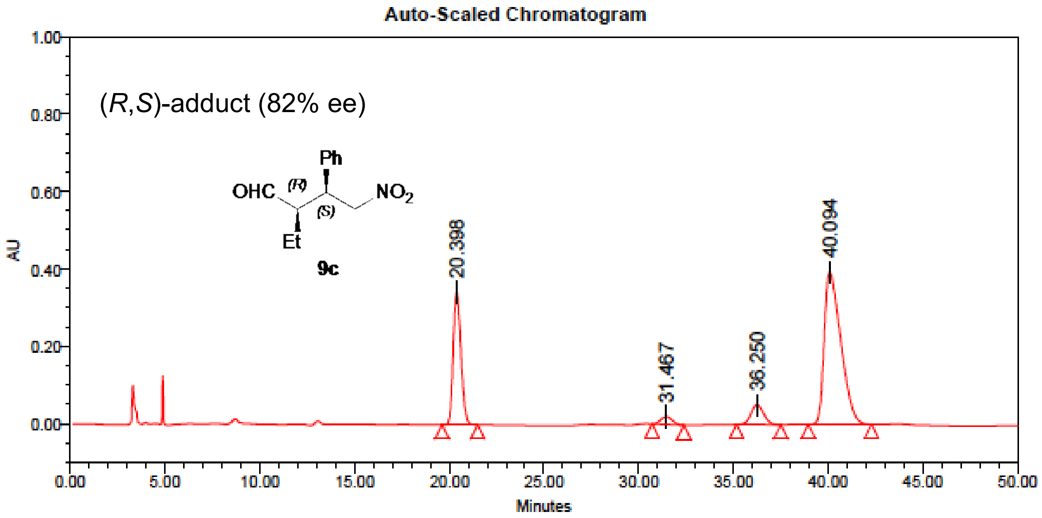

Processed Channel:

	Peak Name	Retention Time (min)	Area	% Area	Height
1	Peak1	20.398	9707481	26.40	341729
2	Peak2	31.467	830263	2.26	20123
3	Peak3	36.250	2382084	6.48	51033
4	Peak4	40.094	23850533	64.86	392498

(2*R*,3*S*)-2-Methyl-4-nitro-3-phenylbutanal

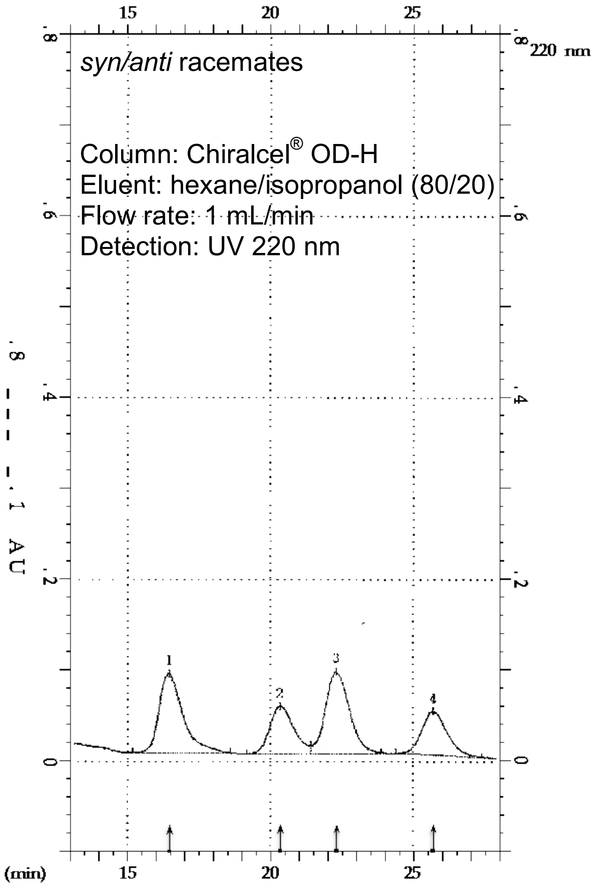

220 nm Report File PUL23001.DT3

No.	Retention time	Height [AU]	Left time	Right time	Area [AU*min]	Area [%]	Mark
1	16.48	0.0871	15.18	18.60	0.085382	31.081	I
2	20.33	0.0523	19.17	21.43	0.052145	18.982	
3	22.31	0.0901	21.43	23.88	0.086726	31.570	V
4	25.71	0.0479	24.40	27.39	0.050458	18.368	I

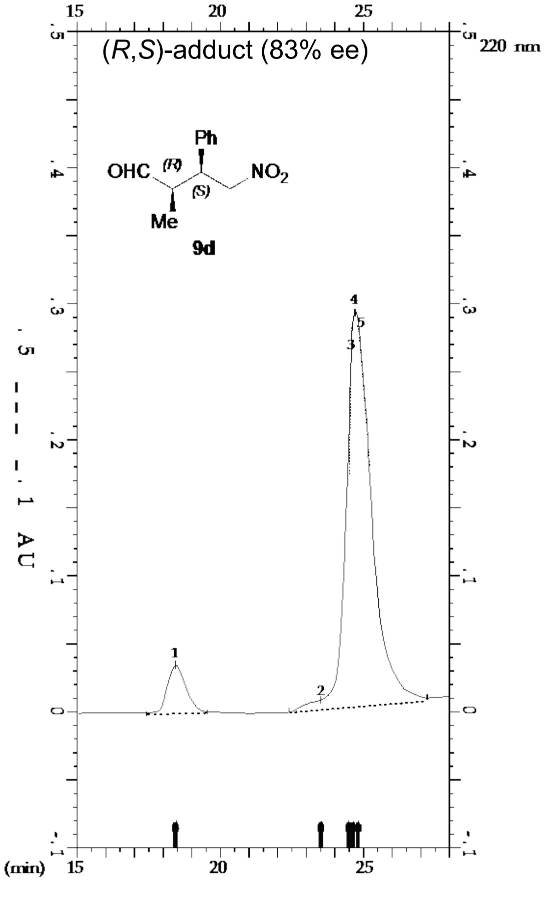

220 nm Report File yban4001.DT3

No.	Retention time	Height [AU]	Left time	Right time	Area [AU*min]	Area [%]	Mark
1	18.43	0.0384	17.17	19.54	0.029683	8.398	I
2	23.50	0.0074	22.43	23.74	0.005164	1.461	
3	24.45	0.0322	24.18	24.65	0.008288	2.345	TB
4	24.68	0.2971	23.37	27.20	0.302806	85.672	V
5	24.90	0.0233	24.71	25.31	0.007507	2.124	TA

2-(2-Nitro-1-phenylethyl)octanal

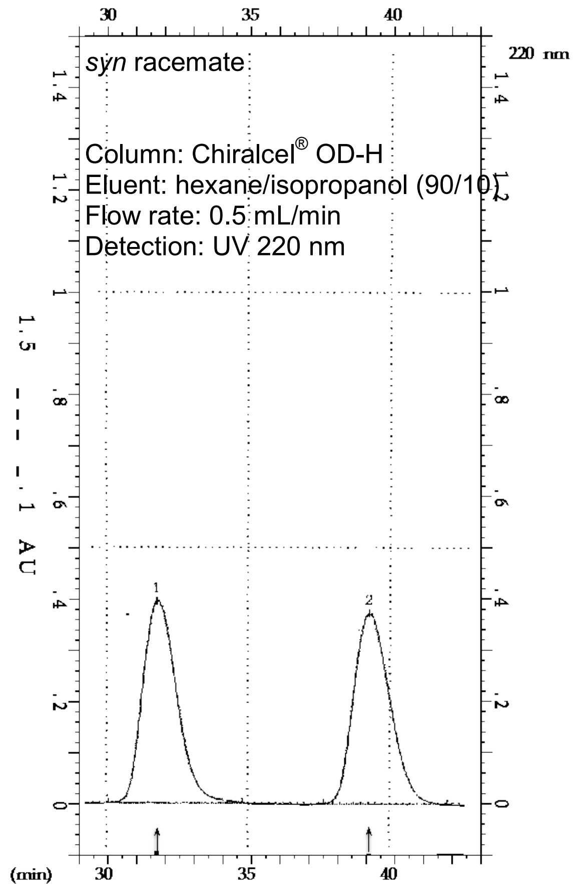

220 nm Report File yban3001.DT3

No.	Retention time	Height [AU]	Left time	Right time	Area [AU*min]	Area [%]	Mark
1	31.79	0.3962	30.06	34.82	0.556779	50.120	I
2	39.26	0.3750	37.66	42.06	0.554123	49.880	I

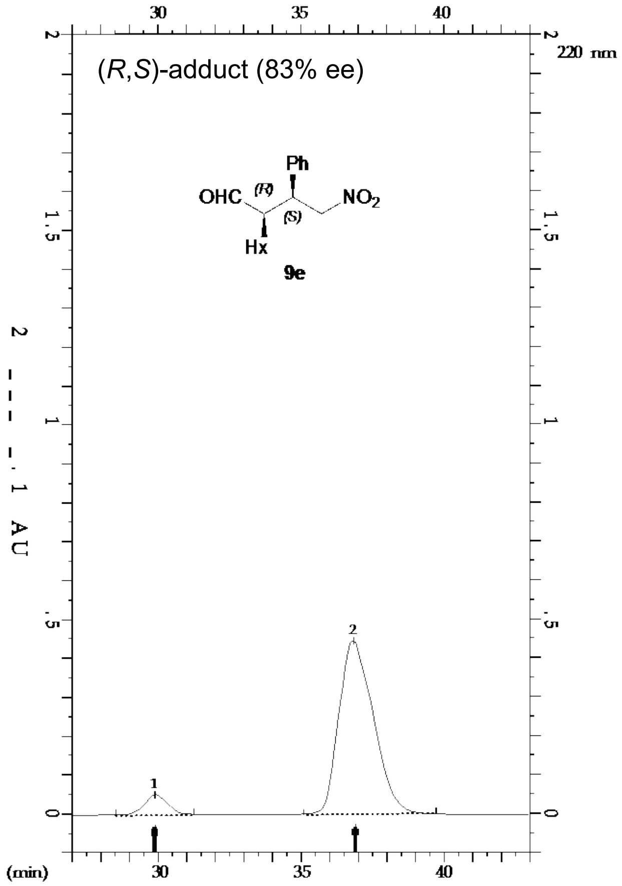

220 nm Report File YBAN3001.DT3

No.	Retention time	Height [AU]	Left time	Right time	Area [AU*min]	Area [%]	Mark
1	29.95	0.0498	28.56	31.39	0.056831	8.384	I
2	36.98	0.4443	35.07	39.82	0.621016	91.616	I

2-(2-Nitro-1-phenylethyl)undec-10-enal

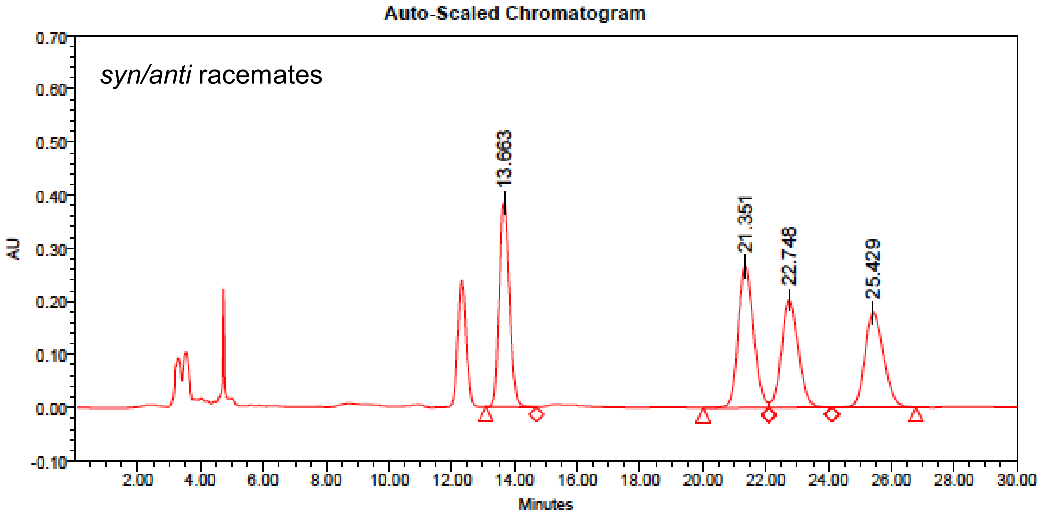

Processed Channel:

	Peak Name	Retention Time (min)	Area	% Area	Height
1	Peak1	13.663	8729994	26.67	384646
2	Peak2	21.351	9098892	27.80	266172
3	Peak3	22.748	7543260	23.04	202115
4	Peak4	25.429	7362156	22.49	177689

Column: Chiralpak® IC
Eluent: hexane/isopropanol (90/10)
Flow rate: 1 mL/min
Detection: UV 220 nm

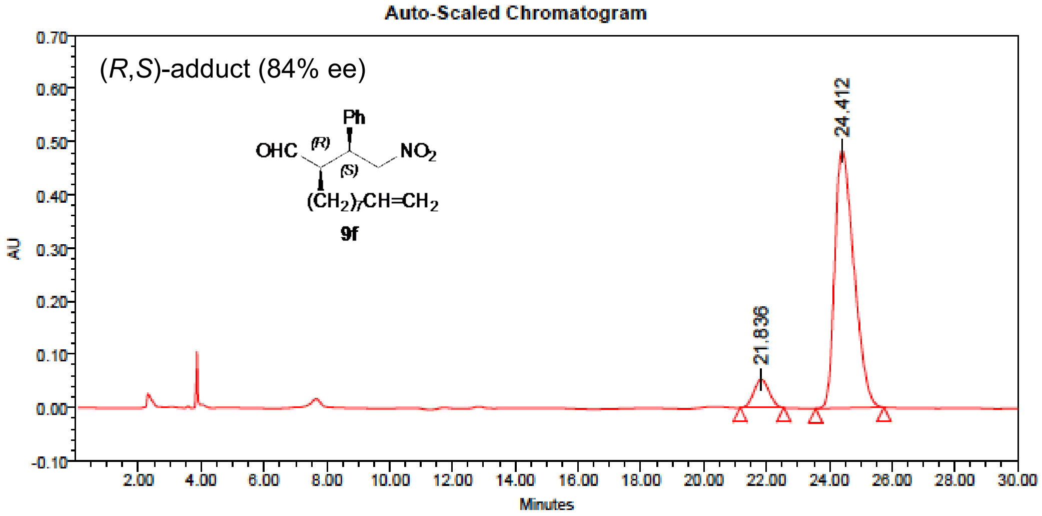

Processed Channel:

	Peak Name	Retention Time (min)	Area	% Area	Height
1	Peak1	21.836	1821301	8.14	53429
2	Peak2	24.412	20557355	91.86	484042

2-[1-(Furan-2-yl)-2-nitroethyl]pentanal

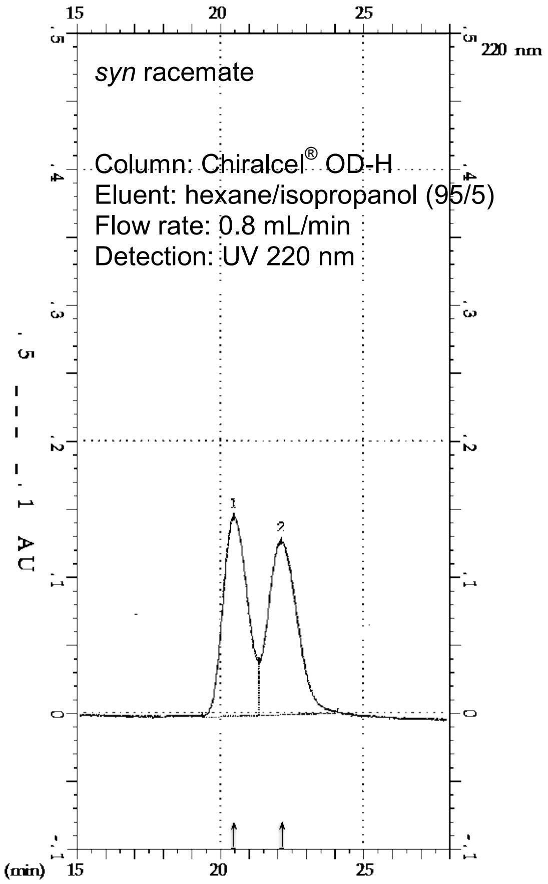

Report File PULK2001.DT3

220 nm

No.	Retention time	Height [AU]	Left time	Right time	Area [AU*min]	Area [%]	Mark
1	20.48	0.1467	19.35	21.36	0.146410	49.431	
2	22.14	0.1280	21.36	24.11	0.149779	50.569	V

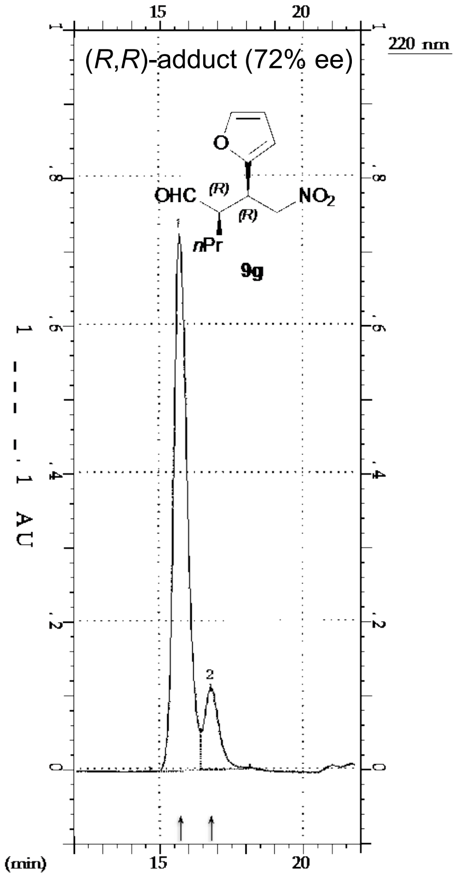

Report File yban2001.DT3

220 nm

No.	Retention time	Height [AU]	Left time	Right time	Area [AU*min]	Area [%]	Mark
1	15.72	0.7206	14.67	16.47	0.405745	86.132	
2	16.80	0.1103	16.47	18.16	0.065331	13.868	V

2-[1-(4-Methoxyphenyl)-2-nitroethyl]pentanal

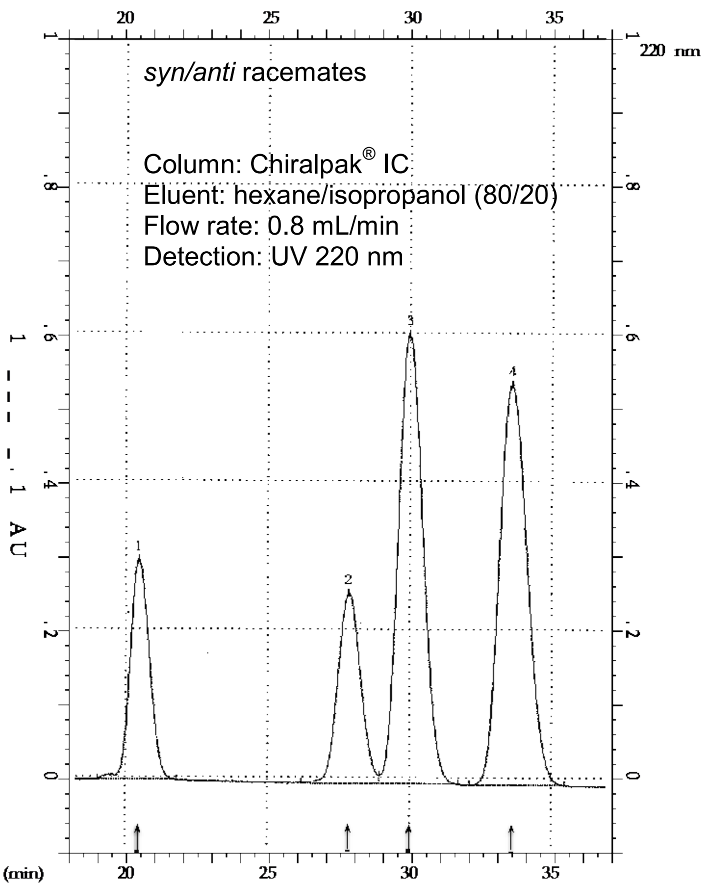

220 nm Report File YBAN2001.DT3

No.	Retention time	Height [AU]	Left time	Right time	Area [AU*min]	Area [%]	Mark
1	20.46	0.2972	19.00	21.82	0.237271	14.014	I
2	27.83	0.2570	26.19	28.93	0.237125	14.005	
3	29.99	0.6070	28.93	31.70	0.609378	35.992	V
4	33.60	0.5403	32.12	35.47	0.609339	35.989	I

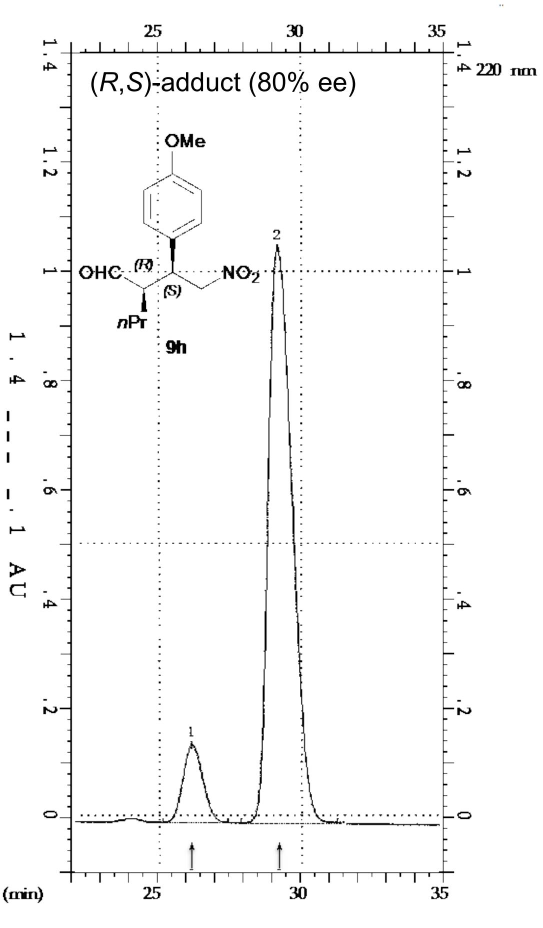

220 nm Report File YBAN1001.DT3

No.	Retention time	Height [AU]	Left time	Right time	Area [AU*min]	Area [%]	Mark
1	26.13	0.1432	25.13	27.42	0.116853	10.237	I
2	29.17	1.0575	27.87	31.26	0.024589	89.763	I

2-[1-(4-Methylphenyl)-2-nitroethyl]pentanal

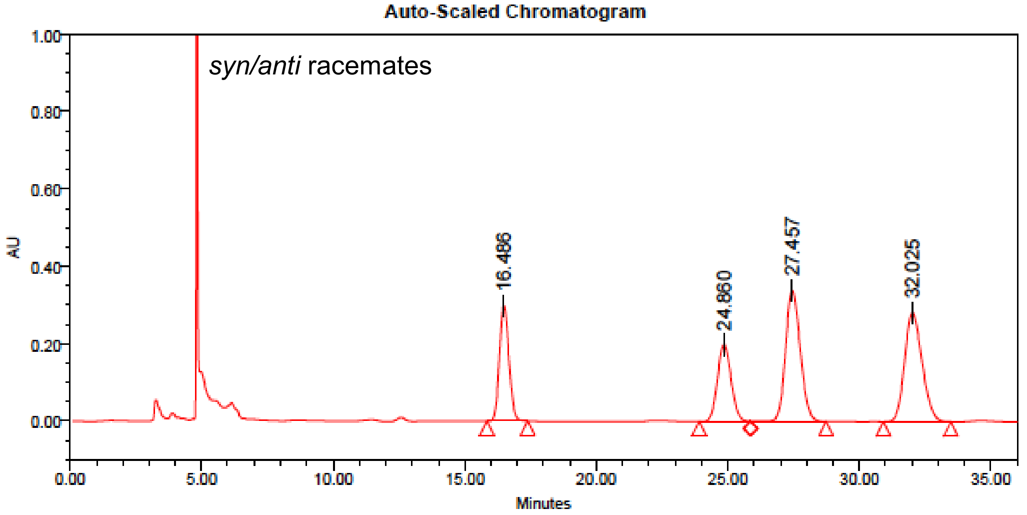

Processed Channel:

	Peak Name	Retention Time (min)	Area	% Area	Height
1	Peak1	16.486	7563572	18.43	298126
2	Peak2	24.860	7197350	17.53	199174
3	Peak3	27.457	13238987	32.25	338499
4	Peak4	32.025	13049130	31.79	281732

Column: Chiralpak® IC
Eluent: hexane/isopropanol (90/10)
Flow rate: 1 mL/min
Detection: UV 220 nm

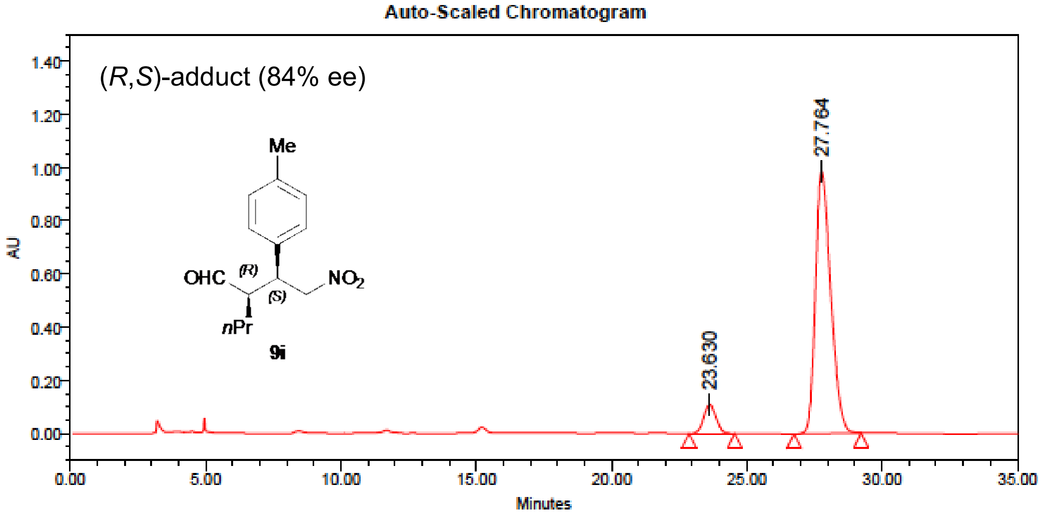

Processed Channel:

	Peak Name	Retention Time (min)	Area	% Area	Height
1	Peak1	23.630	3452856	8.20	109017
2	Peak2	27.764	38677823	91.80	985229

2-[1-(4-Chlorophenyl)-2-nitroethyl]pentanal

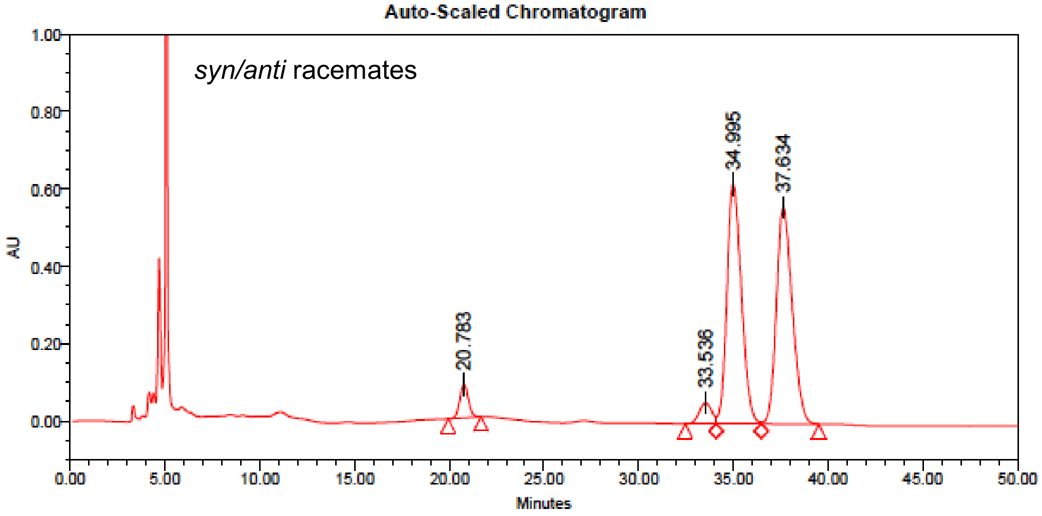

Processed Channel:

	Peak Name	Retention Time (min)	Area	% Area	Height
1	Peak1	20.783	2697137	3.80	85793
2	Peak2	33.536	2521514	3.55	54810
3	Peak3	34.995	33026303	46.55	619620
4	Peak4	37.634	32704407	46.10	560072

Column: Chiralpak® IC
Eluent: hexane/isopropanol (90/10)
Flow rate: 1 mL/min
Detection: UV 220 nm

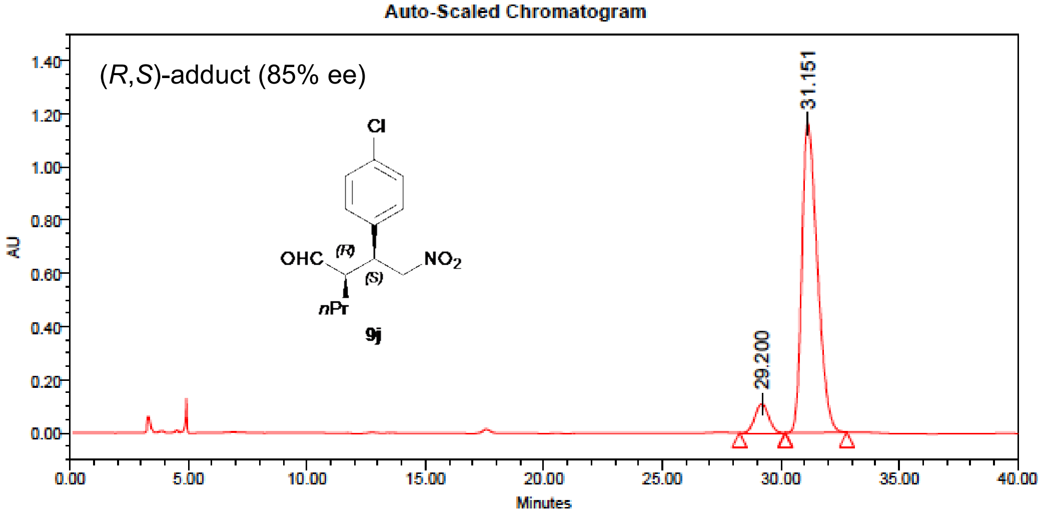

Processed Channel:

	Peak Name	Retention Time (min)	Area	% Area	Height
1	Peak1	29.200	4415849	7.67	108885
2	Peak2	31.151	53137678	92.33	1163159

2-[1-(4-Bromophenyl)-2-nitroethyl]pentanal

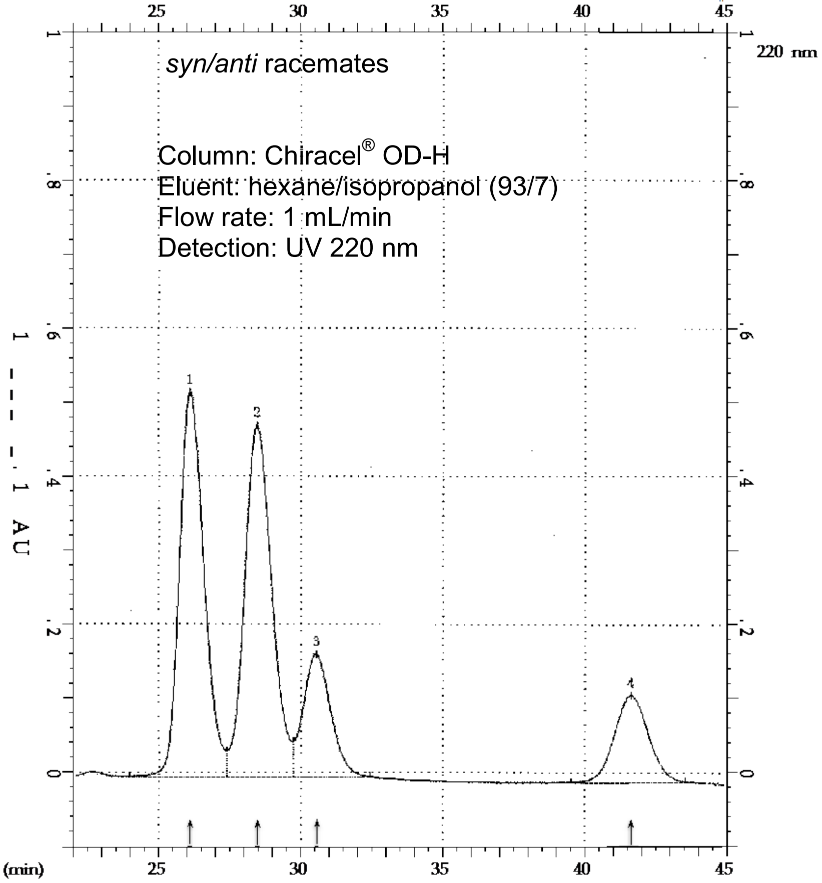

220 nm Report File PUL12001.DT3

No.	Retention time	Height [AU]	Left time	Right time	Area [AU*min]	Area [%]	Mark
1	26.10	0.5190	23.96	27.39	0.521830	37.388	
2	28.46	0.4748	27.39	29.72	0.524397	37.572	V
3	30.53	0.1653	29.72	32.41	0.185941	13.322	V
4	41.62	0.1168	39.52	43.55	0.163547	11.718	I

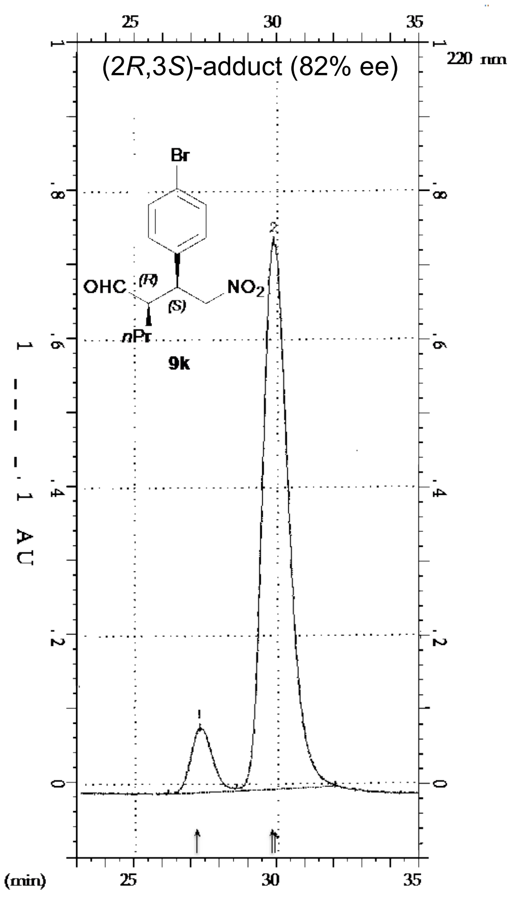

220 nm Report File YBAN4001.DT3

No.	Retention time	Height [AU]	Left time	Right time	Area [AU*min]	Area [%]	Mark
1	27.28	0.0877	26.16	28.54	0.075943	8.989	
2	29.87	0.7402	28.54	31.98	0.768884	91.011	V

2-[1-(3-Bromophenyl)-2-nitroethyl]pentanal

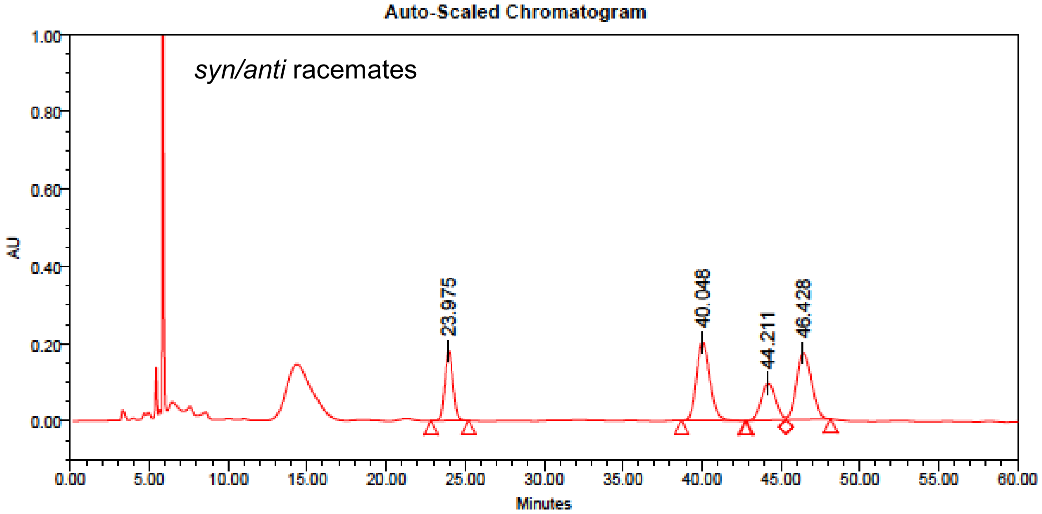

Processed Channel:

	Peak Name	Retention Time (min)	Area	% Area	Height
1	Peak1 2487Channel 1	23.975	6443861	17.93	179574
2	Peak2 2487Channel 1	40.048	11977582	33.33	202334
3	Peak3 2487Channel 1	44.211	6065578	16.88	95254
4	Peak4 2487Channel 1	46.428	11453171	31.87	171632

Column: Chiralpak® IC
Eluent: hexane/isopropanol (95/5)
Flow rate: 1 mL/min
Detection: UV 220 nm

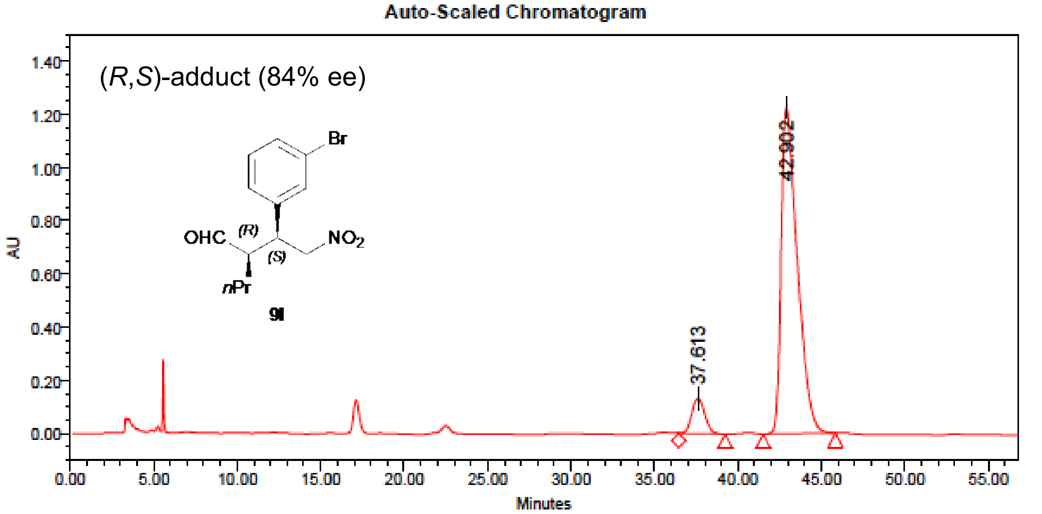

Processed Channel:

	Peak Name	Retention Time (min)	Area	% Area	Height
1	Peak1	37.613	7221985	8.07	133117
2	Peak2	42.902	82305323	91.93	1225475

2-[1-(2-Bromophenyl)-2-nitroethyl]pentanal

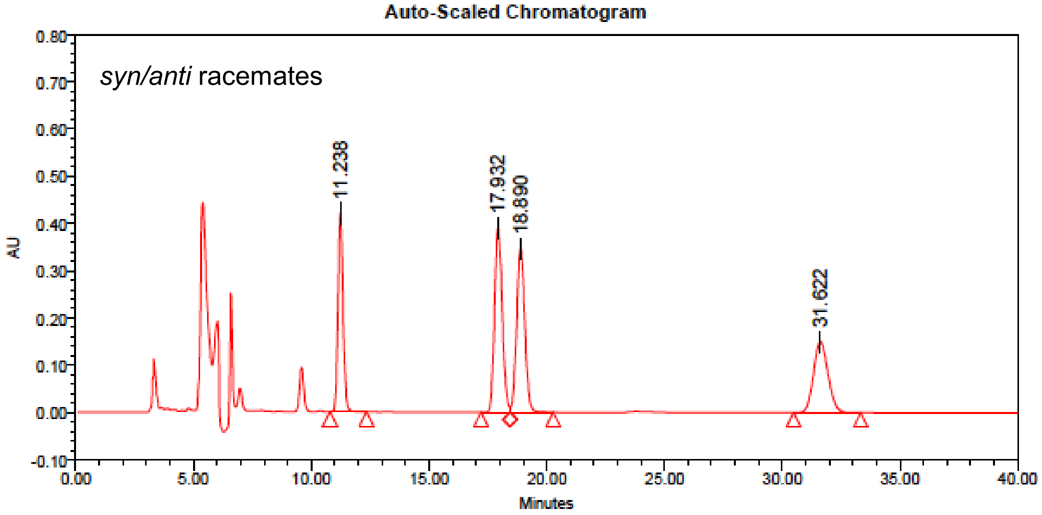

Processed Channel:

	Peak Name	Retention Time (min)	Area	% Area	Height
1	Peak1	11.238	6453038	21.39	421259
2	Peak2	17.932	8813518	29.21	389924
3	Peak3	18.890	8444232	27.99	346020
4	Peak4	31.622	6461770	21.42	151025

Column: Chiralpak® IC
Eluent: hexane/isopropanol/acetone (95/4/1)
Flow rate: 1 mL/min
Detection: UV 220 nm

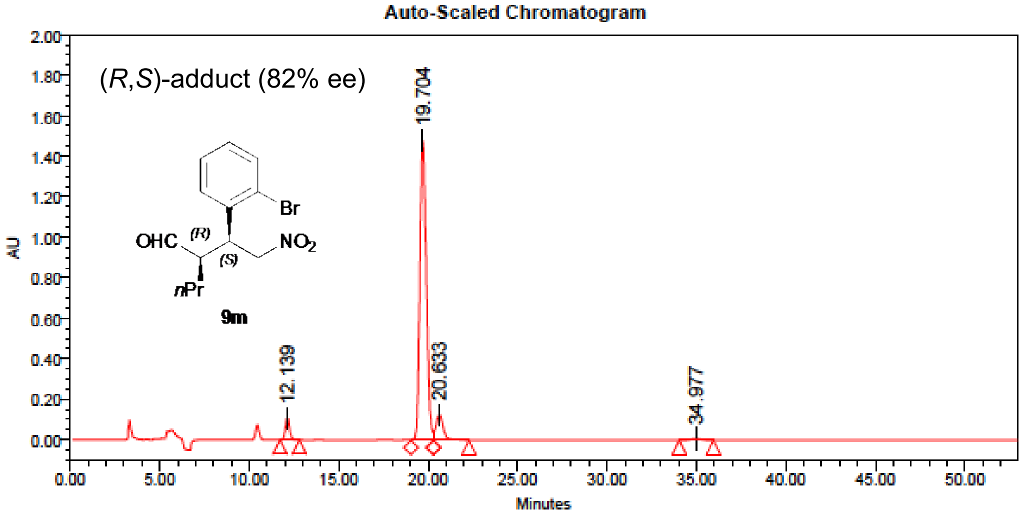

Processed Channel:

	Peak Name	Retention Time (min)	Area	% Area	Height
1	Peak1 2487Channel 1	12.139	1798995	4.23	108907
2	Peak2 2487Channel 1	19.704	36874281	86.61	1480248
3	Peak3 2487Channel 1	20.633	3555275	8.35	124551
4	Peak4 2487Channel 1	34.977	345501	0.81	7540
